# Single-cell decoding of drug induced transcriptomic reprogramming in triple negative breast cancers

**DOI:** 10.1186/s13059-024-03318-3

**Published:** 2024-07-18

**Authors:** Farhia Kabeer, Hoa Tran, Mirela Andronescu, Gurdeep Singh, Hakwoo Lee, Sohrab Salehi, Beixi Wang, Justina Biele, Jazmine Brimhall, David Gee, Viviana Cerda, Ciara O’Flanagan, Teresa Algara, Takako Kono, Sean Beatty, Elena Zaikova, Daniel Lai, Eric Lee, Richard Moore, Andrew J. Mungall, IMAXT Consortium, Marc J. Williams, Andrew Roth, Kieran R. Campbell, Sohrab P. Shah, Samuel Aparicio

**Affiliations:** 1 Department of Pathology and Laboratory Medicine, University of British Columbia, Vancouver, BC Canada; 2 Department of Molecular Oncology, British Columbia Cancer Research Centre, Vancouver, BC Canada; 3 Computational Oncology, Department of Epidemiology and Biostatistics, Memorial Sloan Kettering Cancer Center, New York, NY USA; 4 Irving Institute for Cancer Dynamics, Columbia University, New York, NY USA; 5 Canada’s Michael Smith Genome Sciences Centre, BC Cancer, Vancouver, BC Canada; 6 CRUK Grand Challenge IMAXT Team, Cambridge, UK; 7 Lunenfeld-Tanenbaum Research Institute, University of Toronto, Toronto, ON Canada; 8 Department of Molecular Genetics, University of Toronto, Toronto, ON Canada

**Keywords:** PDX, Single-cell RNA sequencing, DLP+ single-cell sequencing, Cisplatin treatment, In-cis/in-trans genes, Sensitive/resistant clones, Clone aware analysis

## Abstract

**Background:**

The encoding of cell intrinsic drug resistance states in breast cancer reflects the contributions of genomic and non-genomic variations and requires accurate estimation of clonal fitness from co-measurement of transcriptomic and genomic data. Somatic copy number (CN) variation is the dominant mutational mechanism leading to transcriptional variation and notably contributes to platinum chemotherapy resistance cell states. Here, we deploy time series measurements of triple negative breast cancer (TNBC) single-cell transcriptomes, along with co-measured single-cell CN fitness, identifying genomic and transcriptomic mechanisms in drug-associated transcriptional cell states.

**Results:**

We present scRNA-seq data (53,641 filtered cells) from serial passaging TNBC patient-derived xenograft (PDX) experiments spanning 2.5 years, matched with genomic single-cell CN data from the same samples. Our findings reveal distinct clonal responses within TNBC tumors exposed to platinum. Clones with high drug fitness undergo clonal sweeps and show subtle transcriptional reversion, while those with weak fitness exhibit dynamic transcription upon drug withdrawal. Pathway analysis highlights convergence on epithelial-mesenchymal transition and cytokine signaling, associated with resistance. Furthermore, pseudotime analysis demonstrates hysteresis in transcriptional reversion, indicating generation of new intermediate transcriptional states upon platinum exposure.

**Conclusions:**

Within a polyclonal tumor, clones with strong genotype-associated fitness under platinum remained fixed, minimizing transcriptional reversion upon drug withdrawal. Conversely, clones with weaker fitness display non-genomic transcriptional plasticity. This suggests CN-associated and CN-independent transcriptional states could both contribute to platinum resistance. The dominance of genomic or non-genomic mechanisms within polyclonal tumors has implications for drug sensitivity, restoration, and re-treatment strategies.

**Supplementary Information:**

The online version contains supplementary material available at 10.1186/s13059-024-03318-3.

## Background

The concept of gene expression plasticity and evolutionary fixation is fundamental to understanding how cell populations adapt to rapid environmental changes in a growing tumor. As gene expression includes a significant stochastic component, resulting in cell-to-cell variations in mRNA, the sources of this variability include fluctuations in the expression of individual genes and even genetically identical cells can be very different [1–7]. Studies in cell lines and model systems have emphasized the role of non-genomic transcriptional plasticity, either through fixation of stochastic transcriptional states and/or modulation of transcription through epigenetic regulation of transcription. Drug mechanisms and the duration and speed of onset of drug action likely influence the contributions of genomic and non-genomic encoding of transcriptional states. This is emphasized in the discoveries of rare drug-tolerant cells and persister cells in the cell populations of untreated cancers [5, 6, 8, 9]. In most instances these approaches have not observed genomic differences, potentially reflecting the speed of action and drug mechanisms studied. However, not all studies have systematically sequenced single cells, especially to detect somatic gene dosage variants. Copy number structural variants have greater potential to shape the fitness landscapes of resistance in cancers with genomic instability, because single mutations can affect the gene dosage and transcription levels of potentially hundreds of genes [10, 11], creating a potentially large fitness landscape for drug selection. During the evolution of genomically unstable tumors, copy number differences resulting in gene dosage differences between clones may have an extensive effect on transcription and contribute to heritable proportional gene expression differences between clones [10–13]. In triple negative breast cancer (TNBC), gene copy number changes are very abundant and have a major influence on the expression landscape [10, 13–15] and are associated with the clinical biology of these cancers.

Few studies have been structured to separate the genome effects of somatic copy number variants from non-genomic transcriptional plasticity. A recent analysis of a different process, colorectal cancer metastasis, has emphasized that in that cancer type, there is only minor variation in genotypes between primary and metastatic colorectal tumors despite ongoing background mutation, implying the primary tumor landscape is under strong selection for stability. In this case, non-genomic transcriptional variation accounts for the majority of the phenotype differences [16, 17]. The situation in breast cancers is different, where much greater variation in copy number genotypes has been observed among metastases. Bulk WGS methods can usually only detect differences once clonal selection and fixation has occurred and are thus not well suited to analyzing the dynamics of clonal and non-clonal selection. Thus analysis of the potential contributions of gene dosage mutations and non-genomic transcriptional variation in response to chemotherapy requires single-cell analysis of serially sampled populations. The utilization of single-cell DNA and RNA sequencing techniques in solid tumors has facilitated the phylogenetic reconstruction of tumor lineages [14, 18, 19]. It has also resolved rare subpopulations [20, 21] and offered insights into the phenotypes of stromal and tumor cells in various cancers [22–25]. The majority of these publications focused on either DNA or RNA exclusively. These studies did not explore the analysis of both RNA and DNA at the single-cell level within the same sample, nor did they examine changes in gene dosage affecting gene expression at the clonal level. Using a scaled single-cell genome sequencing technology and population genetic fitness models, we have recently shown that copy number genotype differences among subclones of TNBC can be associated with platinum resistance [26]. Here we have exploited this observation to dissect the dynamics of clone-associated and clone-independent transcription during the onset and reversion of platinum drug resistance sampling 53,641 single-cell transcriptomes of serially platinum-treated TNBC patient derived xenografts over a 2.5 years interval. We show that even within a tumor, different subclones may exhibit quite different transcriptional dynamics to drug treatment and withdrawal, reflecting the degree of clonal fixation.

## Results

### Copy number variation shapes PDX single-cell transcriptomes in proportion to genomic instability

**Fig. 1:**
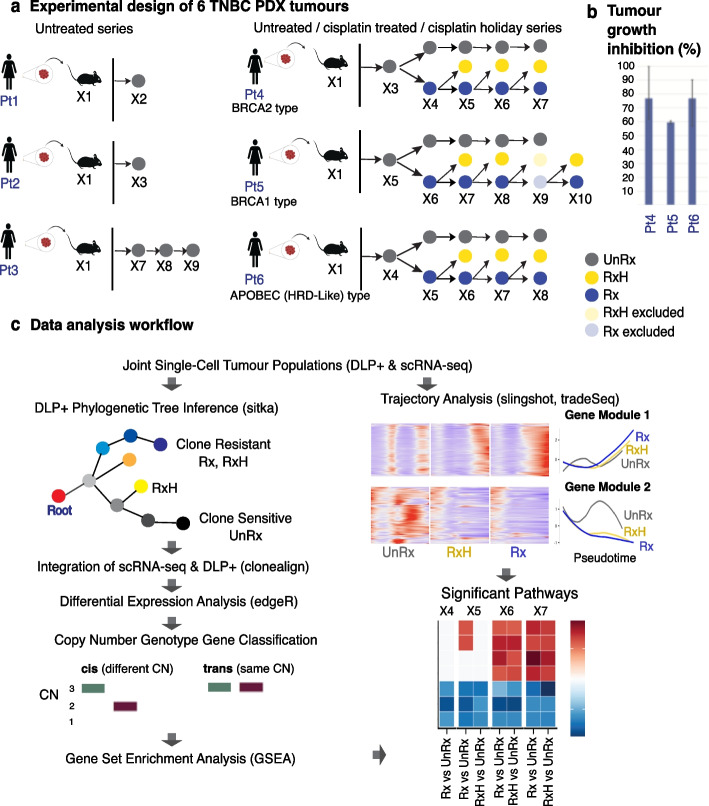
Workflow of experimental and study design for tracking drug induced transcriptome reprogramming. **a** Tumor biopsies from 6 TNBC patients were transplanted in immunodeficient mice. Three TNBC untreated time-series (Pt1-3) and three cisplatin-treated time-series (Pt4-6) with its counter drug holiday samples. UnRx: Grey-untreated, Rx: Blue-cisplatin treated, RxH: Yellow-cisplatin drug holiday (kept untreated for that cycle of treatment). The treated, drug holiday samples at X9, Pt5 were excluded from analysis due to its sample qualities. **b** Tumor growth inhibition graphs for Pt4, Pt5 and Pt6. **c** Data analysis workflow, including phylogenetic tree inference using DLP single-cell copy number profiles from previously published work [27], followed by clonal alignment from DLP copy number to RNA-seq gene expression with clonealign [28]. Differentially expressed genes are then classified into in-cis and in-trans based on overlapping genomic regions, and based on the positive or negative directions of copy number tendency and gene expression trend (upregulated or downregulated). Significant pathways were determined by applying gene set enrichment analysis. Pseudotime analysis identified genes with significant change in expression along the trajectories of evolution through drug treatment

We set out to measure the copy number aberration (CNA) associated and copy number independent transcriptional variation in subpopulations of patient-derived xenografts passaged over months to years under neutral (no drug intervention) conditions (UnRx) and with parallel cisplatin treatment (Rx) and cisplatin holiday (RxH) conditions (Fig. 1, Methods). We have previously measured CNA-associated clonal fitness using a Wright Fisher model for treated and untreated passages and validated the measured fitness with repeated mixture re-transplant experiments [26]. Here, we established a new dataset of joint tumor cell population measurements utilizing DLP + single-cell genome sequencing (scWGS) to measure clonal structure determined by CNA and scRNA-seq sampled from single cells in the same population from 6 triple negative breast cancer patient-derived xenograft series (Fig. 1, Additional file 1: Figure S1), including 3 patients where serial platinum treatment samples were obtained. We captured 53,641 high-quality transcriptomes (post-filtering), representing 28,074 cells from untreated tumors (Pt1-6), 14,111 cells from cisplatin-treated tumors (Pt4,5,6), and 11,456 cells from cisplatin drug holiday tumors (Pt4,5,6, Table 1, Additional file 2: Table S1).

**Table 1:** Types and numbers of single-cell RNA-seq data generated for this study

Current label of TNBC PDX	Previous label [26, 29]	Condition	No. of scRNA-seq passages	No. of scRNA-seq cells sequenced	No. of filtered cells for analysis	Percentage of cells assigned to clones
Pt1	SA501	Untreated (UnRx)	1	7,799	2,600	75.9%
Pt2	SA530	Untreated (UnRx)	1	3,021	1,207	94.6%
Pt3	SA604	Untreated (UnRx)	3	7,369	3,685	84.6%
Pt4	SA609	Untreated (UnRx)	5	22,628	12,109	99.9%
		Cisplatin (Rx)	4	12,281	8,001	100%
		Cisplatin holiday (RxH)	3	9,857	6,828	100%
Pt5	SA535	Untreated (UnRx)	5	9,076	4,371	99.9%
		Cisplatin (Rx)	4	7,551	3,322	94.4%
		Cisplatin holiday (RxH)	3	5,801	2,284	96.8%
Pt6	SA1035	Untreated (UnRx)	5	16,803	4,104	76%
		Cisplatin (Rx)	4	10,970	2,788	80.6%
		Cisplatin holiday (RxH)	3	9,147	2,344	98.0%
Total untreated Pt1-Pt6		Untreated (UnRx)	20	66,696	28,074	92%
Total Cisplatin Pt4-Pt6		Cisplatin (Rx)	12	30,802	14,111	95%
Total Cisplatin-hol iday Pt4-Pt6		Cisplatin holiday (RxH)	9	24,805	11,456	98.9%
Total Pt1-Pt6		All conditions	41	126,556	53,641	94.2%

The columns show: (1) the patient label Pt1-6; (2) the previous labels used in other publications; (3) the treatment conditions: untreated, treated with cisplatin and treated with cisplatin followed by holiday; (4) the number of scRNA-seq passages; (5) the number of scRNA-seq cells initially sequenced; (6) the number of cells after applying the mouse contamination, quality control and doublet filters (see Methods)—all these cells were used as input to clonealign and in the pseudotime analysis; (7) the percentage of cells that were assigned to clones by clonealign after additional filtering and clone assignment (see Methods). See Additional file 2: Table S1 for details of each individual sample

**Fig. 2:**
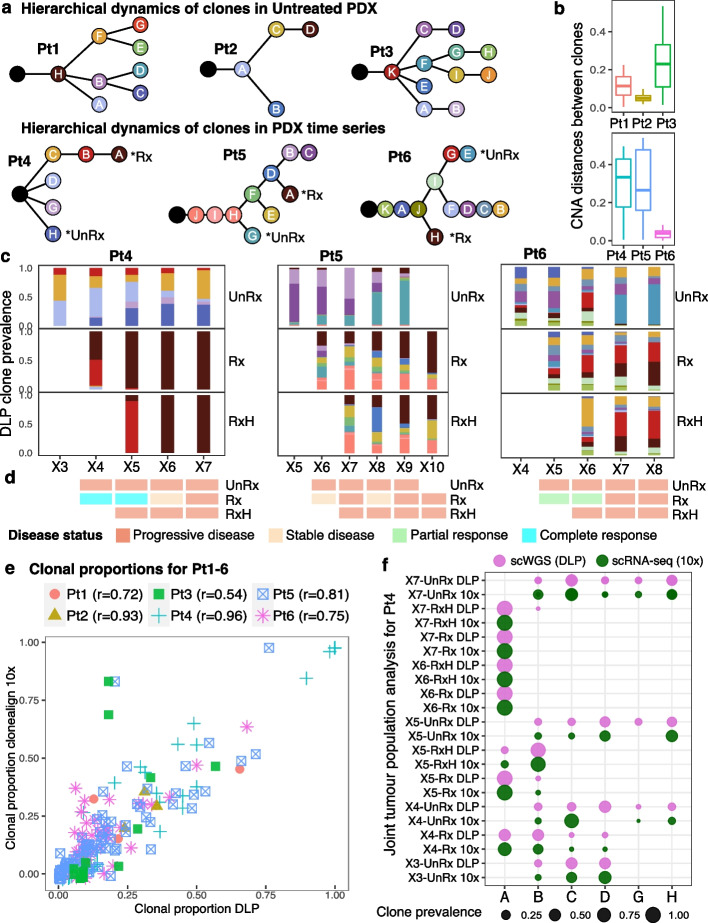
Assignment of structural-copy number clone structure to scRNA-seq. Summary results of phylogenetic trees and clone alignment correlations. **a** Inferred phylogenetic trees of three untreated PDX patients Pt1-3, and three drug treatment time-series data Pt4-6. Resistant, sensitive cell clones were developed at different branches far from each other. (*) denote high fitness coefficient clones in each branch based on the previously published work [26]. **b** Manhattan copy number distances between median copy number profiles of paired clones in each series denote the variance level in the copy number DLP tree. **c** Clonal evolution of cells with/without drug treatment across time. **d** Disease classification at each time point and treatment status for Pt4-6. **e** Each point shows the clonealign-inferred scRNA-seq clonal proportion (*y*-axis) versus the corresponding DLP+ clonal proportion (*x*-axis) for each clone in each sample. Each patient is denoted by a different shape. Pearson correlation coefficients for each patient are reported at the top. **f** Pearson correlation between clonal proportions in DLP, and inferred clonal proportions in 10x – scRNA-seq expression at the same passages from patient Pt4 using clonealign. Strong positive correlation demonstrates that clonealign is able to provide accurate alignment

To assign scRNA-seq transcriptomes to clones, we first generated CNA phylogenetic trees with a Bayesian [27] method from DLP+ single-cell genome sequencing of 6 patient PDX lines, to determine the genome fraction occupied by TNBC breast cancer clones. For each patient series, we identified CNA clonal populations from major clades (Fig. 2a), and copy number profiles for each clone (Fig. 2b, c, Additional file 1: Figures S2-S5). Reflecting the background genomic diversity of breast cancer patients [10], the number of major clones varied from 4 (Pt2) to 11 (Pt3,6), while the proportion of genome altered by CNA (Manhattan distance) was < 0.06 average CNA distance for Pt2 and Pt6, and > 0.2 for Pt3,4,5, reflecting the natural variation of CNA clonal composition of TNBC [26]. Among three patient lines treated serially with platinum (Pt4,5,6, Fig. 1), Pt4 exemplifies a low complexity (6 clones) and intermediate fraction of altered genome (median = 0.33, sd = 0.16), Pt6 a high clone complexity (11 clones) and low fraction of altered genome (median = 0.04, sd = 0.02), whereas Pt5 exhibited high clonal complexity (10 clones) and high altered genome fraction (median = 0.27, sd = 0.16), Fig. 2a, b.

We have previously measured the clonal fitness of Pt4,5,6 under different passaging conditions [26]. Briefly, the fitness coefficient denotes the relative growth potential rate (increased or attenuated) of a given clone over time and was quantified using a Wright-Fisher inspired Bayesian probabilistic framework named fitClone. The fitness coefficient results from this previous analysis for patients Pt4-6 are included in Additional file 1: Figures S3b, S4b, S5b and Additional file 2: Table S2. In addition to fitness quantification, we have also previously confirmed clonal fitness by experimental mixing and serial re-transplantation experiments [26]. In the present study, clones from the treatment series with high fitness coefficient were labeled as “Rx” (resistant to drug), and clones from the untreated series with high fitness coefficient were labeled as “UnRx” (sensitive to drug, Fig. 2a, Additional file 2: Table S2).

We note that for all the treatment patients Pt4-6, a fitness landscape inversion occurs, such that minor clones present at early untreated stages become prevalent under treatment, identifying them as resistant clones in the treated samples. Resistant clones arise on different clades (i.e., branch of the phylogenetic tree) than clones in untreated samples (Fig. 2a), the latter exhibiting near neutral fitness as determined by a Wright-Fisher model [26]. In Pt4, clone B appears as a minor population in the untreated samples and evolves through minor gene dosage mutations into clone A, which exhibits a clonal sweep, becoming the resistant and only clone after two or more rounds of cisplatin treatment. In Pt5, clones B and C diminish in prevalence in the untreated samples, but their parent, clone D, evolves into clone A, a resistant clone under treatment, which is on a very different clade than the sensitive clone G that increases in abundance in UnRx. In Pt6, clone G, the parent of the sensitive clone E, expands in the treated samples, but the fittest resistant clone, clone H, is again on a very different clade than the sensitive clone E. Thus clones originating from different clades in the phylogenetic tree based on CNA mutations have different fitness under UnRx and Rx or RxH samples, as previously shown [26].

Using mRECIST criteria, *complete response*, *partial response* < 50% tumor shrinkage, *stable disease* 0% tumor shrinkage or *progressive disease* (Fig. 2d and Extended Data Fig. 3 of ref. [26]), we observed initial physical tumor responses with onset of resistance within 2 cycles, indicating that early clonal responses were also mirrored by physical tumor volume response.

Next, we mapped scRNA-seq from cells drawn from the same tumor cell populations to the CNA clones defined above, using clonealign [28], which probabilistically assigns CNA clone identity to scRNA-seq transcriptomes, assuming that an increase in the copy number of a gene will result in a corresponding increase in that gene’s expression and vice versa (Methods, Additional file 2: Table S3). The inferred clonal prevalences (Fig. 2e, f) of cells in scRNA-seq populations were highly concordant with the clonal prevalences of cells in DLP+ populations (Fig. 2c), Pearson correlation coefficient *r* > 0.72 for all but 1 patient, overall correlation *r* = 0.86, *p*-value < 0.05 for all patients, average correlation in three treatment series was greater than in three untreated series when cells were under treatment *r* = 0.84 + −0.11, *p*-value < 0.05.

**Fig. 3:**
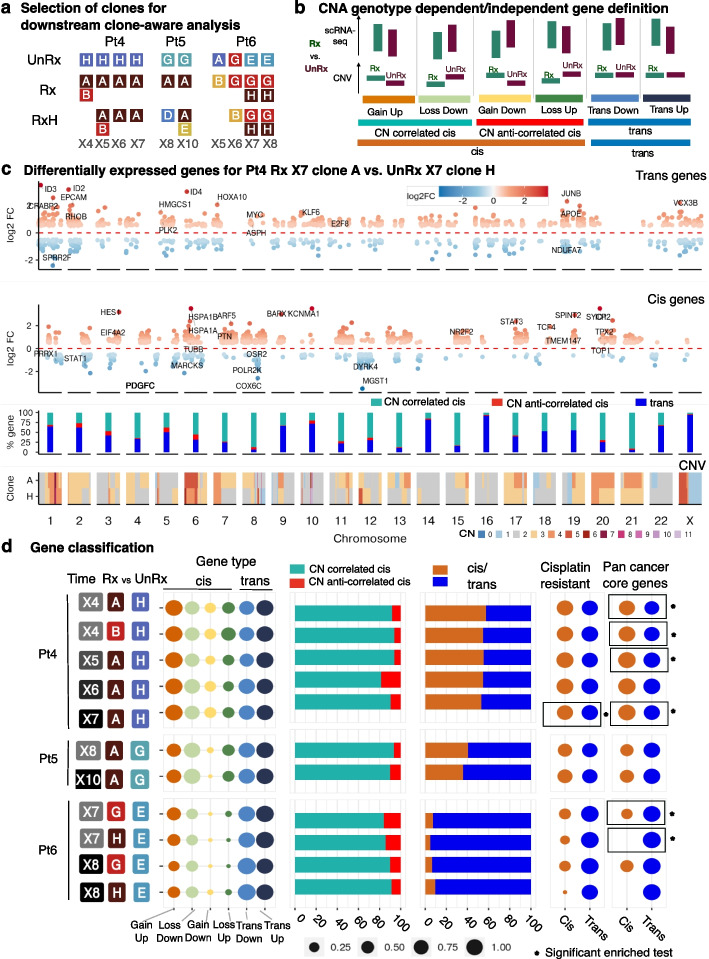
In-cis and In-trans gene proportions and gene set memberships. **a** For Pt4-6, the clones at the same passage that were the fittest or most abundant and had at least 100 scRNA-seq cells were selected for further clone-aware analysis. Examples of comparisons include: Pt4 X4 Rx:A vs. UnRx:H, or Pt5 X10 RxH:E vs. UnRx:G. **b** Gene classification: genes are divided into in-cis (located in regions of CN differences) and in-trans (no CN differences). In-cis gene expression is denoted as CN correlated cis (concordant with CNA change) or CN anti-correlated (discordant with CNA change). **c** Differentially expressed genes for Pt4 between resistant clone A in Rx passage X7 versus sensitive clone H in UnRx passage X7, classified into “in-trans” (top panel) and “in-cis” genes (second panel). Red and blue gradient colors denote the degree of log2 fold change (FC) in positive and negative directions. Each dot is one significant DE gene with selected conditions abs(log2FC) > 0.5, FDR < 0.01, *p* value < 0.05. Third panel: % in-cis CN correlated—light green, CN anti-correlated—red, and in-trans genes—blue color per chromosome. Bottom panel: median copy number profile of genomic regions for the two clones from DLP+ results. **d** Differentially expressed genes between resistant clones versus sensitive clones in Pt4-6 were classified into different gene types. The dot size represents the proportion of each gene type based on the definition in **b**. Gene set membership: mapping of in-cis and in-trans genes to 2 reference gene sets: our curated cisplatin resistance gene set from the literature and Pan cancer core genes set [31]. Rectangles with * show significant enrichment of reference gene sets - results of statistical tests using GSEA [32] with *p*-adj values < 0.05

Next, having established CNA clone identity of transcriptomes, we were interested in exploring the transcriptional change between distinct clones or distinct treatment status at the same time point (passage). Therefore, we investigated differential transcription among same-passage clones that had the highest fitness coefficient [26] in different treatments (e.g., treated versus untreated, or drug holiday versus untreated), and/or were the most abundant at each time series sample (see Fig. 3a). For this purpose, we used edgeR, which implements a negative Binomial model to determine genes whose expression changes between conditions [30] (Methods). The gene expression change strongly correlated with the copy number change (Pearson correlation coefficient > 0.95, Additional file 1: Figure S6), which is expected given clonealign’s model of gene expression – copy number correlation, and consistent with previous findings across many tumor types including breast cancer [10, 11].

Having obtained the differentially expressed (DE) genes for an inferred clone pair (e.g., the comparison in Fig. 3c for Pt4, passage X7, treated inferred clone A versus untreated inferred clone H), we next divided the DE genes based on whether they are encoded in genomic regions of copy number difference between clones: we refer to in-cis, where the copy number values are different between the two clones compared, and in-trans, where the copy number values are the same (Fig. 3b). Precisely, first we extracted the genomic coordinates of each DE gene, and we mapped its genomic coordinates to the most overlapping genomic region in the DLP+ copy number data. Then, we calculated the median copy number across all cells in each clone, and compared the two medians. If they were different, then the DE gene is defined as an in-cis gene; otherwise, it was defined as an in-trans gene. In-trans regions exhibited a larger proportion of up- than downregulation differences across treated and untreated tumor (39.5% sd = 8.5% of the upregulated in-trans genes on average across patients Pt4,5,6 as compared to 26.9% sd = 15.9% downregulated in-trans, KS test *p*-value = 0.03, Fig. 3c, top panel). Regions identified as in-cis exhibited the expected (copy-number correlated) directional tendency of differential expression (example Pt4, Fig. 3c, lower track), with a small proportion of copy-number anti-correlated in-cis transcripts regulated against the direction of clonal copy number difference (example Pt4, Fig. 3c, second track). Copy-number correlated and anti-correlated in-cis differential expression was evident over all sizes of CNA from sub-chromosomal regions (e.g., Pt4, Chr1, Chr18, Fig. 3c, second track) to whole chromosomes (Pt4 Chr 7, Fig. 3c, second track, Pt5,6, Additional file 1: Figure S7).

We identified copy-number correlated and copy-number anti-correlated differential expression and quantified the proportionality of in-cis and in-trans DE transcripts across the selected clones (Fig. 3b, d, Additional file 1: Figure S8, Additional file 2: Tables S4 and S5). The fraction of CNA genotype associated in-cis transcripts varied from 5.1 to 57.4% of the total number of DE genes in Pt4,5,6, reflecting the variance in the size and number of genomic copy number differences (Fig. 2b, Additional file 1: Figures S3a, S4a, and S5a) between clones for each patient. The proportion of in-trans transcripts up- and downregulated between pairs of clones was similar in all untreated patients (Additional file 1: Figure S8). However the DE variance of in-trans transcripts was greater (in-trans upregulated, sd = 4%, downregulated sd = 3.05%) than in-cis region transcript (in-cis upregulated sd = 0.49%, in-cis down sd = 1.22%; Levene’s test, *p*-value < 0.05 for upregulated genes logFC values between in-cis, in-trans genes in Pt4, Pt5, and for downregulated genes in Pt4, Pt6). In contrast, a small proportion of in-cis transcripts (3.1%, sd = 2.2%, Fig. 3d) in each of the Pt4,5,6 patient treatment series exhibited in-cis copy-number anti-correlated differential expression, indicating a relatively constant fraction of transcripts escaping gene segment copy number dosage effects. This copy-number anti-correlated DE transcript proportionality was stable across time points (Pt4 sd = 2.8%, Pt5 sd = 0.7%, Pt6 sd = 0.2%). Copy-number anti-correlated transcripts have been noted in several studies [10, 11, 33]. Copy-number anti-correlated transcripts may arise from small CNAs that were missed (for example the PDGFC gene, see Fig. 3c, second panel, chromosome 4), or by mis-calling the locations of CNA segment boundaries (on average 11.7% of the copy-number anti-correlated transcripts overlap with the copy number bin boundaries: 11.6% for Pt4, 13.4% for Pt5 and 10.6% for Pt6). However, multiple genes that span the same CN segment are likely to be true copy-number anti-correlated genes (see Fig. 3c, second panel, chromosome 2), suggesting that a small proportion of transcripts are biologically counter regulated.

To understand the dosage effect of copy number in treatment response genes compared to untreated lines, we examined further the following classes of DE genes in the in-cis and in-trans categories: untreated comparisons: (i) UnRx vs. UnRx, and treated comparisons including (ii) Rx expanding clones vs. UnRx, (iii) Rx shrinking clones vs. UnRx, (iv) RxH vs. UnRx (Additional file 1: Figure S9). Overall, the in-cis proportion of the treated comparisons are lower than in untreated comparisons in Pt5, Pt6 (mean +− sd = 37.55 +− 3.47 for treated and 58.52 +− 3.09 for untreated in Pt5, and 10.93 +− 3.63 for treated vs. 31.08 +− 5.02 for untreated in Pt6), showing that the majority of DE gene regulation in untreated clones associates with in-cis genes, i.e., regions of copy number difference. However, in contrast, in Pt4 serial transplants, in-cis genes contribute less in treated versus untreated comparisons (mean +− sd = 53.57 +− 1.12 for treated and 41.84 +− 14.1 for untreated). This difference across patients may be explained by the dosage effects in Pt4 due to gene dosage mutations conferring very strong fitness to platinum treatment (Fig. 2b).

Next, we asked whether DE transcripts reflected possible resistance mechanisms. Through GSEA analysis using curated cisplatin resistance genes (Additional file 2: Table S6) and published cancer gene sets [34], we found several clones that exhibited statistically significant enrichment of known pan cancer core gene set [34], whereas only one clone pair exhibited enrichment of known cisplatin resistance genes (Fig. 3d, Additional file 2: Table S6), indicating that additional resistance pathways remain to be discovered.

### Clonal and non-clonal transcriptional responses to therapy reveal intratumoral heterogeneity

Having mapped in-cis (CNA-clonal) and in-trans region single-cell transcription, we examined the transcription modulation associated with cisplatin treatment and with treatment withdrawal from in-parallel treatment holiday transplants of previously drug exposed tumors [26]. This partially mimics cyclical dosing of patients with platinum.

**Fig. 4:**
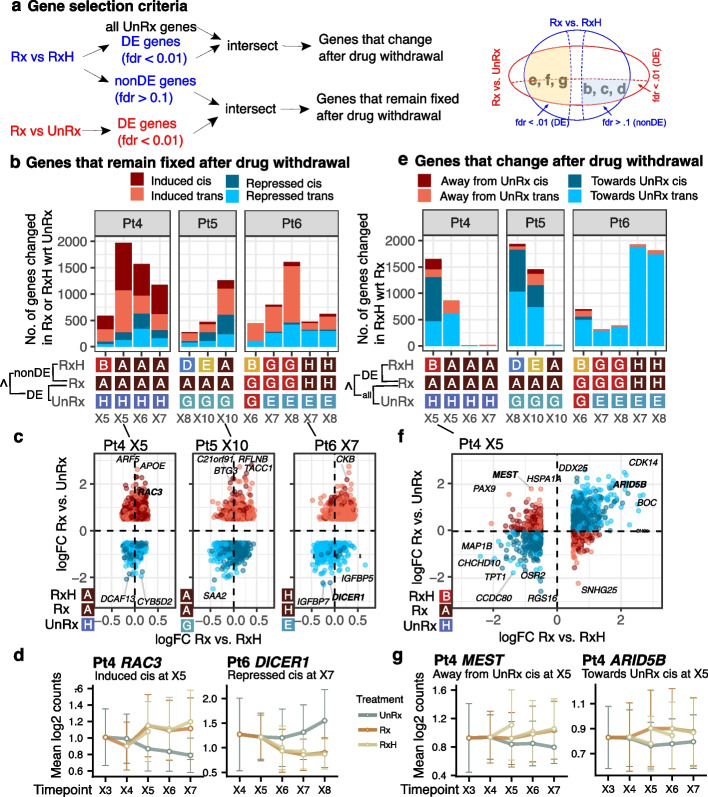
Gene level dynamic changes. **a** Schematic showing our gene selection criteria for Pt4-6: genes in **b**, **c**, **d** are differentially expressed (DE, fdr < 0.01, |log2 fold change| =|logFC|> 0.5) at Rx vs. UnRx and non-DE (fdr > 0.1) at Rx vs. RxH; genes in **e**, **f**, **g** are DE at Rx vs. RxH and intersected with all the genes in Rx and UnRx. The clones in Fig. 3a were used for comparisons. **b** Number of in-cis or in-trans treatment induced (logFC > 0.5) and repressed (logFC <− 0.5) genes for Pt4-6, when comparing the clones listed in the bottom panel. Genes are selected as explained in panel **a**. The in-cis and in-trans gene annotation uses the copy numbers at Rx and UnRx. **c** Scatter plots for logFC of Rx vs. UnRx (*y* axis) against logFC of Rx vs. UnRx (*x* axis) for the comparisons with the largest numbers in panel **b**. Each point is a gene. The color legend is the same as in panel **b**. **d** Two examples of induced and repressed genes for Pt4 and Pt6. **e** Number of in-cis or in-trans holiday diverged genes, split in two main categories: “towards UnRx” if logFC of Rx vs. RxH has the same sign as logFC of Rx vs. UnRx, and “away from UnRx” otherwise. The comparisons are as explained in panel **a**. The in-cis and in-trans gene annotation uses the copy numbers at Rx and RxH. **f** Scatter plots for logFC of Rx vs. UnRx (*y* axis) against logFC of Rx vs. UnRx (*x* axis) for Pt4 X5 (UnRx:H, Rx: A, RxH:B). Each point is a gene. The color legend is the same as in panel **e**. **g** Two examples of diverged genes away from (left) and towards (right) UnRx

Investigating the scRNA-seq transcripts that are stable (i.e., not differentially expressed, fdr > 0.1) between treatment and holiday transcriptomes, but expressed differentially from the untreated state (fdr < 0.01, |log2 fold change| =|logFC|> 0.5, Fig. 4a, b), we observed that in all patients drug treatment induces more transcripts than are repressed (Fig. 4a–d and Additional file 1: Figure S10a, Additional file 2: Table S7), ranging from ~ 250–2000 per comparison. The proportion of genes exhibiting increased (induced) stable expression under treatment (e.g., RAC3, Pt4, Fig. 4d) was higher (66%) than stably decreased (repressed) transcripts (e.g., DICER1 Pt6, Fig. 4d) in all series except for the last time points of Pt5 and Pt6 (43 and 35%, respectively). Overall, the proportions of in-cis versus in-trans genes that contribute to the stable expression state of drug exposed cells (52–61% for Pt4, 20–45% for Pt5 and 0–12% for Pt6, Fig. 4b, Additional file 1: Figure S11a) were generally higher than those of untreated passages (Fig. 3d).

We asked how transcription changed over time in the same clones, where these could be observed in sufficient abundance over multiple passages. We have previously shown that Pt4 exhibited the strong genotypic clonal selection for platinum resistance, with eventual domination (by passage X6,7, Fig. 2c) of a single resistant clone labeled A. In Pt4 comparisons of the untreated high fitness clone H, which is in a distant branch to strongly platinum-resistant clone A (Fig. 2a), suggested a gradual decline in differential expression over time (Fig. 4b). However, differential expression with untreated clone C, which is in the same clade as clone A, showed fewer transcriptional differences (Additional file 1: Figure S11b). In Pt6 where clone E (untreated) and G (resistant to platinum) are sister clones, comparison of E with G and comparison of E with H, a distant but also platinum-resistant clone, both showed passage decline in differential expression (Fig. 4b). Taken together, this indicates gradual transcriptional adaptation to repeated platinum exposure, even within resistant clones.

We next asked whether and to what extent treatment withdrawal influenced the clonal drug resistance transcriptional states (Fig. 4a, e–g and Additional file 1: Figure S10b). In treatment holiday intervals, the majority (79% over all samples) of the DE transcription reverts in the direction of untreated, indicating a collapse back to the earlier state. Notable exceptions are later passages of Pt4 where the tumor cell population exhibited a full CNA clonal sweep of clone A (strongly platinum resistant genotype) at the last two passages, which we have previously shown was the fittest under platinum treatment. An extreme example of loss of transcriptional plasticity after clonal fixation was observed for Pt4, where the later passages X6, X7 of platinum exposure resulted in a monoclonal population [26]. In this case, few reversible genes were observed (only 15 genes at X6 and 19 genes at X7, all in-trans, whereas 2513 genes were found at X5, Additional file 2: Table S7), consistent with a fixed genomic landscape of drug resistance (Fig. 4e–g), with minimal transcriptional plasticity (Additional file 1: Figure S11c). A similar observation was made for clone A of Pt5, where the transcriptional landscape is fixed at the last passage (X10). On the contrary, clonal sweeps of resistant clones were not detected in Pt6 (see also Fig. 2c), and similarly a large number of reversible genes were found at all time points (> 695 for Pt6, Fig. 4e, > 70% by the last passage, Additional file 1: Figure S11c). This suggests that these genes develop into new states that are different from untreated and platinum treatment. Taken together, the degree of transcriptional adaptation in single CNA clones on initiation or withdrawal of treatment appears inversely related to the relative contribution of genotypic fitness to platinum.

**Fig. 5:**
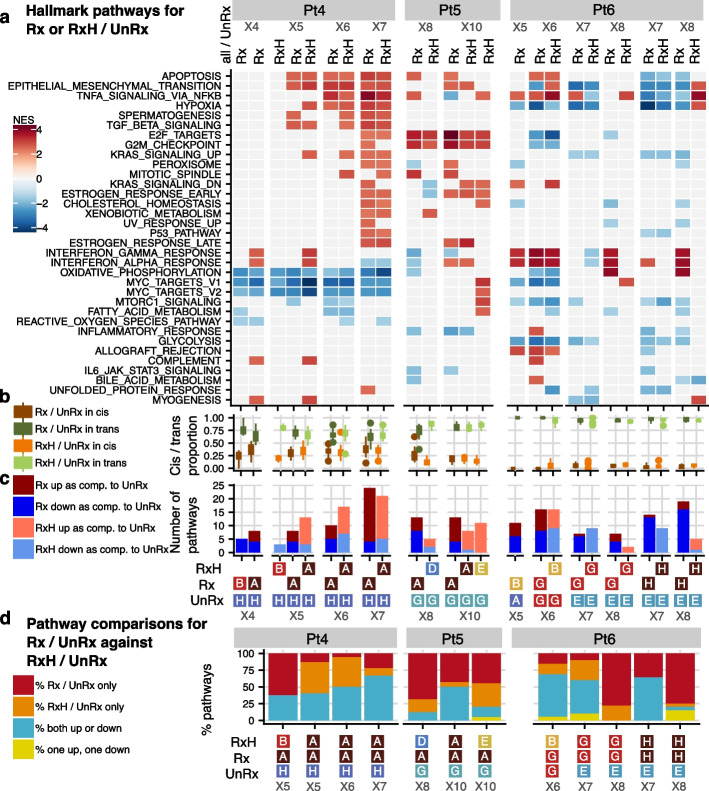
Hallmark significant pathways in Rx, or RxH versus UnRx for Pt4-Pt6. **a** Significantly enriched pathways (*p* < 0.05, vertical axis) from a ranked gene set enrichment analysis (GSEA) [32], using the Hallmark gene set collection from MSigDB [32, 35]. Each column corresponds to one comparison between a treated or holiday clone versus an untreated clone (as denoted in Fig. 3a), for a specific patient and time point. The color intensity signifies the normalized enrichment score (NES) results of enrichment analysis obtained by using all the edgeR differentially expressed genes for that comparison at FDR < 0.01 and |log2 fold change|> = 0.25. Only the common pathways that are enriched in at least three DE comparisons across all patients are shown. **b** The distribution of in-cis and in-trans pathway gene proportions for all the comparisons in **a**. **c** The number of upregulated and downregulated pathways in each column in **a**. **d** Pathway status of treated-untreated comparisons against the pathway status of their respective holiday-untreated comparisons, split into four types of changes: (i) “Rx only” include pathways that are enriched in treated, but not in holiday; (ii) “RxH only” are pathways that were not enriched in treated, but are enriched in holiday; (iii) “both same direction” are pathways that are enriched in the same direction in treated and holiday; and (iv) “both reverse direction” are pathways that are enriched in a different direction in treated and holiday samples

To define the functional pathways implicated in clone-specific and clone-independent gene expression, we mapped differentially expressed genes from contrasts between the selected clones (Fig. 3a) across temporal passaging, for drug treatment and drug withdrawal versus their untreated temporal counterparts (Fig. 5, Additional file 2: Table S8). We used pathway enrichment tests and Hallmark reference gene sets to highlight the most significant pathways that are related to cancer. We found that the number of regulated pathways for treated versus untreated clones increased with serial exposure for all Pt4,5,6 (from 8 at X4 to 24 at X7 for Pt4, from 8 at X6 to 13 at X10 for Pt5 and from 11 at X5 to 19 at X8 for Pt6, see also Fig. 5c). More significant pathways (*p* < 0.05) are activated at later time points, relative to untreated counterparts.

While Pt4 and Pt5 displayed more upregulated pathways than downregulated pathways, especially after several rounds of treatment, Pt6 showed the opposite trend in the last two time points, showing heterogeneity in transcriptional responses across patients. This is consistent with previous work [26] and could be because of different DNA repair deficiency phenotypes of Pt4 and Pt5 as *BRCA2* and *BRCA1* types respectively, while Pt6 [36] was exhibiting *APOBEC-BRCA1* germline mutational signatures. Interestingly, different clones of the same sample may encode common as well as individual pathways, as observed for example in Pt5 X10, where holiday clones A and E display four common pathways and ten individual pathways, when compared against the corresponding untreated sample (Fig. 5a).

The in-cis and in-trans composition of the genes in all the hallmark pathways taken together for each comparison, was consistent with the proportions discussed earlier in Fig. 3 (mean percentage of in-cis genes 33.4, 19.4 and 3.7% for Pt4,5,6, respectively, Fig. 5b). In-cis gene mean proportions for each individual pathway are also similar (35.1, 17.2 and 4.6% for Pt4,5,6, respectively, Additional file 1: Figure S12), with a few notable exceptions. Notably, the KRAS Signaling Down pathway, which contains 62.5% of the in-cis genes from Pt4, and MYC Targets V2, having 10.5% of the in-cis genes from Pt4 (see Additional file 2: Table S9 for the list of all in-cis and in-trans pathway genes), both exhibit an excess of in-cis transcripts. All pathways at the last time points contained both in-cis and in-trans genes, except for five Pt6 pathways, including TNFA Signaling Via NFKB and Interferon Alpha and Gamma Response pathways, which contained only in-trans genes.

When comparing the enriched pathways for treated versus untreated clones (Rx vs. UnRx) and for drug withdrawal versus untreated clones (RxH vs. UnRx, see the columns in Fig. 5a–c), we once again observed the clonal fixation for Pt4, with more similar pathway enrichment at X7 than at X5), but continuous differentiation for Pt6, with the pathway enrichment diverging drastically more at X8 than at X6. Pt5 also displayed no clonal fixation, but less differentiation than Pt6.

**Fig. 6:**
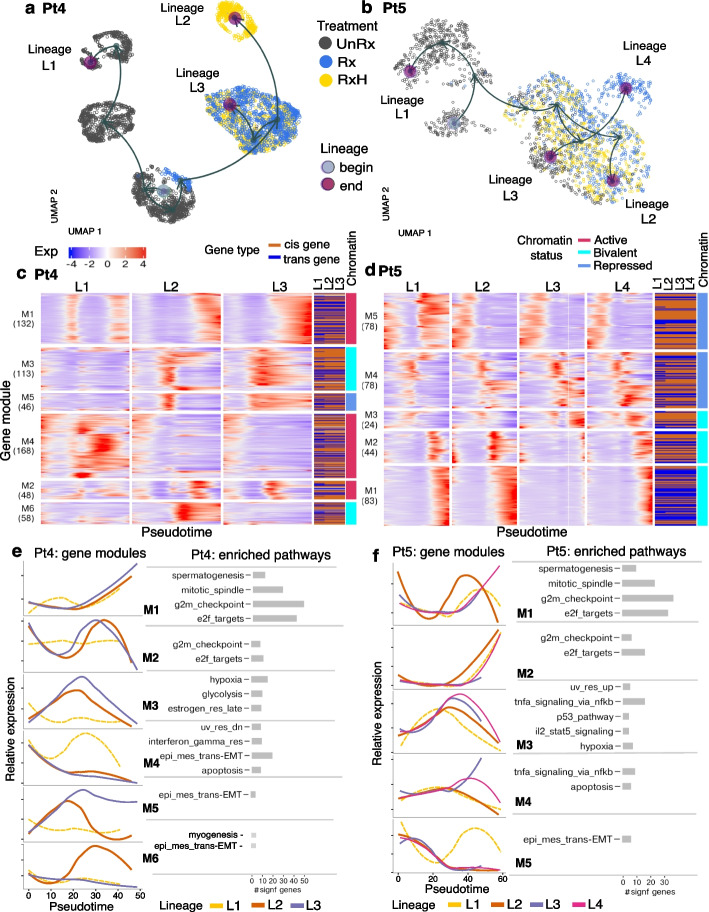
Dynamic gene regulation modules across treatment are captured using pseudotime analysis in Pt4 and Pt5. **a**, **b** UMAP visualization with individual cell lineages—output of pseudotime analysis for Pt4 (**a**) and Pt5 (**b**) coloured by actual drug treatment status (UnRx: untreated cells, Rx: drug treatment, RxH: drug holiday). Green circle denotes starting point of a lineage, red circle denotes the end point of a lineage. **c**, **d** Heatmap of gene expression across the pseudotime of different lineages for Pt4 (**c**) and Pt5 (**d**). *X*-axis: relative smoothed gene expression of each gene in each lineage, colors denoting gene expression level, blue—low expression, red—high expression. Gene types of each individual gene across different lineages, dark chocolate: in-cis, blue: in-trans gene. Chromatin status of each gene module: active—red, bivalent—cyan, repressed—blue. *Y*-axis: each row is an individual gene. Rows are grouped by regulatory gene modules (*M* denotes a gene module), i.e., M1(132) means gene module 1 and contains 132 genes. **e**, **f** Summary of relative gene expression for individual lineages in each gene module from heatmap panels **c**, **d** and list of Hallmark enriched pathways related to each gene module based on enrichment analysis gprofilers statistical tests with *P*-adj < 0.05. Bar size denotes the number of genes in each gene module that belong to significant Hallmark gene sets (M6 in panel **e** did not have any significant gene sets; therefore, we included the pathways that had the most number of genes). *x*-axis: pseudotime, *y*-axis: relative expression scaled from −2 to 2

We have previously observed that the gene expression regulation may change after withdrawal of drug (Fig. 4). Similarly, for our Pt4-6 samples, we see pathways that are enriched after drug, but not after drug withdrawal, and vice versa (Fig. 5d, “Rx / UnRx only” and “RxH / UnRx only”, respectively). For treatment induction (“Rx / UnRx only”), a maximum of 60, 65 and 76% of pathways diverged respectively in Pt4, Pt5 and Pt6. On the contrary, some pathways were enriched only in the holiday versus untreated clones (Fig. 5d, “RxH / UnRx only”), with the largest percentage occurring for Pt4 at X5 (47%). The majority of the remaining pathways displayed enrichment in the same direction (both up or both down). For Pt4 and Pt5, the largest percentage of such pathways was observed at the last time point (67 and 50%, respectively), suggesting some degree of pathway fixation over time, while for Pt6, this was observed at the second last time point (64%), suggesting that pathways are still differentiating (Fig. 5d). For Pt4,5, fewer than 5% of the pathways changed direction of enrichment, from up- to downregulated or vice versa (Fig. 5a, d), whereas for Pt6 up to 15% of the pathways changed direction by the last time point. Taken together, our data suggest that, while some pathways remain stable after drug withdrawal, most either revert to the untreated state, indicating an acquisition of transcriptional memory of prior treatment, or move into a new state distinct from treated or untreated transcriptomes (see also pseudotime analysis and Fig. 6).

Finally, we asked which cellular functions are represented by the in-cis and in-trans encoded transcription dynamics to drug exposure and withdrawal. Shared common pathways between treated cells (Rx) versus untreated cells (UnRx) across patients Pt4,5,6 include apoptosis, epithelial mesenchymal transition (EMT), hypoxia, TNF signaling, and cell cycle/checkpoint functions (Fig. 5, Additional file 1: Figure S12). EMT is known to significantly contribute to chemoresistance by converting epithelial cells into mobile mesenchymal cells and altering cell to cell adhesion as well as the cellular extracellular matrix, leading to invasion of tumor cells [37]. TGF-β signaling is known to create pre-target cisplatin resistance and is a critical cellular initiator of EMT [38, 39]. Hypoxia is known to create post-target resistance through pro-apoptotic effects and off-target cisplatin resistance [40, 41] through high expressions of *TMEM45, ID1, ID4* genes (Fig. 3c). TNFA signaling via NF-κB develops cisplatin resistance through the activation of mediators, including anti apoptotic genes [42]. This pathway was also found to exhibit reversal of expression on drug withdrawal (Fig. 5a). Some pathways exhibited patient-specific expression, including MTORC1, IL-2 STAT5 Signaling, and KRAS Signaling Down. Pt4 exhibited differential expression of MYC Targets including *C-MYC*, Pt6 exhibited regulation of Interferon Response pathways implying sensitivity to DNA damage. Notably, Pt6 tumor with an APOBEC mutator phenotype (Pt4 and Pt5 have HRD phenotype) exhibited recruitment and repression of Interferon Gamma pathways and G2M Checkpoints. Furthermore, activation of the Interferon Alpha Signaling pathways could contribute to apoptosis and cellular senescence but are also attributed to increased migration and drug resistance depending on the interferon-stimulated genes transcribed [43, 44].

### Pseudotime analysis identifies intermediate differentiation cell states in reversible drug resistance

We next asked whether states of transcriptional resistance induction and reversion could be identified ab initio. Differential expression analysis tends to summarize a population by a mean value and is therefore less effective in capturing subtle subpopulations that include distinct intermediate differentiation states, while the pseudotime analysis allows to place cells into the global landscape and emphasize the transition between cell states based on global transcriptional changes. To address this, we conducted a pseudotime trajectory analysis (Fig. 6, Additional file 1: Figures S13 and S14) over actual time series samples.

Firstly, we applied Slingshot [45], an efficient computational method that can robustly infer multiple branching cell lineages and pseudotime from single-cell gene expression data. For each patient Pt4-6, we used all untreated, treated, and drug holiday scRNA-seq cells (see Table 1), and applied Slingshot to obtain inferred lineages over pseudotime. As a result, each cell can be associated with one or more lineages and be assigned a pseudotime value (Fig. 6, Additional file 1: Figures S13 and S14, Methods).

In Pt4, the pseudotime inference output embedded in UMAP projection reveals two main distinct lineages that correspond to the untreated cell population (lineage L1), and the treated/holiday cell population, confirming the expected differentiation between the treated versus untreated cells. However, the treated/holiday population was further sub-divided into two sub-lineages: L3 associated with continuous drug exposure, and L2 associated with drug holiday (Fig. 6a). Sub-lineages L2 and L3 share the same starting clusters at the beginning of treatment where cells have a similar effect to the drug, then at the late time points, with enough drug exposure, cells are divided into resistant cell subpopulations (resistant clone A, fitness coefficient median = 1.048, sd = 0.019 Additional file 1: Figure S13a, b) along lineage L3, and drug holiday reversibility cells population L2 (drug holiday clone B, fitness coefficient median = 1.023, sd = 0.015 Additional file 1: Figure S13a, b) along lineage L2 of Pt4. This result is consistent with the results at the genomic level from our previous study [26] (Fig. 2a, c). The UMAP projection (Fig. 6a) further suggests that the drug holiday cells in L2 are distinct from the untreated cells, suggesting that the cells exist in a different state than fully resistant or fully untreated. A similar trend was observed for Pt6 (Additional file 1: Figure S14), where Pt6 lineage L2 appears to represent a distinct treatment holiday state, distinct from the variation between untreated and treated states. As with Pt4, some holiday cells fall back towards the untreated, or remain close to the treated state. In contrast, Pt5 exhibited no distinct holiday state. Here two main lineages that correspond to untreated cells (L1) and treatment/holiday cells, further differentiated into three treatment sub-lineages that are differentiated by cells at the late drug time points (L2, L3) and L4 containing mostly one round of treated cells. Importantly, the cell landscape at the end points of lineage L2 contains drug-resistant cells (resistant clone A, fitness coefficient median = 1.047, sd = 0.018, Additional file 1: Figure S13c, d). Moreover, the end points of lineage L3 contains drug holiday cells in the similar landscape as untreated cells (Additional file 1: Figure S13c, d), suggesting that the majority of drug holiday cells revert back to states that are close to the ground state of untreated cells. The mapping between pseudotime lineages and clonal landscape was highlighted in Additional file 1: Figure S13c, d, e where resistant clones or drug holiday clones are present near the end points of treated, and holiday lineages, and early, shrinking cell clones are detected near the beginning of each lineage.

We next asked which biological functions are represented in the different lineage states of treatment and treatment withdrawal and their relationship to known states of drug resistance. We used tradeSeq [46] to perform between-lineage statistical tests in order to extract differentially expressed genes across lineages in Pt4-6. Moreover, tradeSeq admits identification of differentially expressed genes between the end points of each pair of lineages where there was a strong drug effect (see Methods).

The identified tradeSeq significant genes (Wald statistics tests assessing differentially expressed genes along multiple lineages, and between early, or end points of lineages, *p*-value < 0.05, see Methods) were grouped into gene modules (Fig. 6c, d, Additional file 1: Figure S14, Additional file 2: Table S10). The computed gene modules identified three main patterns of gene regulation with drug effects: (i) genes that are induced relative to untreated state and travel similarly with treatment and holiday (Pt4 modules M1, M2 31.9%; Pt5 M2, 14.3%; Pt6 M4 19.8%); (ii) genes repressed relative to the untreated state by treatment (Pt4 module M4, 29.7%; Pt5 M3, M5 33.2%; Pt6 M2 8.6%), and (iii) strikingly, holiday state genes whose expression is in neither treated nor untreated state, indicating that treatment withdrawal can be associated with novel intermediate states and not simply a reversion to untreated ground state or a fixation to the treated state (Pt4 modules M5, M6 18.4%; Pt6 M3, M5 58.1%). Precisely, genes in modules Pt4 M3, M5 (28.1%) are induced along drug treatment, but are repressed at drug holiday. In contrast, genes in module Pt4 M6 (10.3%) are repressed along drug treatment, but upregulated at drug holiday time points (Fig. 6c, e). The dominant biological processes [47, 48] in the distinct holiday states was EMT related for Pt4 (M4, M5, M6) and a mixture of interferon response and EMT for Pt6 (Fig. 6c, d, Additional file 1: Figure S14, Additional file 2: Table S10, M3, M5). In contrast, common functions between shared treatment and holiday states were dominated by cell cycle functions such as G2/M checkpoint, mitotic spindle, E2F targets, consistent with the effect of DNA damaging agents in dividing cells. These results match the pathway analysis results in the previous section (Fig. 5). With two separate analyses, we see consistent pathway mechanisms that are activated under drug treatment and drug holiday conditions.

Further, we investigated the potential promoter chromatin states of the identified modules in Pt4-6 using the ChIP-seq data for H3K27me3 and H3K4me3 from untreated breast cancer cell line MDA-MB-468 [49]. For Pt4, we found gene modules M1, M2, and M4 to have active promoter chromatin status with significant enrichment of H3K4me3 (*P* < 0.05, ANOVA) and significant depletion of H3K27me3 (*P* < 0.05, ANOVA) compared to repressed (low expressed) genes. While two modules, M3 and M6, had bivalent status having H3K4me3 enrichment (*P* < 0.05, ANOVA), but having similar high levels of H3K27me3 as found at repressed genes (*P* > 0.05, ANOVA test), and lastly M5 gene module was found to have repressed state with the same chromatin state for H3K27me3 and H3K4me3 as found at repressed genes (*P* > 0.05, ANOVA) (Fig. 6c, right column, see Methods). In contrast with Pt4, none of the five modules in Pt5 and Pt6 were found to have active promoter chromatin status, but instead had either bivalent/repressed status in Pt5 (Fig. 6d, right column, see Methods) or only bivalent status in Pt6 (Additional file 1: Figure S14). Unique holiday module genes were associated only with bivalent or repressed marks across Pt4,6.

In addition, we characterized each tradeSeq significant regulated gene as in-cis or in-trans depending on the inferred cell clonal labels and the copy number change between clones in each lineage (Fig. 6c, d, Additional file 1: Figure S14c). We observed that the number of in-cis genes in treated lineages are higher than in untreated lineage for Pt4, Pt5 (Additional file 1: Figure S13f), demonstrating that genes are strongly dynamic under drug treatment lineages compared to untreated lineages (Pt4: untreated lineage L1: 58% in-cis genes compared to 66% in treated lineages L2, L3. Pt5: untreated lineage L1: 56% in-cis genes compared to 61% in treated lineages L2, L3, L4). In Pt6, in-cis genes contributions are similar (9%), and lesser than in-trans genes (91%) across lineages. This result is consistent with earlier analysis with the observation that most regulated gene types in Pt6 are in-trans.

In summary, the pseudotime analysis allows us to strengthen the results from the earlier analyses. The untreated, treated, and drug holiday identified lineages match with the observations in the earlier analysis of drug response and clonal prevalence. Moreover, the regulated gene modules as a result of the pseudotime analysis enriched similar main pathways as in the earlier analyses.

## Discussion

Copy number mutations alter gene dosage potential over hundreds of genes in breast cancer, thus single mutational events have the potential to impact transcription of many genes [10, 11]. Here we show how the relationship between gene dosage in subclones of breast cancer and non-genomic transcriptional plasticity impacts transcription in the setting of platinum treatment and platinum withdrawal in multi-clonal triple negative breast cancers. We contrasted three TNBC patient tumors characterized by long interval, multi-year serial passaging [26] and different genomic instability backgrounds representative of the spectrum of TNBC patients [10, 13, 14, 50, 51], where serial tumor sampling of platinum treatment and platinum withdrawal were available. To separate genotypic and non-genotypic transcriptional dynamics we generated scRNA-seq from the serially passaged tumors, assigning cells to clones with a probabilistic model informed by prior single-cell genome sequencing determination of CNA clone genotypes. As anticipated, the proportion of differentially expressed transcripts potentially impacted by gene dosage is a reflection of the fraction of genome altered by CNAs and ranges from 5 to 50%. Whole genome duplication represented only a minority of tumor cells in Pt4-6 (mean 2.86%, sd 1.92%), thus most of the in-cis variation arises from chromosomal level CNA.

A striking result is that different clones even within a tumor may exhibit very distinct modes of transcriptional landscape fixation. The dynamics of gene expression on exposure to platinum results in a majority increase in transcripts, dominated by non-copy number associated regions of the genome (in-trans). In-cis region transcripts were correlated with gene dosage. The pattern of transcription on withdrawal appears to partially reflect the degree of clonal selection and fixation in tumor cell population, consistent with previous studies where it has been shown that phenotypic changes are not necessarily associated with genomic changes, but rather transcriptional plasticity plays a major role [16]. The most extreme example in our data is Pt4, which exhibits very little transcriptional variation upon withdrawal of platinum by later passages when the entire population is composed of a single drug-resistant clone. Pt5 shows an intermediate effect in clonal selection and at least one clone that exemplifies clonal fixation, whereas Pt6 that has the least genome altered did not exhibit clonally fixed states of transcription. These results emphasize the internal heterogeneity of clones with respect to the contributions of genomic and non-genomic transcriptional plasticity. Transcriptional plasticity implies short-term responses to drug treatment which may be reversible. We observed that a large part of the transcriptional response is not fixed. On the other hand*,* gene dosage mutations are unlikely to revert to ground state, especially where deletions occur. We showed that in-cis genes (whose expression may be influenced by gene dosage) are less prone to variation, depending on the nature of the change. Complete deletion is irreversible, gene amplification could progress in either direction (more gains or loss after gain). Once a resistant (genomic) clone having in-cis genes becomes dominant and fixed in the tumor population, the possibility of clonal competition with less fit precursors no longer exists. Potential clinical significance lies in the degree of reversibility of each mechanism. We previously showed that clonal dominance of resistance (i.e., where gene dosage mutations may carry some of the fitness) results in irreversible states. On the other hand, where clonal dominance does not occur, reversal of clone determined fitness may occur during treatment holiday and patients’ tumors could remain sensitive to re-challenge.

The anti-correlated transcripts we identified could arise through multiple mechanisms, for example, some genes gaining copy gains could get downregulated due to copy gains of other repressor transcription factors or imbalances in the gene regulatory networks that regulate its expression; genes that maintain a fixed level of gene product due to network interactions; some gene dosage alterations resulting in altered epigenetic marks leading to the counter-regulation, etc.

A further question relates to the basis of memory in tumor tissues exposed to drugs. Here we observed that while the majority of drug-induced transcription can revert, reversion is not always associated with return to the untreated state but rather new states that represent a potential reservoir of intrinsically resistant cells. Perhaps this plasticity results in priming or keeping the memory of exposure [52] through formation of new states that could define the fate of those cells. Bivalent promoters are known for repressing the developmental genes in embryonic stem cells which gain expression during the differentiation for instructing lineage specification [53] via epigenetic mechanisms [54]. Interestingly, all the five gene modules achieved from pseudotime analysis in Pt6 were enriched in bivalent promoters (Additional file 1: Figure S14c), while comparatively only two and three modules were enriched in bivalent promoters for Pt4 and Pt5 respectively (Fig. 6c, d). Therefore, we believe that the activation of the developmental/differentiation genes in the bivalent promoters enriched modules in Pt6 during its response to drug treatment instructed lineage specification to new state; hence, Pt6 holiday passages did not revert back to drug-naïve transcriptional state when compared to the Pt4 and Pt5.

Pathway analysis shows that convergence on altered EMT state, a known phenotypic background for drug resistance, is one common factor in the reversion trajectories, additionally common interferon pathways and cytokine signaling represent a convergence point. We observed that the four common pathways identified in all three serially passaged and cisplatin-treated deep xenografts are also present in other cancers subjected to cisplatin treatment. Transcriptomic profiles of cisplatin-treated small cell lung carcinomas [55] revealed similar pathways (hypoxia, EMT, TNFA-signaling, Interferon signaling) as those identified through time series sampling. A recent single-cell study of different chemotherapeutic agents [56] also suggested pathway convergence, where samples were collected pre-treatment, mid-treatment, and post-treatment, involving a combination of drugs including doxorubicin, Taxol, and VEGF inhibitors. Despite differences in the drug combinations and sampling methods, we observed common pathways shared with our findings.

Our study also has certain limitations. It should be noted that PDX models, while capturing many aspects of polyclonal tumor evolution and intrinsic tumor cell phenotypes, do not mirror the effects of the human immune system nor replicate precisely the tissue-level pharmacology of drug exposure. However, the limitations of human tissue biopsy for patients under treatment also preclude the serial analysis needed to measure clonal fitness accurately. The patient tumors studied here represent only a subset of TNBC tumor subtypes, albeit sampled intensively over multi-year intervals. More extensive multi-time point sampling of TNBC would be desirable to map the relationships between fitness, gene dosage, and transcriptional reversion in a fully quantitative manner. It is likely that recent discoveries of chromosomal instability subtypes of TNBC characterized by foldback inversion patterns (FBI [50]), distinct from homologous recombination deficient TNBC, imply additional mechanisms of in-cis and in-trans drug resistance. FBI tumors are intrinsically platinum resistant and tend to exhibit higher genome duplication rates.

## Conclusions

Taken together, our results emphasize that both CNA-associated and CNA-independent mechanisms may encode transcriptional states associated with platinum resistance and emphasize the internal heterogeneity within tumors with respect to each mechanism. The contribution of each mechanism may have implications for therapeutic strategies. Strong genomic clonal fixation with a clonal sweep implies irreversibility, whereas tumors with heterogeneous clones may amplify non-genomic transcriptional states, with implications for re-treatment or re-sensitization approaches. Future studies of transcriptional plasticity should account for both aspects to achieve a realistic understanding of polyclonal tumor responses to therapy.

## Methods

### Establishment and serial passaging of patient-derived xenografts

#### Serial transplantation of patient-derived xenografts (PDX)

Xenograft-bearing mice were euthanized when the size of the tumors approached 1000 mm^3^ in volume (combining together the sizes of individual tumors when more than one was present). The tumor material was excised aseptically, then processed as previously described [26, 57]. Briefly, the tumor was harvested and minced finely with scalpels, then mechanically disaggregated for 1 min using the Stomacher 80 Biomaster (Seward Limited, Worthing, UK) in 1–2 mL cold DMEM-F12 medium. Aliquots from the resulting suspension of cells and fragments were used for xenotransplants in the next generation of mice.

#### Histopathology of PDX tumors

The hormone receptor status of all tumor samples were determined by immunohistochemistry. Two separate tissue microarrays (TMAs) were prepared using duplicate 1-mm cores extracted from formalin-fixed paraffin-embedded (FFPE) blocks, containing materials from patient derived xenografts (Pt4, Pt5, Pt6) as described in [26, 36] (Additional file 1: Figure S1).

#### TNBC PDX timeseries treatment with cisplatin

Triple negative breast cancer patient-derived xenografts previously established as described [26, 36, 57] were serially passaged as described [26, 57]. Serially transplanted material represented approximately 0.1–0.3% of the original tumor volume. Immunodeficient NOD/Rag1 − / − Il2rγ − / − (NRG) mice of the same age and genotype as above were used for transplantation treatment experiments. Drug treatment with cisplatin (Accord DIN: 02355183) was started when the tumor size reached approximately 300 to 400 mm^3^. Cisplatin was administered intraperitoneally (IP) at 2 mg kg^−1^ every third day for 8 doses maximum (Q3Dx8). Low-dose cisplatin pulse was selected to achieve the experimental aims of tumor resistance at the time of tumor collection. Residual tumors after treatment were re-transplanted in a new cohort of eight mice. Also, in parallel for the treatment/treatment holiday study group, half of the mice were treated with cisplatin when tumors exhibited approximately 50% shrinkage, the residual tumor was then harvested and re-transplanted for the next passage in the group of eight mice. Again, half of the mice at the second cycle of treatment were kept untreated while the other half were exposed again to cisplatin following the same dosing strategy. Four cycles of cisplatin treatment were generated, with a parallel drug holiday group at each passage.

We used the label Rx to denote treated samples. RxH denotes samples treated over one or more passages but kept on drug holiday (drug withdrawal) at that specific (last) passage. UnRx denotes samples that were never exposed to drug treatment.

In the re-transplant, all implanted mice were left without drug treatment for a period of 4–5 weeks until visible tumors formed and then half the mice were re-treated (Rx arm) and half left to grow further to endpoint—the latter harvested as the holiday interval (RxH) tumors, this is in keeping with the way humans are treated. Although we did not sample gene expression immediately before treatment started (this would have been destructive to the tumors), the observed responses of clones in the treated mice reflects a drug-free period before treatment started, mirroring the way chemotherapy is delivered to patients. The holiday population (RxH) was without the drug for the same duration as the parallel mice treated with cisplatin for one cycle. RxH was subjected to one, two, or three cycles of cisplatin but entered a drug-free or holiday state in the corresponding passage, where they are compared with tumors continuously treated with cisplatin and those entirely treatment-naive. The RxH are parallel transplants that never again receive drugs and are allowed to grow, to establish the full reversion status of the transcriptome after the prior treatment.

For patient naming, Pt1, Pt2, Pt3 in this study correspond to the breast cancer patient-derived xenograft SA501, SA530, SA604 [50], and Pt4, Pt5, Pt6 in this study correspond to SA609, SA535, and SA1035 respectively in our previous study [26], see also Table 1 and Additional file 2: Table S1. The growth curves that contain the details of tumor volume (mm^3^) and days from palpable tumor to collection are noted in Extended Data Fig. 3 of our previous study [26].

#### Replicate lineages of treatment, holiday, and no treatment

Replication included (i) biological replicate transplants per time point; (ii) lineage replicates, where transplants were serially transplanted in parallel; (iii) re-mixing and re-transplant of tumor populations of different putative fitness at different population fractions, with a re-run of serial treatment or passaging, to revalidate the clone dynamics in each situation. These details are reported in our previous paper [26]. We conducted parallel analyses of untreated lineage and counter drug holiday samples for that time point.

#### Single-cell whole genome sequencing and library construction with DLP+

Single-cell whole genome sequencing and library construction with (Direct Library Preparation) DLP + were done as described in [26, 58]. Briefly, single-cell suspensions from the tumors were prepared by enzymatic digestion with collagenase/hyaluronidase (Stem Cell Technologies, 07912) enzyme mix in serum-free Dulbecco’s modified Eagle’s medium (DMEM) at 37 °C with intermittent gentle trituration with a wide bore pipette tip. Cells were stained with LIVE/DEAD Fixable Red Dead Cell Stains (ThermoFisher) and using a cellenONE (Cellenion), single cells dispensed into each well on a nanowell chip containing two unique dual indices. DLP + sequence analysis, copy number determination and quality control filtering were done as in [26].

#### Processing of patient-derived xenografts for single-cell RNA sequencing

Viably frozen vial of the PDX tumors was thawed and after washing out the freezing media, the tumor clumps and fragments were incubated with digestion enzymes as previously described [26, 58]. The cells were resuspended in 0.04% bovine fetal serum (BSA in PBS). Dead cells were removed using the Miltenyi MACS Dead Cell Removal kit, and cells were processed as described in [59]. For library construction, the samples at the same time point were sequenced on the same chips to avoid processing artifacts.

### Data filtering and analysis

#### Copy number estimation, phylogenetic tree inference, clone determination, and clonal abundance measurements in DLP+

We applied the DLP + single-cell analysis pipeline that was developed in the previous study [26, 58] for single-cell DNA sequencing datasets. Firstly, the read data from DLP + single-cell sequencing was preprocessed using HMMcopy package version 1.32.0 to provide a copy number profile for each cell with 500 kb fixed-length non-overlapping genomic regions [60]. Secondly, we reconstructed phylogenetic trees by applying sitka [27], an efficient Bayesian tree inference method to copy number data. Cells are placed at the terminal leaf nodes in the phylogenetic tree, and cells with high similarity in copy number profiles are placed in the neighborhood area of the same branch in the tree. Thirdly, based on the output of the phylogenetic tree, clonal populations are identified. The connected components of cells in the neighborhood area on the phylogenetic tree were identified and cells were divided into clones based on the degree of homogeneity in copy number profiles. Fourthly, the fitness coefficient which denotes a growth potential rate of a given clone along the drug evolution was previously quantified using the fitClone tool [26] for Pt4-6 and used as such in this study.

To simplify the phylogenetic tree for visualization in this study, sitka trees were collapsed and cells in the same clone were represented by one supercell—one circle with the given clone label. The collapsed tree allows us to observe the order of clones, and the clades in the tree. The output of the collapsed trees are shown in Fig. 2a. The clones with the fittest coefficients across treatment as calculated by fitClone are annotated with *Rx, and across untreated lines are annotated with *UnRx. The phylogenetic trees of the three drug-treated patients Pt4, Pt5, Pt6 were generated in our previous study [26]. We applied the same methods to reconstruct the trees for three untreated patients Pt1, Pt2, Pt3 in this manuscript (Fig. 2a).

#### Quality control for the scRNA-seq data

Firstly, count matrices were generated using CellRanger version 3.0.2 (V3 chemistry), see column “Nb_cells_initial” in Additional file 2: Table S1. Secondly, all mouse cells that were mixed with human cells were eliminated. Cells were aligned using both mouse and human reference gene sets. A cell is classified as a mouse cell if the total number of counts aligned to mouse reference of the 10 × sample was greater than the total number of counts aligned to the human reference (column “Nb_cells_no_mouse”). Thirdly, cells were considered to have passed a quality control filter (QC-filter) and retained for subsequent analysis if they met the following criterion: (i) minimum of 1000 detected genes, (ii) low mitochondrial contamination with less than 20% of UMI counts mapping to mitochondrial genes, (iii) less than 60% of UMI counts mapping to ribosomal genes, and (iv) the total counts (UMIs) per cell was at most 3 median absolute deviations lower than the overall median counts. Cells with lower quality than the above criteria were filtered using the calculateQCMetrics and isOutlier functions in the scater package [61] (column “Nb_cells_QC_filter”). Fourthly, we eliminated doublets using package scrublet [62] (column “Nb_cells_for_analysis_no_doublets” in Additional file 2: Table S1).

#### Clonealign

Clonealign version 1.99.2 [28] was used to align scRNA-seq cells from each specific xenograft sample to the DLP + clones obtained from the same xenograft sample. Clonealign assumes that, for most genes, the non-diploid copy number is positively correlated with the gene expression level. Manual tuning was necessary for some of the parameters due to the high degree of heterogeneity in the noise of our samples (see Additional file 2: Table S3), as follows:

1. Copy number purity CN_purity threshold for a gene in a clone, defined as the percentage of cells that have the modal copy number for that gene and clone, was set to 0.6 for most samples. For example, if the copy number mode of a gene in a clone is 4, then the gene is included in the clonealign model only if at least 60% of the cells in that clone have copy number 4 for that gene. This ensures that only the highest confidence data is included in the analysis. However, for some samples from Pt4 and Pt6, no or very few genes pass this threshold; therefore, we used lower thresholds for those samples.
2. min_counts_per_cell of 25–100 was used to remove the cells that have lower total counts in the selected genes than this threshold. This parameter is intended to ensure that only high gene count cells are included in each clone transcriptome.
3. data_init_mu was set to TRUE if the mu parameters need to be initialized using the data, or FALSE otherwise.

Other parameters that had the same values for all libraries include *n* repeats = 3, mc samples = 1, learning rate = 0.07, max iter = 500, and saturation threshold = 6.

#### Differential expression analysis

Differential expression quantifiers including log2 fold change and false discovery rate (FDR) were computed using the R 3.6.0 Bioconductor package edgeR 3.26.0 [30] that implements scRNA-seq differential expression analysis methodology based on the Negative Binomial distribution. First, we applied scran normalization to remove library size effects and use the obtained size factors for the next step. Then, we called the estimateDisp function to estimate the dispersion by fitting a generalized linear model that accounts for all systematic sources of variation. Next, we used the edgeR functions glmQLFit and glmQLFTest to perform a quasi-likelihood dispersion estimation and hypothesis testing that assigns FDR values to each gene. In the track scRNAseq plots in Fig. 3 and Additional file 1: Figure S5, a positive log 2 fold change value for Clone X relative to Clone Y signifies that the gene is significantly more upregulated (at a given FDR threshold) in X than in Y while taking into consideration all the expression values for all the genes in both clones. Similarly, a gene with negative log twofold change is significantly more downregulated in X than in Y.

#### Normalization method

We applied SCTransform [63] to normalize the gene expression matrix and regress the difference in sequencing depths between samples. The normalized matrix will be used for pseudotime analysis and UMAP visualization. First, scRNA-seq data were preprocessed. Low expression genes that have zero UMI counts in more than 97.5% of the cells were considered as unreliable genes and were excluded from our analysis. Then, from the list of filtered genes, the mitochondrial confounding genes were removed. SCTransform model genes using Pearson residuals from regularized negative binomial regression. The generalized linear model from SCTransform uses a covariate value that accounts for sequencing depth. First we removed genes with low expression and confounding genes as described above. Then we used the SCTransform function within Seurat version 4.0 to normalize data, resulting in a SCTransform log-normalized expression matrix.

#### Dimensionality reduction computation

To visualize the dimensionality reduction map, we applied a basic processing pipeline in Seurat version 4.0 [64] to compute the UMAP features map. First, the gene expression matrix was normalized as described above using SCTransform. The 30 principal component vectors (PCA) are computed from a log normalized gene expression matrix. Then UMAP vectors were computed based on 30 PCA vectors using the uwot package that is included within the Seurat version 4.0 platform.

#### CNA genotype-dependent in-cis and independent in-trans gene detection

To classify genes based on their copy number (CNA) genotype dependent/independent, we implement the following steps: (i) summarize the copy number profile of each clone; (ii) assign copy number profile to genes in each clone at the overlapping of gene genomic regions and bin genomic regions; (iii) classify genes into different gene types based on copy number alterations.

(i) Computing the median copy number profile of each clone: first we grouped cells based on cell clonal labels achieved from DLP + analysis procedure above, then for each clone, copy number values at each bin genomic region were summarized by the median copy number value of all cells at this bin genomic region in the given clone.
(ii) Assigning copy number profile to ensembl gene indexes: to find mapping between DLP + copy number results and scRNA-seq gene expression data, all overlaps between bin genomic regions and the genomic ranges of transcriptomic genes were taken into account. We used the R package org.Hs.eg.db version 3.8.2 [65] to find all genomic ranges (containing start, end, width) corresponding for a given ensembl gene index. Then findOverlaps function from R package iRangers [66] to find any overlaps between bin genomic regions and genomic ranges of each gene, resulting in the list of bin genomic regions and their corresponding gene indexes at the overlapping area.
(iii) Classifying genes into CNA genotype-dependent/independent in-cis, in-trans gene types based on copy number alterations: the input data for gene classification is differentially expressed genes in scRNA-seq data between two clones that were inferred using clonealign and copy number alteration between two clones from DLP + analysis at the overlapping genomic region. CNA genotype independent in-cis gene is a gene that exhibits the change in gene expression (DE gene) in scRNA-seq gene expression, and its copy number value. In contrast, CNA genotype-independent in-trans gene is a gene with its change in expression that only occurs at the transcriptomic level but does not exhibit any change in copy number values at the overlapping genomic regions. To compute in-cis genes, from DE genes that were obtained using edgeR differential expression analysis as described above, we tracked the assigned copy number profile for each gene, in case there is a change in copy number values, these genes were labeled as in-cis genes, otherwise were labeled as in-trans genes.

#### Gene classification: define six variation trends

Four main variation trends for in-cis genes based on the positive or negative directions of gene expressions and copy number profiles, and 2 variation trends for in-trans genes based on the direction of gene expressions were defined. Four main variations in-cis genes are: (i) *Gain Up*: *gain*, increase in median copy number values between two clones in DLP + results and upregulated with positive logFC value of gene expression in DE analysis between two inferred clones in scRNA-seq. (ii) *Loss Down*: *loss*, decrease in copy number values between two clones in DLP + results and downregulated in gene expression with negative logFC value of gene expression in DE analysis between two inferred clones in scRNA-seq. (iii) *Gain Down*: *gain*, increase in median copy number values and downregulated in gene expression. (iv) *Loss Up*: *decrease* in copy number values and upregulated in gene expression. Moreover, based on the directions, we classified in-cis genes into the copy-number correlated in-cis gene in case the gene shows the same direction in both transcript and genomic levels: Gain Up, Loss Down, or copy-number anti-correlated in-cis genes in case opposite direction: Gain Down, Loss Up. Two variation trends that can occur in-trans genes are (i) In-trans Up: upregulated gene in scRNA-seq gene expression (U) and (ii) In-trans Down: downregulated gene in scRNA-seq gene expression (D) (Fig. 3).

#### Mapping reference genes to our DE genes results

Next, we investigated whether the significant genes obtained from our analysis belong to the list of cancer functional genes. To address this, first, we scanned two databases and denoted as custom reference gene sets, including ADAM PanCancer core cancer fitness [34], and the genes involved in cisplatin resistance were curated from recent literature (Additional file 2: Table S6). Then we computed the fraction of in-cis, in-trans DE genes from our results that belong to two reference gene sets (Fig. 3, Additional file 1: Figure S6). Moreover, to examine whether the differentially expressed genes obtained from our analysis enriched any custom reference gene set of core cancer fitness or cisplatin-related genes, we adopted the pathway enrichment analysis applying R package fgsea version 1.22.0 [67]. Similar to pathway analysis using well-known gene sets, here we used two custom gene sets as input, and logFC values of significant genes from DE analysis results as input. The significant pathway results with *p*-adjusted value < 0.05 are shown in Fig. 3d.

#### Track plots

Top plot used scatter plot in R package ggplot2 version 3.3.3 [68], each dot denote a DE gene, *y*-axis: log2 fold change value of each gene in DE analysis result, *x*-axis: the genomic position of gene, calculated by extracting start, end, and chromosome genomic position of a given gene using R package annotables version 0.1.91 (https://github.com/stephenturner/annotables). We divide genes into subplots based on the chromosome positions (chr 1:22 and chr X) Bottom: heatmap plots of median copy number profile for pair of clones from output of DLP + copy number analysis.

#### Gene dynamic level quantification

Two types of dynamic genes were identified as follows. Firstly, to identify genes that were induced or repressed following cisplatin treatment (Fig. 4a–d), we selected the genes that were differentially expressed between Rx and UnRx (FDR < 0.01, |log2 fold change|> 0.5) and non-differentially expressed between Rx and RxH (FDR > 0.1), to obtain a set of genes with significant change in expression after treatment, but stable after drug withdrawal. Some of these genes had increased expression after treatment (induced), and some had decreased expression (repressed).

Secondly, to identify the genes that displayed dynamic changes following drug withdrawal, we computed the differentially expressed genes between Rx and RxH (FDR < 0.01, |log2 fold change|> 0.5), and intersected them with all the UnRx genes in order to measure the direction of change. While most genes reverted back towards the untreated state, some genes changed away from the untreated state.

#### Pathway analysis

Pathway Enrichment Networks were computed from differentially expressed genes (FDR < 0.01) ranked by log2 fold change. A normalized enrichment score (NES) was calculated from a ranked gene set enrichment analysis (GSEA) [38] using the gseapy Python package version 0.10.3, performed on each subset of differentially expressed genes using the hallmark gene set collection from MSigDB [39]. Significantly enriched pathways (adjusted *p*-value < 0.05) and pathway-specific differentially expressed genes were included in the network enrichment (Fig. 5).

#### Trajectory analysis

Slingshot [45] is an efficient method which can robustly infer cell trajectories and accurately capture pseudotime. In this study, Slingshot was used to infer pseudotime, and tradeseq [46] was used to extract differentially expressed genes across multiple lineages of pseudotime output. To prepare input data, we applied Seurat v4.0 [63] preprocessing steps. First, from SCTransform normalized gene expression matrix as described above, we selected 3000 most highly variable genes (HVG) using FindVariableFeatures function and “vst” method within Seurat. Following the common pseudotime analysis pipeline, we only take into account the top 3000 HVG genes in this study. The lowly expressed genes do not show the strong regulation and are excluded from analysis. The normalized expression matrix of HVG was used as input to extract 30 principal component (PCA) vectors. Thus, PCA vectors were used to do cell clustering using the Louvain algorithm, dividing cells into multiple clusters. Here we used the resolution 0.3 to get cell clusters at the fine grain level. Similar to the pseudotime analysis pipeline in general, we removed a small outlier cell cluster, and kept the main cell population.

To run slingshot, first one cell cluster was selected as the starting point of trajectories. In this study, the cluster with the most number of untreated cells from the earliest passage was selected as the starting point. Then, in order to estimate the global lineage structure by building a minimum spanning tree, we called the getLineages function from slingshot using as input 30 PCA vectors of all cells in each series. Based on the graph of main lineages, the smooth branching lineages and pseudotime values are estimated by fitting simultaneous principal curves using the getCurves function from Slingshot. The output is the pseudotime values that are assigned to each cell, and with multiple lineages, cells can be assigned to one or several lineages of development.

To visualize pseudotime results, we projected output into 2 UMAP vectors using embedCurves function (Fig. 6a, b, Additional file 1: Figures S13 and S14). Each lineage includes several clusters in the order of pseudotime. We added the arrows from one cluster to the next consecutive cluster in the given lineage to show the direction of development. The starting cluster is marked by a green circle and all intermediate clusters are marked by black circles and the ending cluster of each lineage is marked by a red circle.

We characterized each lineage based on the treatment conditions and inferred clonal cell labels from clonealign results. The prevalence plots of annotated labels are shown in Additional file 1: Figures S13 and S14.

Based on the output of trajectory inference, we applied tradeSeq in order to detect genes that displayed a strong differentiation between lineages. First, we ran tradeSeq fitGAM function to fit a negative binomial generalized additive model (NB-GAM) to the normalized count gene expression matrix, pseudotime value of each cell, and cell weights. Output of trajectory inference shows the substantial difference between untreated lineage and drug treatment lineages. In this study, we focus on “between lineages” comparison. A gene that is significant along a trajectory should satisfy two conditions: (i) gene expressions are varying along at least two or multiple lineages applying a statistical test patternTest function in tradeSeq; and (ii) gene expression be significantly different between endpoints—diffEndTest or early dividing points—earlyDETest of two lineages. We used a *p*-adjusted threshold value of 0.05 to select significant genes. Then, from the list of significant genes, we applied a threshold of 200 for patternTest significant level, and a threshold of 50 for diffEndTest, or earlyDETest to retrieve the most significant genes (Fig. 6c, d, Additional file 1: Figures S13 and S14).

In order to divide significant genes into multiple regulated gene modules, where each module displays a similar gene regulation pattern, we did gene clustering based on the Monocle3 genes clustering method [69]. From the list of significant genes, the cosine distance metric was applied to compute the distance between genes, and then extract 25 UMAP vectors. UMAP vectors were then used as input to the Leiden clustering method, with resolution 0.3 to extract gene module labels (Fig. 6c, d, Additional file 1: Figures S13 and S14).

Significant genes along trajectories are classified into in-cis, in-trans gene types in each lineage based on the list of inferred clonal labels and copy number profiles of each clone in DLP + . We assessed the inferred cell clone labels in each lineage. The copy number profile of these genes across clones is examined to see whether there is any change in copy number values. If there is a change, the gene is classified as in-cis gene, otherwise as in-trans gene in a given lineage (Fig. 6c, d, Additional file 1: Figures S13 and S14).

The regulation pattern of each gene module is summarized using the geom_smooth function in ggplot2. To define the significant hallmark gene set that are related to each gene module, we applied gprofilers statistical tests g:GOSt test using default parameter settings and hallmark reference gene set. The output of the gene sets along with a number of significant genes are displayed in Fig. 6e, f and Additional file 1: Figure S14d.

#### Chromatin status analysis

ChIP-seq data in untreated cancer cell line, MDA-MB-468, for H3K27me3 (GSM2258886 & GSM2258887), H3K4me3 (GSM2258892 & GSM2258893), and input control (GSM2258900 & GSM2258901) was retrieved from GEO [49]. ChIP-seq reads were mapped to the human genome (hg38) using Bowtie2 [70]. Using featureCounts (Subread package v 2.0.3) mapped reads were assigned to the promoters (2-kb window upstream of TSS) corresponding to the genes identified as part of the expression modules for each TNBC line in the pseudotime analysis. ChIP/input reads per million was calculated for each promoter in the respective modules to compare the enrichment of H3K27me3 and H3K4me3 with respect to a set of 500 repressed genes with non-zero lowest number of reads in Pt4 bulk data across time-series as a control for the genes with repressed chromatin status. Modules with significant enrichment of H3K4me3 (*P* < 0.05, ANOVA) and significant depletion of H3K27me3 (*P* < 0.05, ANOVA) compared to low expressing or repressed genes were labeled as having “Active Promoter” status. Meanwhile, modules having significant enrichment of H3K4me3 (*P* < 0.05, ANOVA test) but similar high levels of H3K27me3 as found at repressed genes (*P* > 0.05, ANOVA test) were labeled as having “Bivalent Promoter” status. Lastly, modules were labeled to have “Repressed Promoter” status if they have similar levels of H3K4me3 (low) and H3K27me3 (high) compared to repressed genes (*P* > 0.05, ANOVA).

### Supplementary Information

Additional file 1. Supplementary figures S1-S14, showing histology images, genomic and transcriptomic characteristics of TNBC PDX, copy number versus gene expression analysis, in-cis and in-trans genes, genes that are influenced by drug withdrawal, and pseudotime analysis.

Additional file 2. Supplementary tables S1-S10, showing a summary of the scRNA-seq data, clone fitness, clonealign parameters, differentially expressed genes, curated cisplatin resistance genes, drug withdrawal influence on genes, Hallmark gene set pathways and genes, gene modules from the pseudotime analysis.

Additional file 3. Review history.

## Data Availability

Uploaded Data URL: https://ega-archive.org/studies/EGAS00001007242 [29]. Github manuscript: https://github.com/molonc/drug_resistant_material/ [71] under Apache Licence v2.0. Zenodo archive, including processed files, code, results, pipelines and materials: 10.5281/zenodo.11397650 [72]. Third party Chip-Seq data was extracted from the GEO, see also [49]: https://www.ncbi.nlm.nih.gov/geo/query/acc.cgi?acc=GSM2258886 [73], https://www.ncbi.nlm.nih.gov/geo/query/acc.cgi?acc=GSM2258887 [74], https://www.ncbi.nlm.nih.gov/geo/query/acc.cgi?acc=GSM2258892 [75], https://www.ncbi.nlm.nih.gov/geo/query/acc.cgi?acc=GSM2258893 [76], https://www.ncbi.nlm.nih.gov/geo/query/acc.cgi?acc=GSM2258900 [77], https://www.ncbi.nlm.nih.gov/geo/query/acc.cgi?acc=GSM2258901 [78].
