# Supplementary material for: Single-cell decoding of drug induced transcriptomic reprogramming in triple negative breast cancers
Source: Genome Biol. 2024 Jul 18;25:191. doi: 10.1186/s13059-024-03318-3 (PMC11256464; doi:10.1186/s13059-024-03318-3)
Supplement: Supplementary file 1 — Additional file 1. Supplementary figures S1-S14, showing histology images, genomic and transcriptomic characteristics of TNBC PDX, copy number versus gene expression analysis, in-cis and in-trans genes, genes that are influenced by drug withdrawal, and pseudotime analysis. [file 13059_2024_3318_MOESM1_ESM.docx]

**Additional file 1: Supplementary figures**

**Single cell decoding of drug induced transcriptomic reprogramming in triple negative breast cancers**

Farhia Kabeer^1,2^*****, Hoa Tran^2^*****, Mirela Andronescu^1,2^*****, Gurdeep Singh^2^, Hakwoo Lee^2^, Sohrab Salehi^3,4^, Beixi Wang^2^, Justina Biele^2^, Jazmine Brimhall^2^, David Gee^2^, Viviana Cerda^2^, Ciara O'Flanagan^2^, Teresa Algara^2^, Takako Kono^2^, Sean Beatty^2^, Elena Zaikova^2^, Daniel Lai^1,2^, Eric Lee^2^, Richard Moore^5^, Andrew J. Mungall^5^, IMAXT Consortium^6^, Marc J. Williams^3^, Andrew Roth^2^, Kieran R. Campbell^7,8^, Sohrab P. Shah^3,4^, Samuel Aparicio^1,2^**^#^**.

^1^ Department of Pathology and Laboratory Medicine, University of British Columbia, Vancouver, BC, Canada

^2^ Department of Molecular Oncology, British Columbia Cancer Research Centre, Vancouver, BC, Canada

^3^ Computational Oncology, Department of Epidemiology and Biostatistics, Memorial Sloan Kettering Cancer Center, New York, NY, USA

^4^ Irving Institute for Cancer Dynamics, Columbia University, New York, NY, USA

^5^ Canada's Michael Smith Genome Sciences Centre, BC Cancer, Vancouver, BC, Canada

^6^ CRUK Grand Challenge IMAXT Team

^7^ Lunenfeld-Tanenbaum Research Institute, University of Toronto, Toronto, ON, Canada

^8^ Department of Molecular Genetics, University of Toronto, Toronto, ON, Canada

*****All these authors contributed equally

**^#^** Corresponding author


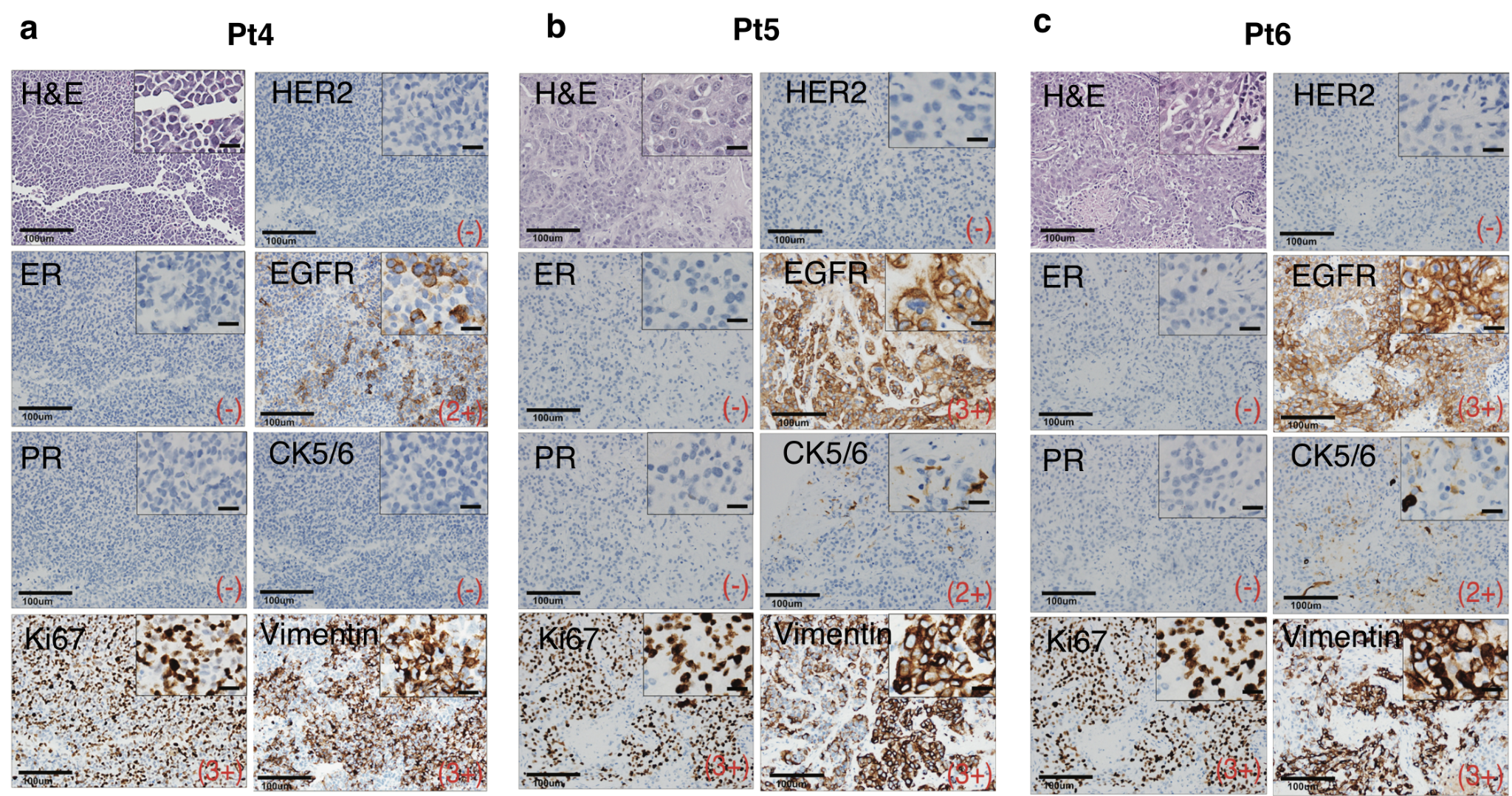


#### Figure S1: Histology images of TNBC patient’s tumor samples with H&E and immunohistochemistry staining

(a) Pt4 (b) Pt5 (c) Pt6. Scale bars 500um (main) and 100um (inset). Right bottom red values, pathologist determined, discretized score ranging from - (no staining) to 3+, based on staining intensity level of stained cells and number of cells that are expressed/ stained a marker compared to total cells in tissue.


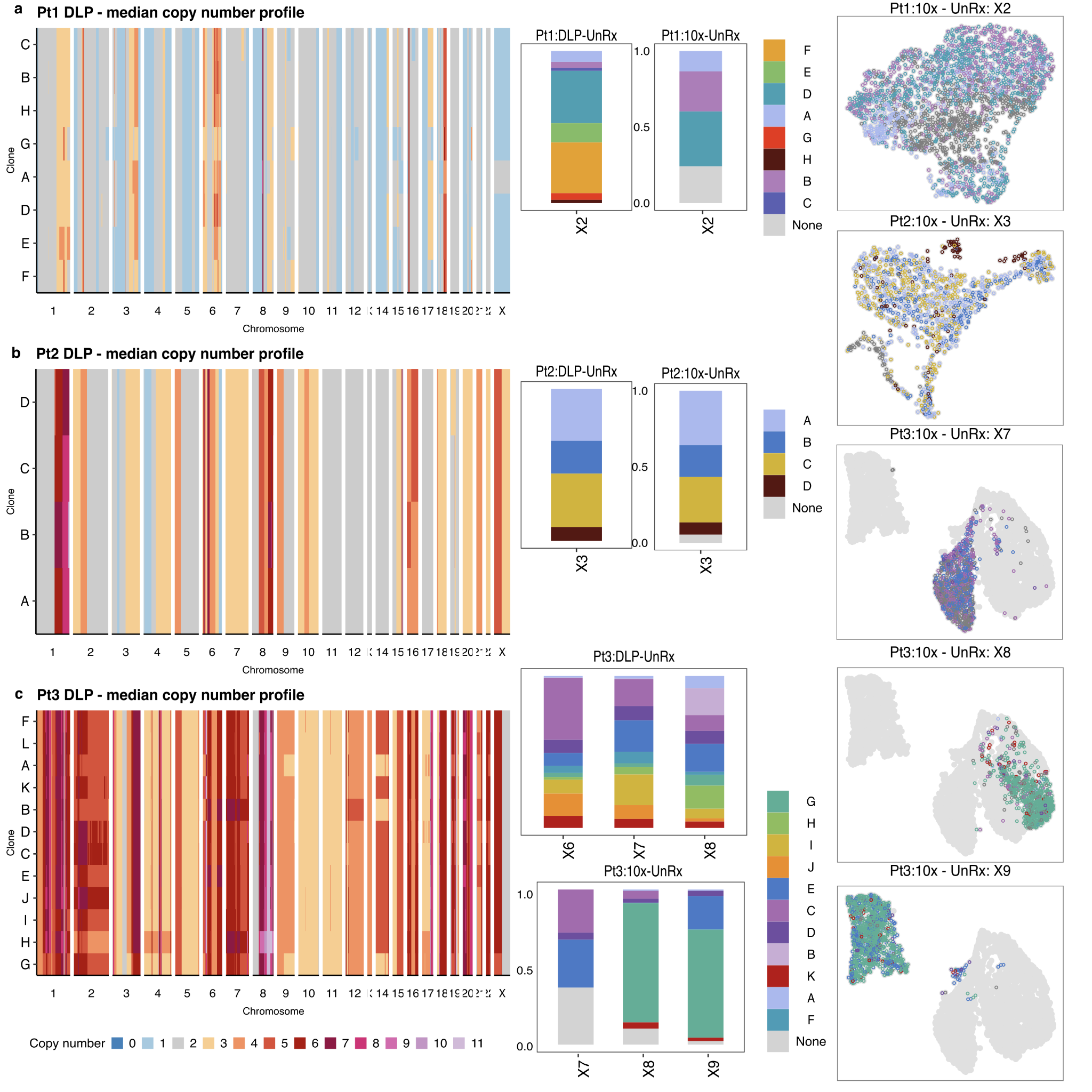


#### Figure S2: Genomic and transcriptomic characteristics of three untreated patients Pt1-3.

Segment copy number profiles of Pt1 (a), Pt2 (b), Pt3 (c) in DLP+ sitka phylogenetic tree results at left panels: heatmap with x-axis lists each chromosome, y-axis lists the sitka phylogenetic clones. Each entry represents the median copy number at the corresponding bin genomic position for each clone. Middle panels: Bar plots showing the clonal fractions in DLP+ and 10x data at each time point. “None” includes cells filtered out by clonealign and unassigned cells (see Methods, Table 1 and Additional file 2: Table S1). Right panels: cell populations UMAP plots in 10x scRNA-seq data, and each dot is one cell, colored by assigned clones.


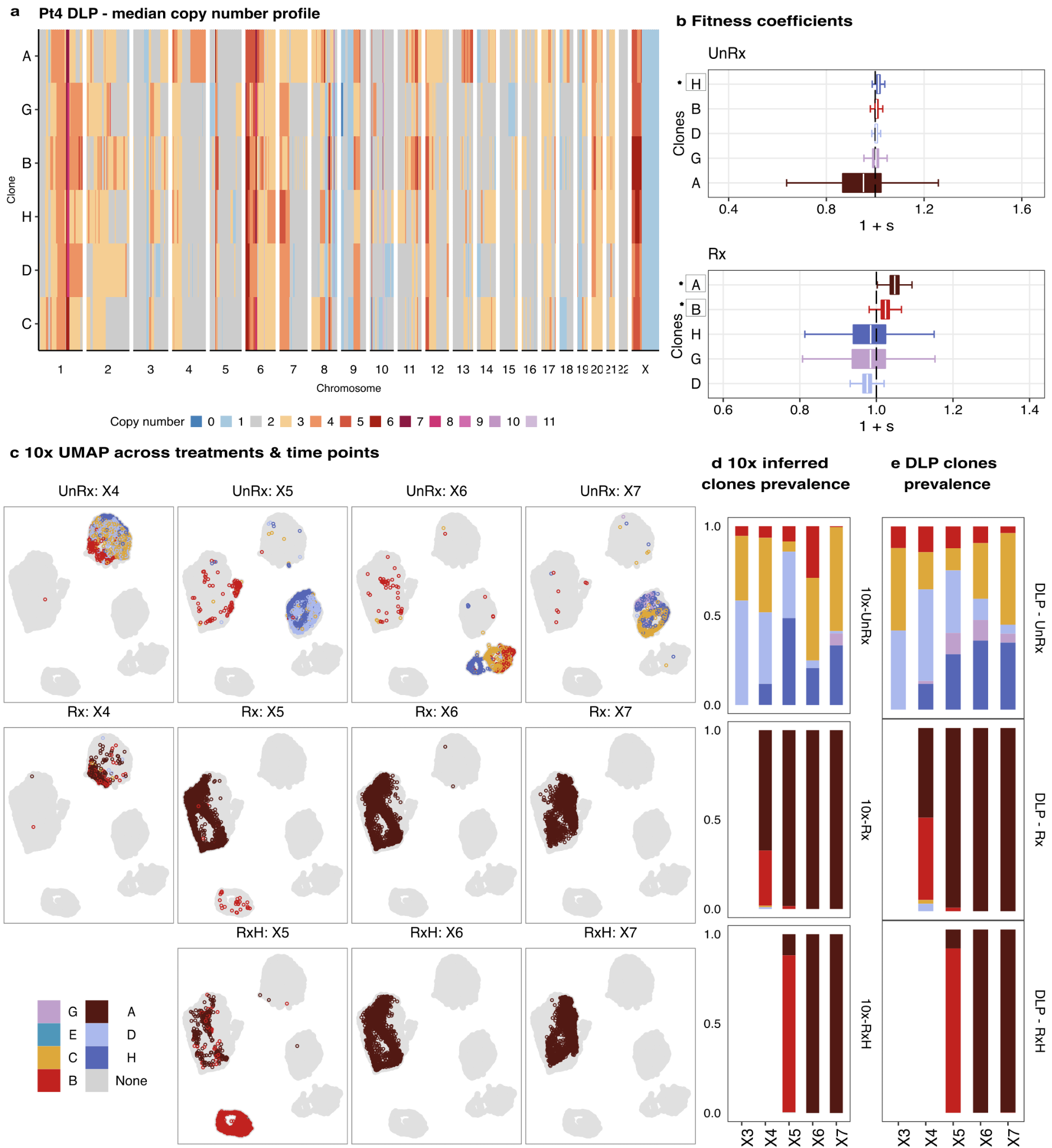


#### Figure S3: Genomic and transcriptomic characteristics of Pt4.

(a) Segment copy number profiles of Pt4 in DLP+ sitka phylogenetic tree results, heatmap with x-axis lists each chromosome, y-axis lists the sitka phylogenetic clones. Each entry represents the median copy number at the corresponding bin genomic position for each clone. (b) fitness coefficient of each clone in DLP+ from previous analysis . The stars * mark the clones with highest fitness coefficient in untreated, and treated cells. (c) UMAP of 10x scRNA-seq data denote the landscape of cells across drug treatments and untreated time points. Clonal prevalence of inferred clones in scRNAseq analysis (d), and of clones from sitka phylogenetic results in DLP+ analysis (e).


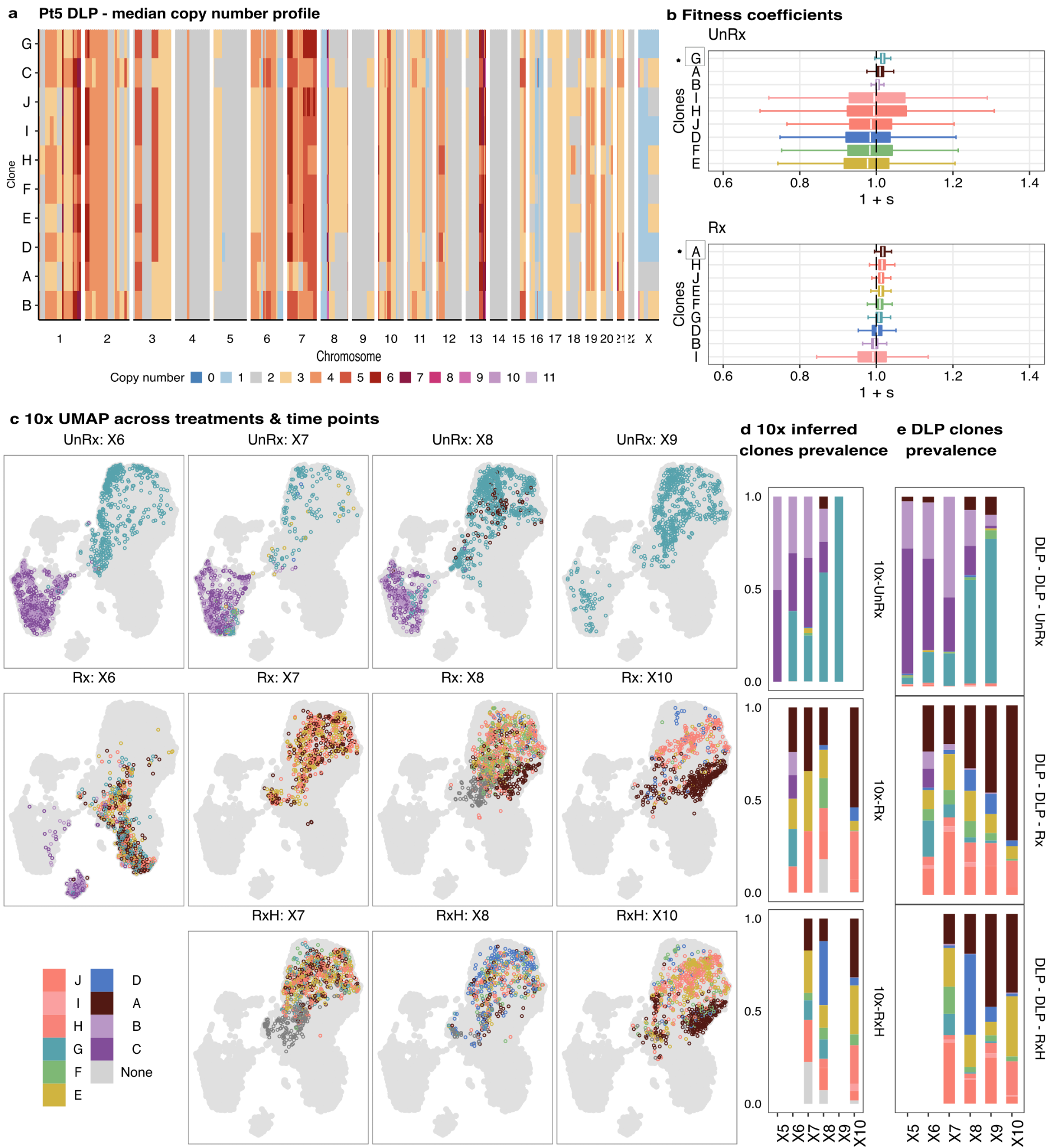


#### Figure S4: Genomic and transcriptomic characteristics of Pt5.

(a) Segment copy number profiles of Pt5 in DLP+ sitka phylogenetic tree results, heatmap with x-axis lists chromosome regions, y-axis lists the sitka phylogenetic clones. Each entry represents the median copy number at the corresponding bin genomic position for each clone. (b) fitness coefficient of each clone at genomic DLP+ data from previous analysis [[1]](https://paperpile.com/c/OLI0T7/uxVZ). The stars * mark the clones with highest fitness coefficient in untreated, and treated cells. (c) UMAP of 10x sc-RNAseq data denote the landscape of cells across drug treatments, drug holiday and untreated time points. (d) Clone prevalence of inferred clones in scRNA-seq analysis. “None” includes cells filtered out by clonealign and unassigned cells (see Methods, Table 1 and Additional file 2: Table S1). (e) Clone prevalence from sitka phylogenetic results in DLP+ analysis.


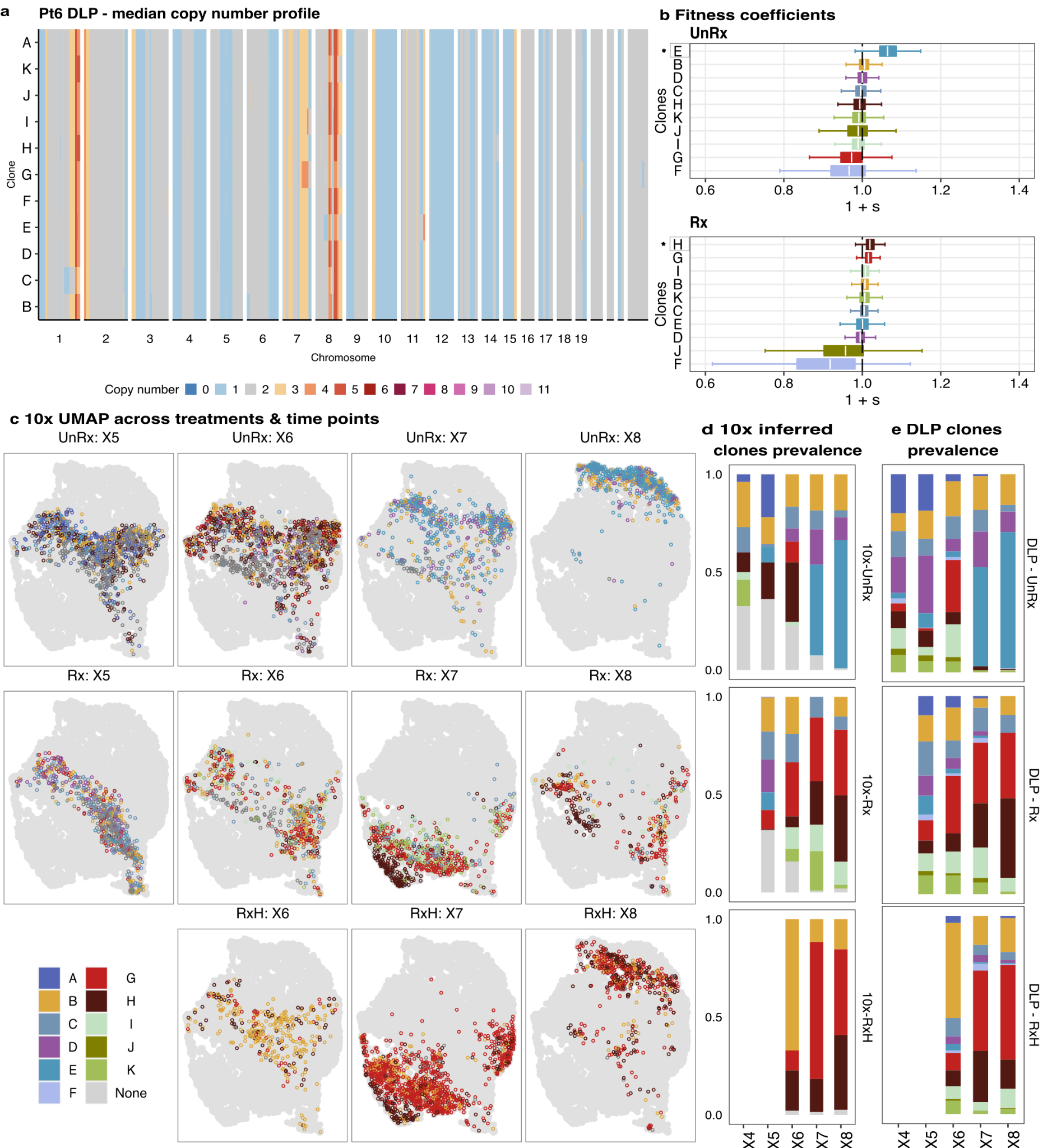


#### Figure S5: Genomic and transcriptomic characteristics of Pt6.

(a) Segment copy number profiles of Pt6 in DLP+ sitka phylogenetic tree results, heatmap with x-axis lists chromosome regions, y-axis lists the sitka phylogenetic clones. Each entry represents the median copy number at the corresponding bin genomic position for each clone. (b) fitness coefficient of each clone in DLP+ from previous analysis [[1]](https://paperpile.com/c/OLI0T7/uxVZ). The stars * mark the clones with highest fitness coefficient in untreated, and treated cells. (c) UMAP of 10x scRNA-seq data denote the landscape of cells across drug treatments, drug holiday and untreated time points. (d) Clone prevalence of inferred clones in scRNA-seq analysis. “None” includes cells filtered out by clonealign and unassigned cells (see Methods, Table 1 and Additional file 2: Table S1). (e) Clone prevalence from sitka phylogenetic results in DLP+ analysis.


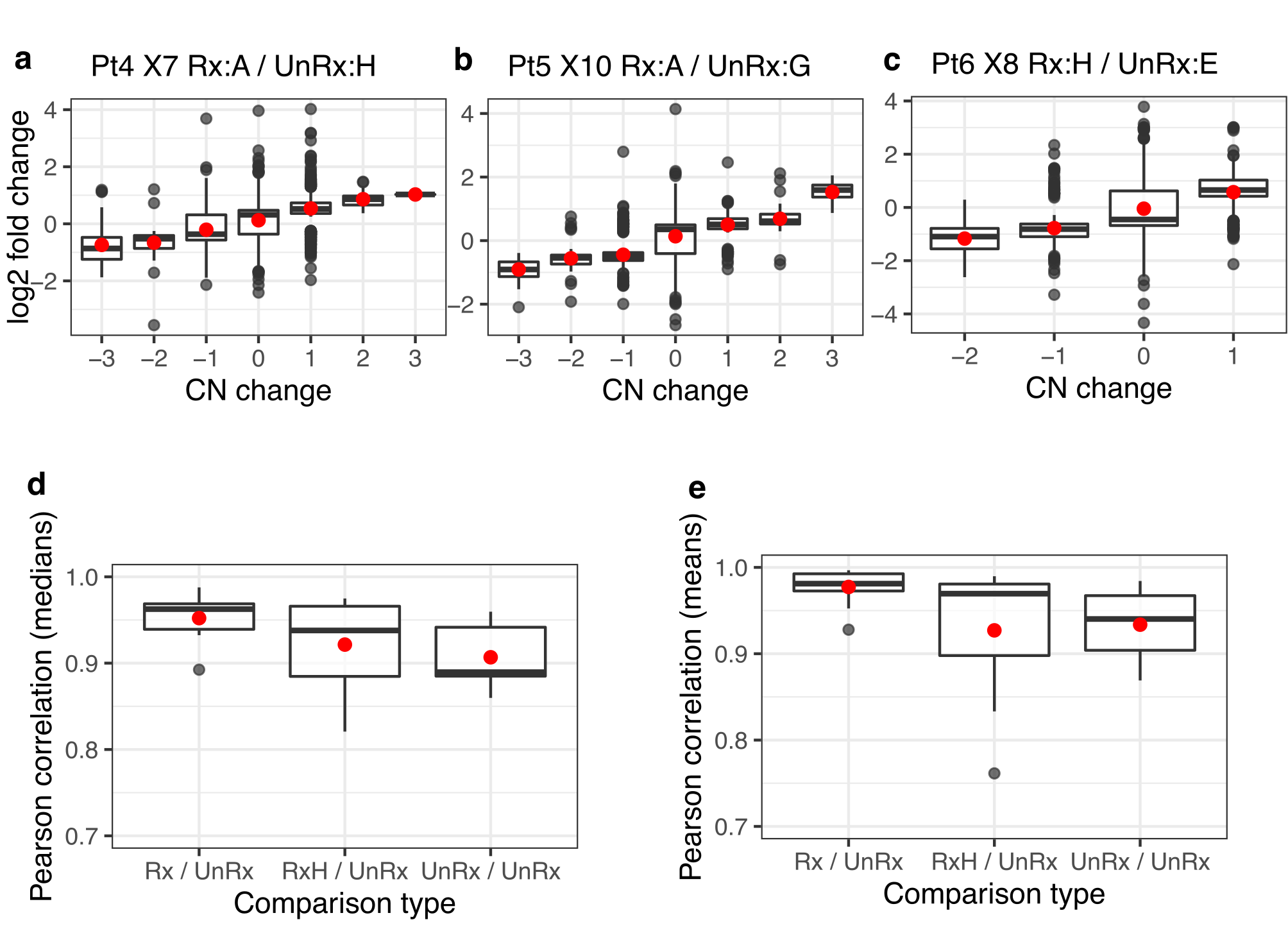


#### Figure S6: Correlation between copy number (CN) change and differential expression.

(a) log2 fold change for the corresponding CN change in Pt4 passage X7 when comparing the differentially expressed genes at Rx:A vs. UnRx:H, as in Figure 3c. The red dots depict means, the black horizontal lines depict medians. Pearson correlation for the medians versus CN change (Pmedians) = 0.99. Pearson correlation for the means versus CN change (Pmeans) = 0.99. (b) Same as a, but for Pt5 passage X10, Rx:A versus UnRx:G, as in Figure S7a. Pmedians=0.97, Pmeans=0.98. (c) Same as a, but for Pt6 passage X8, Rx:H versus UnRx:E, as in Figure S7b. Pmedians=0.95, Pmeans=0.99. (d) Boxplots of Pearson correlations for all DE comparisons in Figure 3d and Figure S8c,d, by comparison type, using Pmedians. (e) Same as e, but using Pmeans.

####
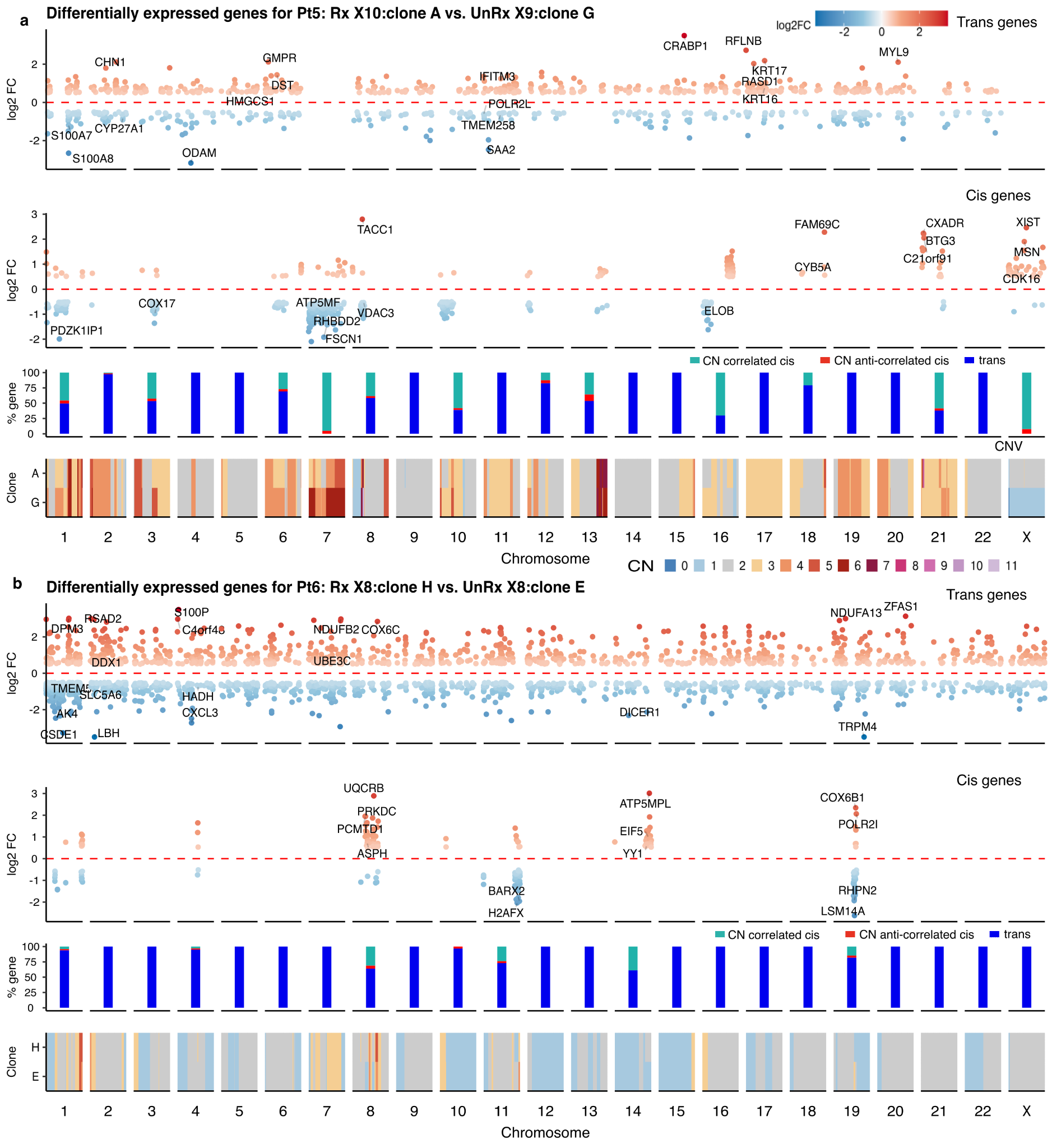


#### Figure S7: Track plots for Pt5 (a) and in Pt6 (b) showing the overlapping regions between gene genomic regions and copy number bin genomic regions, supplementary to Figure 3.

1. Pt5 comparing Rx passage X10 clone A against UnRx passage X9 clone G. (b) Pt6 comparing Rx passage X8 clone H against UnRx passage X8 clone E. In each panel, log2 fold change (FC) values of in-trans DE genes (top), in-cis DE genes (middle) are shown. Red and blue gradient colors denote the degree of log2 FC in positive and negative directions, each dot is one DE gene with selected condition abs(log2FC)>0.5, FDR<0.01, p value<0.05. Third panel: the distribution of each gene type across chromosomes. Genes are classified into copy number correlated/anti-correlated in-cis, and in-trans genes based on DE gene directions in scRNA-seq, and copy number values of pair clones at overlapping genomic regions. At the bottom, median copy number of each clone at bin genomic regions across chromosomes.

**
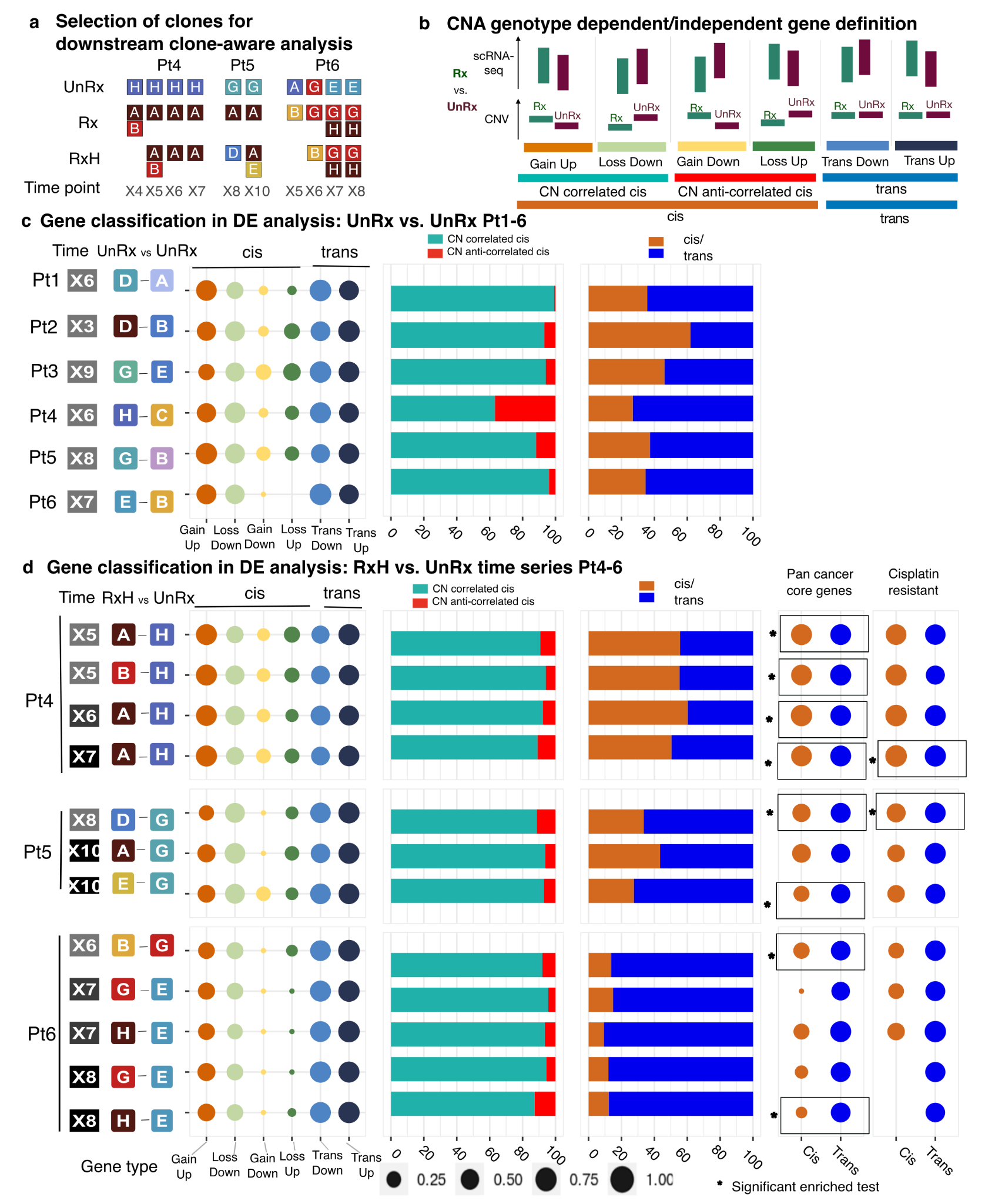
**

#### Figure S8: In-cis and in-trans gene proportions and gene set memberships for untreated series, and drug holiday time series, supplementary to Figure 3.

(a) For Pt4-6, the clones that were the fittest or most abundant and had at least 100 scRNA-seq cells were selected for further clone-aware analysis. (b) Gene classification: genes are divided into in-cis, and in-trans based on whether the copy number values changed - non uniform values at the overlapping genomic regions of the pair clones comparisons. Further, In-cis genes are divided into copy number correlated/anti-correlated in-cis genes based on the same or opposite directions of DE genes in scRNA-seq and copy number values. Genes were then classified based on the direction of change in gene expression (up- or down-regulated) as compared to the direction of change in copy number (gain or loss). (c) Differentially expressed genes between UnRx versus UnRx for Pt1-6 between the two most dominant clones in untreated patients Pt1-3 and untreated Pt4-Pt6. The dot size represents the proportion of each gene type based on the definition in b. (d) Differentially expressed genes between drug holiday clones versus sensitive clones (RxH versus UnRx) in Pt4-6. The dot size represents the fraction of each gene type based on the definition in (b). Copy number correlated in-cis (light blue) and copy number anti-correlated in-cis (red), in-cis genes (chocolate) and in-trans genes (blue). Gene set membership: mapping of in-cis and in-trans genes to two reference gene sets: Pan cancer core genes set [[2]](https://paperpile.com/c/OLI0T7/zsGqj) and Cisplatin resistance set - curated genes list from the latest literature with cisplatin resistance. Rectangles with star * show significant enrichment pathways of reference gene sets (p-adj values < 0.05).


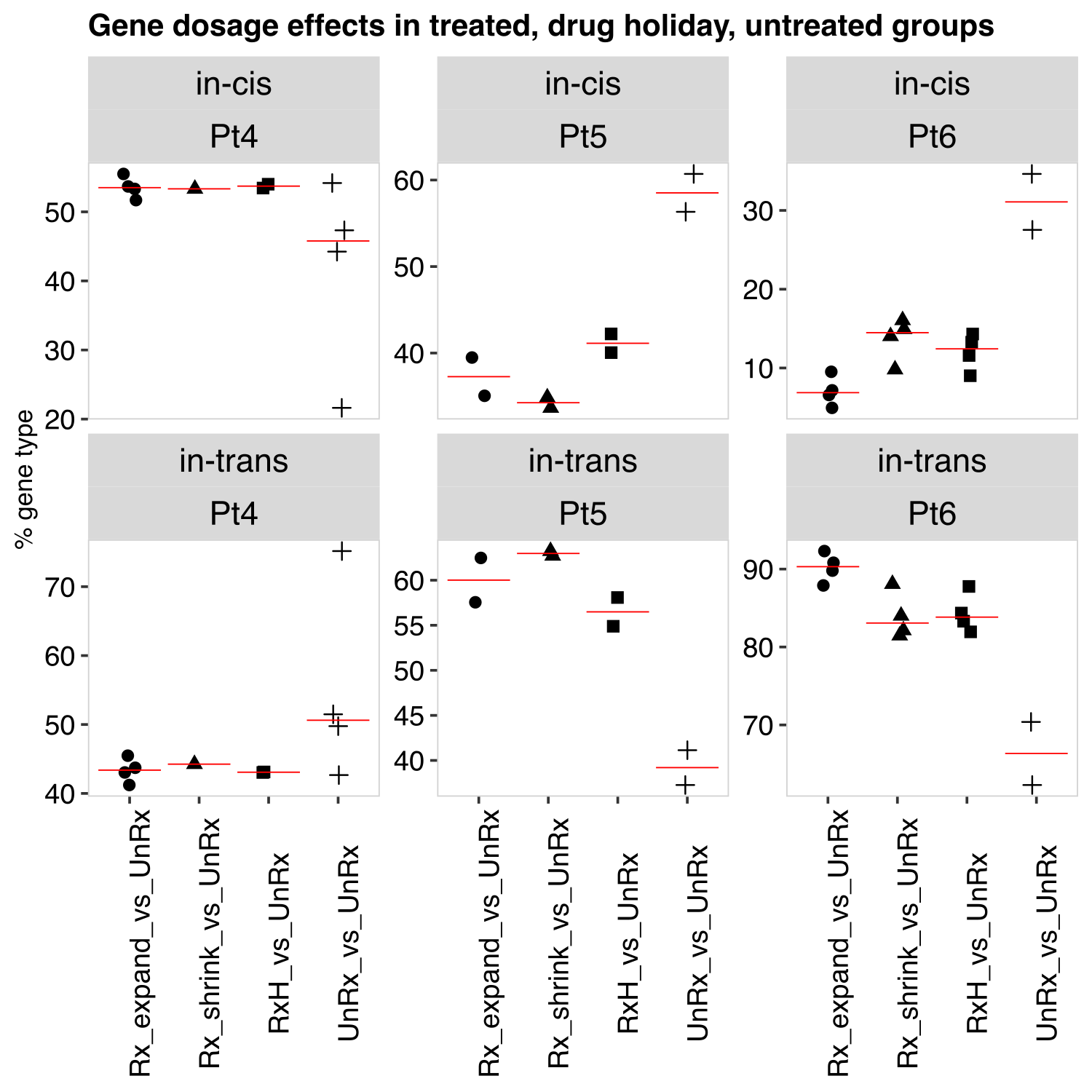


#### Figure S9: Summary of in-cis and in-trans genes across four categories of differential expression comparisons, supplementary to Figure 3.

In-cis (top row) and in-trans (bottom row) genes proportions in Pt4-6 for each type of comparison. X-axis: differentially expressed analyses are classified into 4 groups of comparisons: Rx expanding clones, Rx shrinking clones, RxH and UnRx, all versus UnRx clones. Red lines show medians for each group.


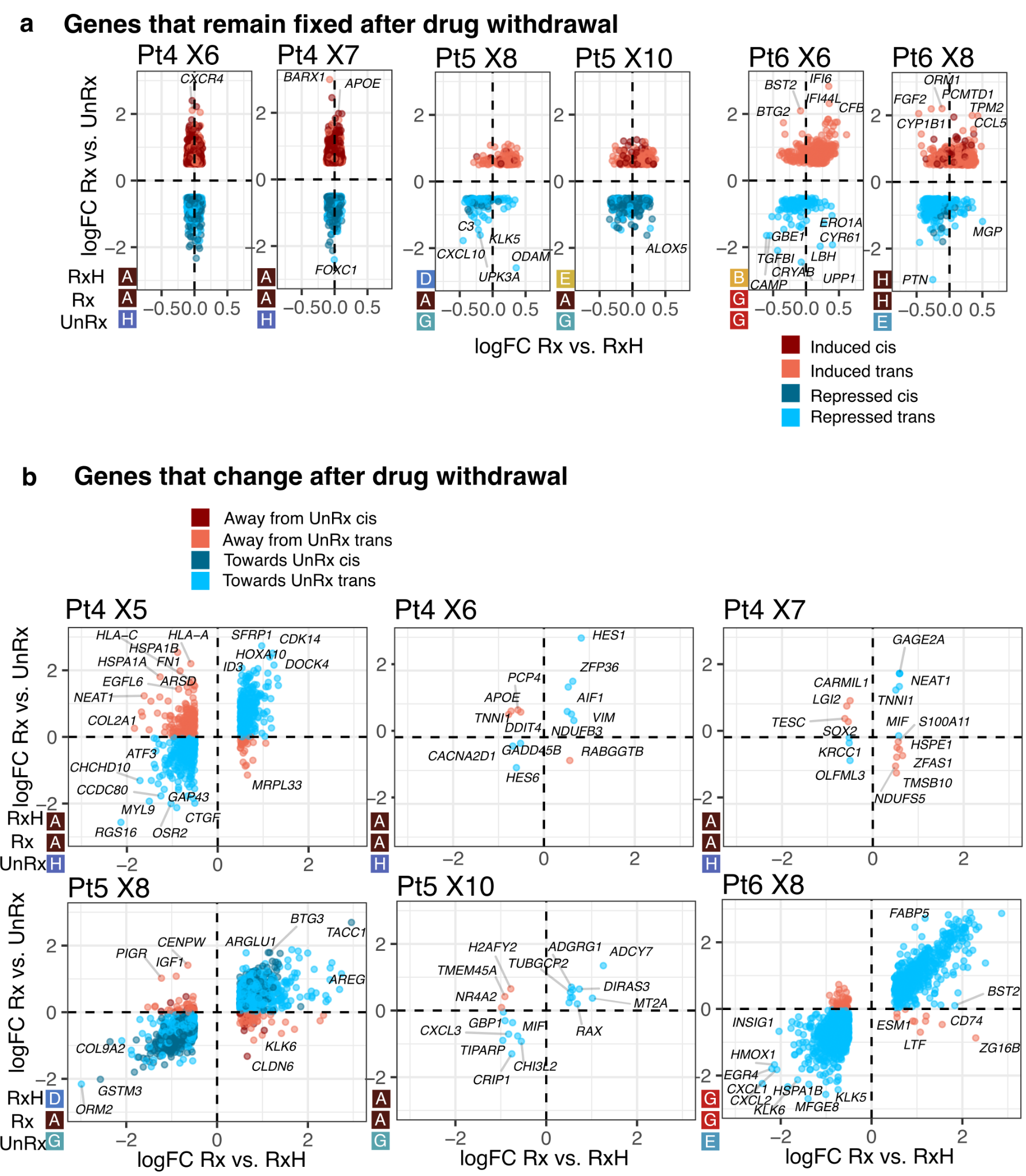


####

#### Figure S10: Dynamic genes scatter plots, supplementary panels to Figure 4.

(a) Genes that remain fixed after drug withdrawal, selected by intersecting the non-differentially expressed Rx vs. RxH genes (FDR > 0.1) with the differentially expressed Rx vs. UnRx genes (FDR < 0.01, |log2 fold change| = |logFC| > 0.5), as in Figure 4a,b. The panels show scatter plots for logFC of Rx vs. UnRx (y axis) against logFC of Rx vs. UnRx (x axis), for the selected clones, see also Figure 4b. Each point is a gene. (b) Genes that change after drug withdrawal, selected as differentially expressed at Rx vs. RxH (FDR < 0.01, |log2 fold change| = |logFC| > 0.5) and intersected with all the genes in Rx and UnRx, as in Figure 4a,e. The panels show scatter plots for logFC of Rx vs. UnRx (y axis) against logFC of Rx vs. UnRx (x axis), for the selected clones, see also Figure 4e.


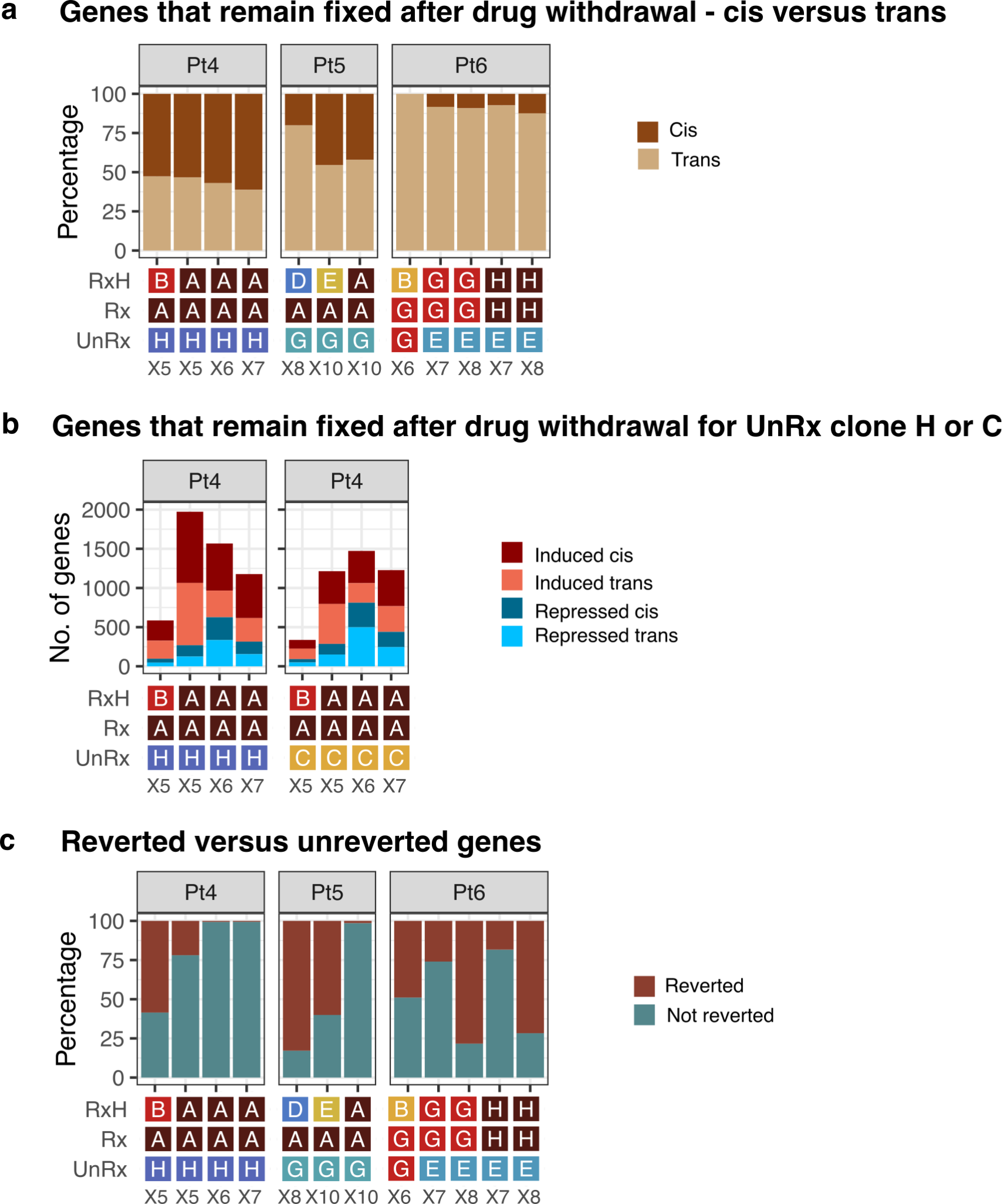


#### Figure S11: Dynamic gene comparisons, supplementary panels to Figure 4.

(a) Percentages of in-cis versus in-trans genes for the genes that remain fixed after drug withdrawal, as in Figure 4b. “In-cis” includes all the “induced” and “repressed” genes in-cis, “Trans” includes all the “induced” and “repressed” genes in trans. (b) Gene level comparisons for Pt4. Left panel: comparison of Rx and RxH clones against UnRx clone H, same as in Figure 4b left panel. Right panel: comparison against UnRx clone C. (b,c) X-axis: the same comparisons as in Figure 4b,e for Pt4-6. Y-axis: percentages. (c) Percentages for reverted versus non reverted genes for the same comparisons as in Figure 4b,e. Reverted genes include all the in-cis and in-trans genes in category “towards UnRx” in Figure 4e. Non reverted genes includes all the in-cis and in-trans genes that are “induced”, “repressed” and “away from UnRx” in Figure 4b,e.


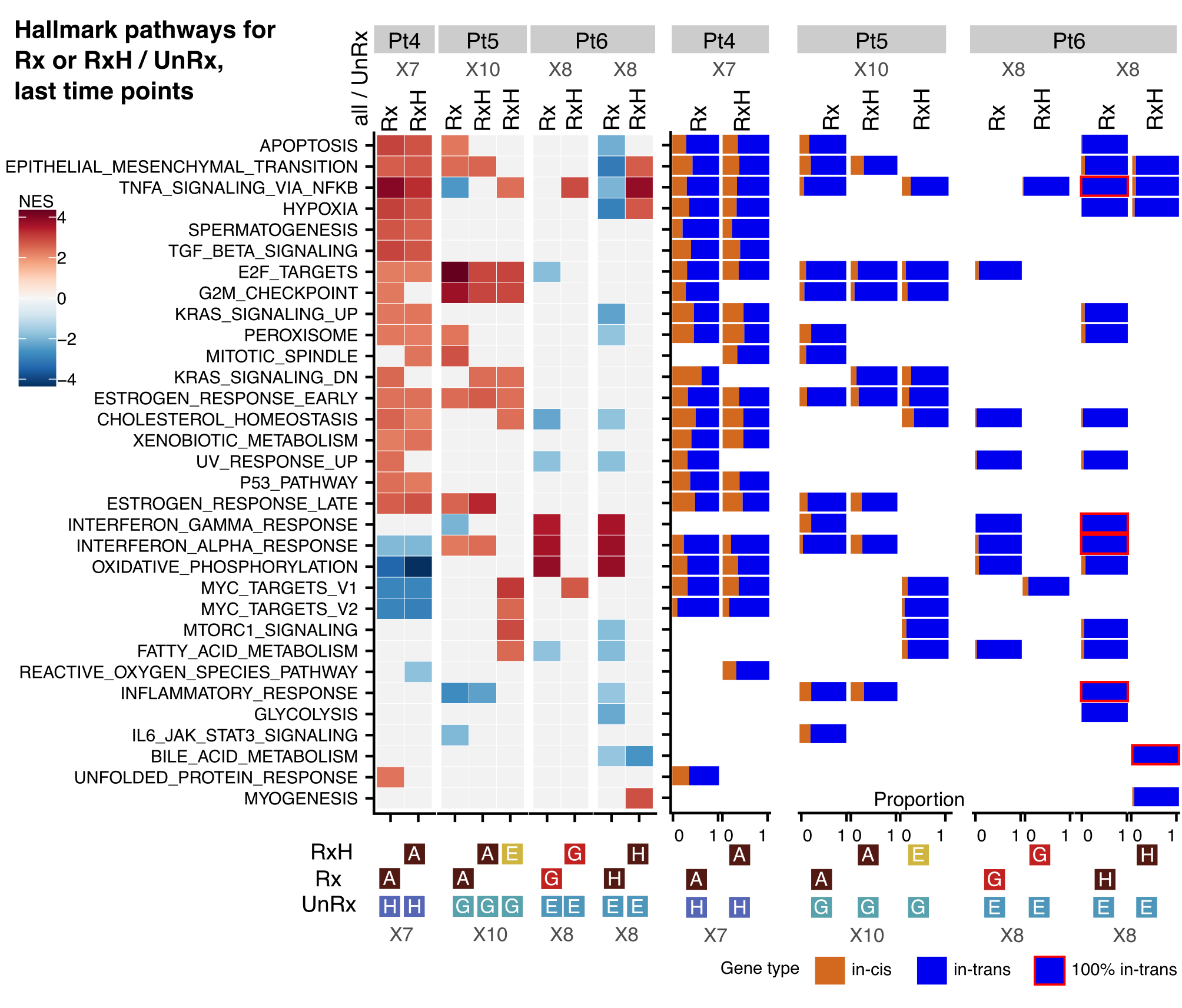


#### Figure S12: In-cis and in-trans gene proportions for the genes in the pathways at the latest time points for Pt4, Pt5 and Pt6, supplementary to Figure 5.

Left side: significantly enriched pathways (p < 0.05, vertical axis) from a ranked gene set enrichment analysis (GSEA) [[3]](https://paperpile.com/c/OLI0T7/DrqgM), using the Hallmark gene set collection from MSigDB [[3,4]](https://paperpile.com/c/OLI0T7/ME3tC+DrqgM), for Rx vs. UnRx and RxH vs. UnRx comparisons at the latest time points, same as in Figure 5. Right side: for each enriched pathway, showing the in-cis (orange) and in-trans (blue) proportions of all the differentially expressed genes (FDR < 0.01 and |log2 fold change| >= 0.25) that are part of the corresponding pathway. The red-contoured boxes represent cases where all the genes are in-trans and there are no in-cis genes.


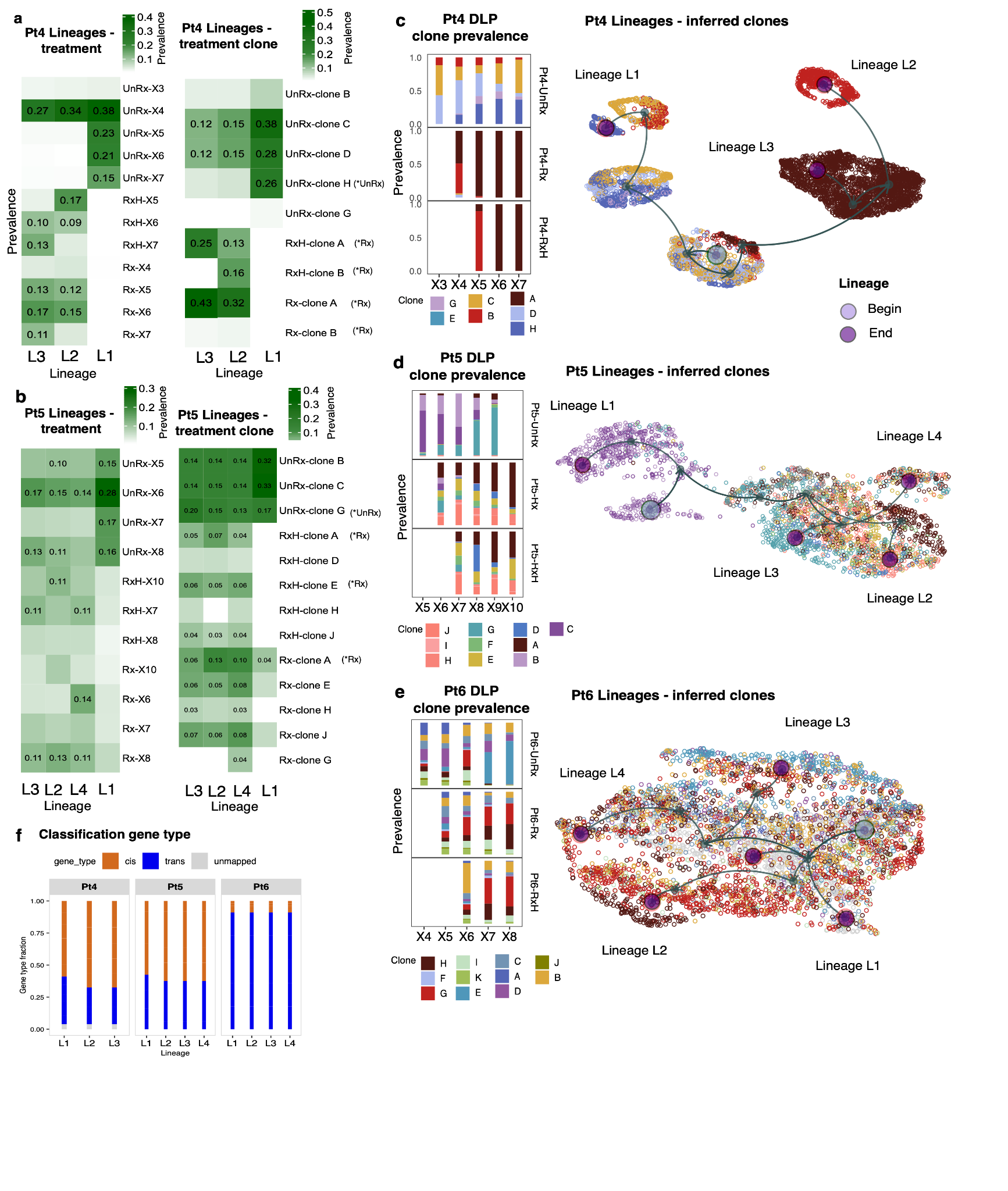


#### Figure S13: Characterizing each lineage based on the fraction of cells, fitted clone labels, and treatment conditions, supplementary to Figure 6.

(a) Pt4: lineage versus treatment comparison for Pt4. UnRx: untreated, Rx: treated, RxH: drug holiday. Lineage versus clone comparison for Pt4. *UnRx: clone that highly fitted in untreated condition, *Rx: clone fitted under drug, *RxH: clone fitted under drug holiday; In details, lineage L1 - Pt4: high prevalence in untreated cells UnRx; lineage L2-Pt4: large proportion of cells at drug holiday RxH passage X5, X6; lineage L3-Pt4: high prevalence in treated cells Rx and drug holiday late time point RxH passage X7. (b) Pt5: similar as Pt4, characterizing each lineage by treatment conditions, and cell clonal labels; In details, lineage L1 - Pt5: high prevalence in untreated cells UnRx; lineage L2-Pt5: large proportion of cells at first time treatment Rx passage X6; lineage L2-Pt5, L3-Pt5: high prevalence in treated cells Rx and drug holiday RxH. (c-e) Clonal prevalence in copy number analysis DLP+ data(left) and UMAP projection with embedded cell clonal labels and annotated lineages (right) for Pt4, Pt5, Pt6 respectively. (f) Mapping pseudotime significant genes into in-cis, in-trans, or unmapped gene types.


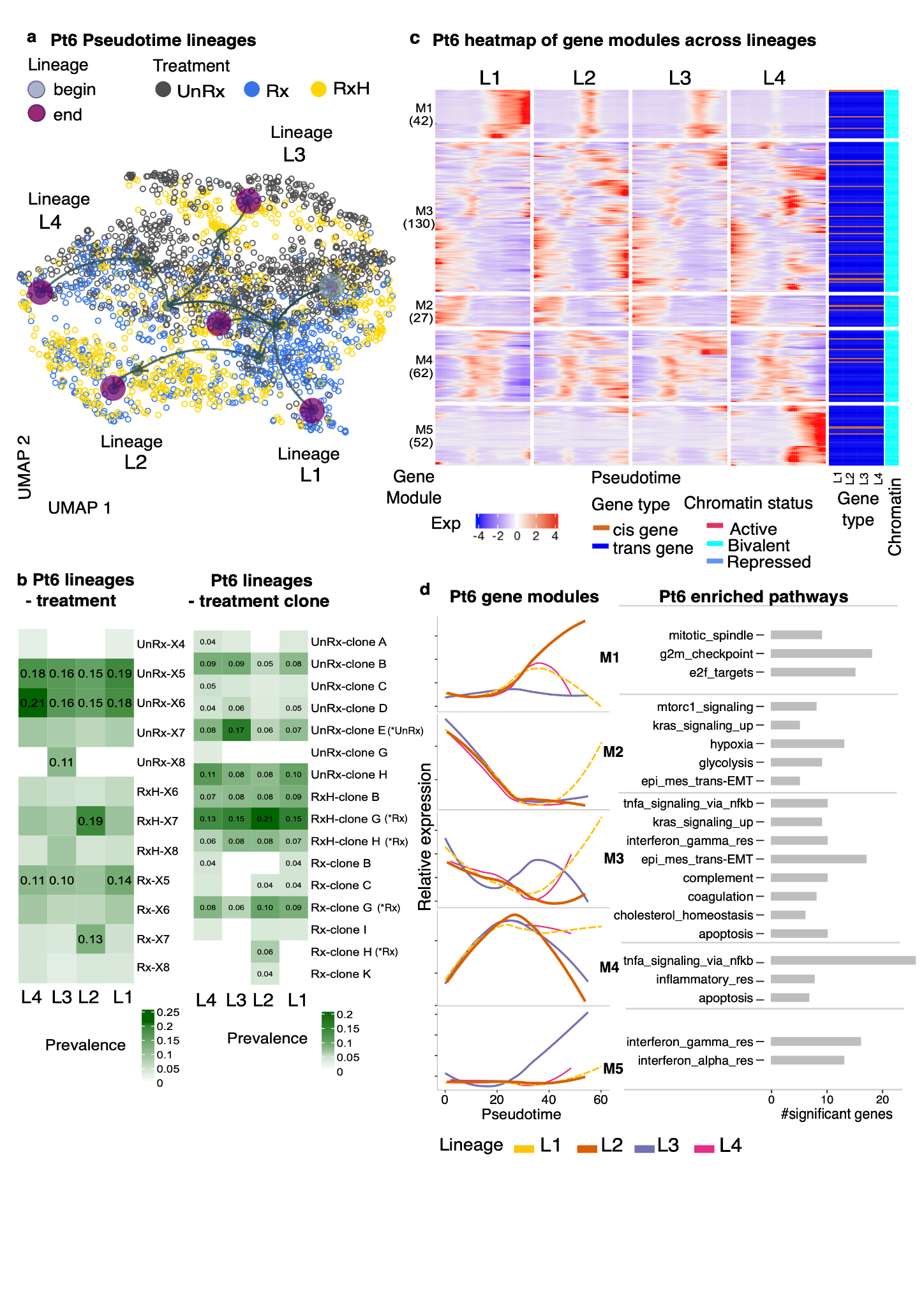


#### Figure S14: Pseudotime analysis of Pt6 showing dynamic gene regulation across treatment, drug holiday, and untreated time points, supplementary to Figure 6.

(a) UMAP visualization of cells in all lineages - output of pseudotime analysis coloured by actual drug treatment status (UnRx: untreated cells, Rx: drug treatment, RxH: drug holiday, and time point X (i.e. X4, X5,...). Individual lineage with its starting point - green, and end point - red circles. (b) Characterization of pseudotime lineages based on cell prevalences from actual drug treatment status and from clone labels assigned to cells in each lineage. Clones with high fitness coefficient are noted (*UnRx, *Rx). (c) Heatmap of gene expression across different lineages. X-axis: smoothed gene expression in each lineage, color denoting gene expression level, Y-axis: individual gene and grouped by regulatory genes modules (M is gene module) and number of genes in each module, ex: M1(42). Right: gene types of each individual gene across different lineages, orange: in-cis, dark green: in-trans. Chromatin status of each gene module: active - red, bivalent-cyan, repressed-blue. (d) Summary of relative gene expression for individual lineages in each gene module from heatmap panel c and list of hallmark enriched pathways related to each gene module based on enrichment analysis gprofilers statistical tests with P-adj<0.05, the size of bar denotes number of gene in each gene module that belong to significant hallmark genes set.

##

## References

[1. Salehi S, Kabeer F, Ceglia N, Andronescu M, Williams MJ, Campbell KR, et al. Clonal fitness inferred from time-series modelling of single-cell cancer genomes. Nature. 2021;595:585–90.](http://paperpile.com/b/OLI0T7/uxVZ)

[2. Forbes SA, Bindal N, Bamford S, Cole C, Kok CY, Beare D, et al. COSMIC: mining complete cancer genomes in the Catalogue of Somatic Mutations in Cancer. Nucleic Acids Res. 2011;39:D945–50.](http://paperpile.com/b/OLI0T7/zsGqj)

[3. Shi J, Walker MG. Gene Set Enrichment Analysis (GSEA) for Interpreting Gene Expression Profiles. Curr Bioinform. 2007;2:133–7.](http://paperpile.com/b/OLI0T7/DrqgM)

[4. Liberzon A, Birger C, Thorvaldsdóttir H, Ghandi M, Mesirov JP, Tamayo P. The Molecular Signatures Database (MSigDB) hallmark gene set collection. Cell Syst. 2015;1:417–25.](http://paperpile.com/b/OLI0T7/ME3tC)
