# Supplementary material for: Single-cell decoding of drug induced transcriptomic reprogramming in triple negative breast cancers
Source: Genome Biol. 2024 Jul 18;25:191. doi: 10.1186/s13059-024-03318-3 (PMC11256464; doi:10.1186/s13059-024-03318-3)
Supplement: Supplementary file 3 — Additional file 3. Review history. [file 13059_2024_3318_MOESM3_ESM.docx]

Review History

**First round of review**

**Reviewer 1**

**Were you able to assess all statistics in the manuscript, including the appropriateness of statistical tests used?**

Yes, and I have assessed the statistics in my report.

**Comments to author:**

In this study, Kabeer et al. generated serially passaged tumors using PDX model and the xenografts were treated in different strategies: untreated, cisplatin or cisplatin holiday. The main novelty is facilitated by single-cell transcriptomic analysis to each sampling series and tracking of drug induced transcriptome changes, which led to discovery of cisplatin-resistance involved gene regulation. They also assigned cells to genotypic subclones and found that transcriptional alterations associated with drug resistance could arise from both genomic and non-genomic mechanisms. These findings offer insights into how polyclonal tumors respond to cisplatin treatment.

One concern from this reviewer is that these findings are based on PDX models derived from 6 tumor samples with several passages, which might limit the reproductivity of the results to a broader spectrum of TNBC patients.

And the following are specific comments that needed to be addressed to improve the clarity of the manuscript:

What is the resolution/bin size for identifying CNA genotype of each sample?

In Figure 2e, what does "clonal proportion" indicate? Except for the proportion level, can the authors see the bin-level copy number correlations for clonealign 10x and DLP?

What are major impacts of genomic transcriptional pattern and non-genomic transcriptional plasticity, and how might they be relevant in a clinical context?

Plots in Figure 1b do not straightforwardly align with the description in Results, for example, Pt4 has "intermediate fraction of altered genome (mean=0.11, sd=0.07)", but the plots may only show the median instead of mean which deviates from 0.11.

**Reviewer 2**

**Were you able to assess all statistics in the manuscript, including the appropriateness of statistical tests used?**

Yes, and I have assessed the statistics in my report.

**Comments to author:**

The study is positioned to dissect the relative contribution of copy number alteration (CNA) versus (presumed) non-genetic factors, to chemotherapy resistance in triple negative breast cancer (TNBC). This is an interesting and important topic, and timely with the growing interest in plasticity in the field.

The study makes use of 6 PDX models that are serially passaged over 2.5 years in mice, and extremely extensive single cell sequencing (>100K cells). The analysis is extensive. The highlighted findings are:
- Subclones with distinct patterns of CNAs have distinct responses to chemotherapy (i.e. variable fitness under treatment).
- The fittest CNA-defined clones have consistent gene expression patterns, whereas gene expression is more variable in less fit clones.
- An EMT phenotype associates with resistance.
- Cells do not return to their original gene expression pattern when therapy is withdrawn, indicating "hysteresis" in the transcriptional response (which I presume is due to unmeasured genetic or epigenetic evolution that "locks in" the new gene expression pattern).

The nomenclature about the clones and treatment sensitivity is a bit confusing. I don't think the rxH group is defined in the main text -> how long was this population out of drug for?

I've really struggled to understand the definition of in-cis and in-trans transcripts. I thought that these were transcripts in regions where a new CNA was acquired, and subsequent gene expression differences compared between the parent and offspring clone. But most comparisons are between untreated and treated PDXs, where the "parent-child" relationship might not exist (i.e both the parent and child could be unique to one condition), so I think my understanding might be wrong. Could the authors revise the definitions/terminology here?
Might labelling the transcripts "within a new CNA" or "within regions of uniform copy number" be simpler?
Relatedly, how the ancestral relationship between clones was used in the analysis wasn't clear to me. Is it actually ignored?

Related, p9-line13 - the phrase "outside of clonal CNA differences" is very confusing to me, do the authors mean "in genomic regions with uniform copy number across all clones"?

I couldn't see a definition of "canonical cis transcripts" (fig 2d)

What is the definition of a "fitness gene"? Is more or less expression of these genes associated with increased fitness (and how is fitness measured?)

The dynamics of gene expression changes through treatment (end of p10 and top of p11) is interesting, though unfortunately I found the terminology challenging again. How is "stably expressed" defined? (I thought it must be assessed by looking at the same clone across time, but that seems to be a distinct analysis in a later paragraph). The authors also use the phrase "stable drug expression" but I presume that is a typo

The reports that chemo challenge tends to generally broadly switch on gene expression, and then expression decays towards pretreatment values, is very interesting and illustrates the plasticity of the gene expression response. Can the authors say anything about differential sensitivity of these "reverted" clones to subsequent rounds of treatment? Are they equally sensitive to treatment once the gene expression has returned to "baseline"? Functional experiments here would be wonderful (drug testing ex vivo) but I think are unreasonable to expect the authors to perform.

Two cases (in patients 4&5) where a clone with distinct CNAs sweeps during treatment are highlighted, and in these cases expression doesn't revert to the pretreatment state. It wasn't clear to me whether or not the fixed change in expression was simply because of the gene dosages expect from the CNA, or if there were genome wide (trans?) differences in gene expression. Presumably the latter but could this be clarified?

The dynamics of reversion to a pre-treatment gene expression state are also interesting - and the notion of "transcriptional memory". Could the authors speculate on the mechanism, is it likely genetic or epigenetic? (or something else)

I felt the pseudotime analysis sits uneasily with the earlier analysis, as in the pseudotime analysis temporal dynamics are inferred, but the data has actually directly measured changes over time and through evolution (and these analyses could detect dynamics, such as osscilations that would presumably not be detected in pseudotime analysis). I wonder if the manuscript (i.e. narrative) would be clearer without this section.

It occurred to me that there is selection in the experiment to both the drug and the host mouse. The analysis addresses the first in detail, but can host selection be delineated too?


Minor points:

- What was the initial death rate upon drug treatment? (IC50 value, etc) Would be nice to know to understand the strength of selection in the experiment.
- The abstract states the paper contains 126,566 single cell transcriptomes, but only 55,025 survived QC. The smaller number feels fairer to report.
- What's the time points in a timeline? Passage numbers are given, but absolute time would nice to know.
- Were the PDX performed in replicates? If there are some replicates the repeatability of evolution would be great to know.

**Reviewer 3**

**Were you able to assess all statistics in the manuscript, including the appropriateness of statistical tests used?**

Yes, and I have assessed the statistics in my report.

**Comments to author:**

Kabeer et al. presented a study of transcriptional drug responses using PDX models of TNBC. While this study proposed an interesting single cell re programing model, the data support is relative weak, particularly, the only three treated PDX Pt had completely different clonality. The analysis of cis and trans- dosage effects is shallow. The study could be much improved through more in-depth analyses.

1. In the alignment of DLP and scRNAseq data, there exist large portions of unassigned cells, indicating sampling bias of the two approaches and or alignment errors. Could the author explore more?

2. The clonal compositions of three treated patients were very different. What are the underlying factors, particularly, Pt 4 had one dominant clone after treatment while the other two had many co-existing clones?

3. The untreated samples also showed one-directional clonal enrichment in all three patients. The authors compared them to Rx enriched clones. However, are there shared features among them? Are they due to random drift?

4. Fig 2e figure legends didn't match with axis labels, and both were poorly stated. The r values labeled in figure were not clearly explained. It's surprisingly high that gene expression and copy numbers had up to 93% concordance.

5. It isn't clear if copy numbers had strong dosage effects in treatment response genes. The in-cis transcript accounts for 5.1-57.4% of DEG across selected clones in three treated PDX-Pt 4,5,6. What are the in-cis percentages of any clone pairs in un-treated PDX samples? What are the in-trans transcript percentages in treated group? Overall, what are the percentages of copy number in-cis transcripts in treated groups comparing to un-treated groups? Would dosage effect stronger or weaker in expanded clones compared to other shrinking clones?

6. Could anti-correlated transcripts arise from other regulations, such as epigenetics in addition to copy number miscalling?

7. "We asked which cellular functions are represented by the cis- and trans- encoded transcription dynamics to drug exposure and withdraw". However, the whole paragraph didn't provide results regarding which genes are cis- and trans-encoded by copy number events. For instance, it will be interesting to learn if copy number changes converge to EMT related phenotypes (more cis-regulated than trans-regulated).

8. How about the alignment between pseudo-time lineages and CNV lineages?

Minor:

1. Fig 1a - there wasn't annotation of light blue circle

2. All gene names need to italic

3. Many words in sub-panels can be moved to figure legends.

**Authors Response**

**Point-by-point responses to the reviewers’ comments:**

We thank the reviewers for constructive comments and feedback. Below we address point by point the revisions to the manuscript. We have added new supplementary figures and tables to address specific comments and in addition revised the text. Text revisions are shown in blue font in the rebuttal and in the revised text. Comments in response are shown in green font. As some comments were in common, we refer to the section in the rebuttal where they are first addressed. Reference numbers in this document refer to publications listed in this same document and may not match the reference numbers from the manuscript.

—-----------------------------------------------------------------------------------------------------

# Reviewer #1:

In this study, Kabeer et al. generated serially passaged tumors using PDX model and the xenografts were treated in different strategies: untreated, cisplatin or cisplatin holiday. The main novelty is facilitated by single-cell transcriptomic analysis to each sampling series and tracking of drug induced transcriptome changes, which led to discovery of cisplatin-resistance involved gene regulation. They also assigned cells to genotypic subclones and found that transcriptional alterations associated with drug resistance could arise from both genomic and non-genomic mechanisms. These findings offer insights into how polyclonal tumors respond to cisplatin treatment.

1.1 One concern from this reviewer is that these findings are based on PDX models derived from 6 tumor samples with several passages, which might limit the reproductivity of the results to a broader spectrum of TNBC patients.

Response:

Our work emphasizes a proof of concept, the joint analysis of gene dosage mutations and transcription at single cell resolution, on the background of temporally measured clone fitness. This approach is necessary to separate plasticity, from genomic encoding of transcriptional variability in genomically unstable cancers. The generalization of the specific findings, for example on clone-specific pathway determinants, would indeed require a larger study, but that is not the principal question being addressed. The key results reveal how gene dosage and transcriptional plasticity can be dissected through joint temporal sampling and show the degree to which plasticity and genomic variants contribute. We tried to obtain scRNA-seq data from [[1]](https://paperpile.com/c/SxU1B4/3KVP) but the authors were not willing to share the primary data. We are not aware of additional data sets where densely sampled time series of solid tumours where gene dosage related clones and transcription were co-measured. We acknowledge these limitations and discuss them more fully.

We have added the following discussion elements to the manuscript:

(i) We note that utilization of single-cell DNA and RNA sequencing techniques in solid tumors has facilitated the phylogenetic reconstruction of tumor lineages [[2–4]](https://paperpile.com/c/SxU1B4/QJDV+qFFT+3HX8). It has also resolved rare subpopulations [[5,6]](https://paperpile.com/c/SxU1B4/e9u2+at0n) and offered insights into the phenotypes of stromal and tumor cells in various cancers ([[7–10]](https://paperpile.com/c/SxU1B4/wbpP+mEO4+DPRe+DUjM). The majority of these publications focused on either DNA or RNA exclusively. These studies did not explore the analysis of both RNA and DNA at the single-cell level within the same sample, nor did they examine changes in gene dosage affecting gene expression at the clonal level. (added to introduction text)

(ii) The clonal fitness and transcriptional states we observed converge with known mechanisms of chemoresistance in cancers. We observed that the four common pathways identified in all three serially passaged and cisplatin-treated deep xenografts are also present in other cancers subjected to cisplatin treatment. Transcriptomic profiles of cisplatin-treated small cell lung carcinomas[[11]](https://paperpile.com/c/SxU1B4/SPLty) revealed similar pathways (Hypoxia, EMT, TNFA-signaling, Interferon signaling) to those identified through time series sampling. A recent single cell study of different chemotherapeutic agents[[12]](https://paperpile.com/c/SxU1B4/CcLNW) also suggested pathway convergence. In this study, samples were collected pre-treatment, mid-treatment, and post-treatment, involving a combination of drugs including doxorubicin, Taxol, and VEGF inhibitors. Despite differences in the drug combinations and sampling methods, we observed common pathways shared with our findings. Notably, the relationship between copy number and the proportionality of transcripts at the clonal level was not explained in the latter study. (added to discussion)

(iii) The proportionality of genome altered by dosage mutations is entirely reflective of TNBC as we have shown in larger studies (Shah et al, Nature 2012, Funnel et al Nature 2022) and the patient PDX described here are reflective of that spectrum. We have made this point clearer in the discussion.

And the following are specific comments that needed to be addressed to improve the clarity of the manuscript:

1.2 What is the resolution/bin size for identifying the CNA genotype of each sample?

Response: The bin size and resolution for CNAs is 500 kilobases, a reflection in part of the depth of sequencing as we have previously described. This information is now clearer in Methods, see section: “Copy number estimation, phylogenetic tree inference, clone determination and clonal abundance measurements in DLP+”, first paragraph:

“We applied the DLP+ single cell analysis pipeline that was developed in the previous study [[13,14]](https://paperpile.com/c/SxU1B4/iM7DP+0ZC0K) for single cell DNA sequencing datasets. Firstly, the read data from DLP+ single cell sequencing was preprocessed using HMMcopy package version 1.32.0 to provide a copy number profile for each cell with 500Kb fixed-length non-overlapping genomic regions [[15]](https://paperpile.com/c/SxU1B4/iFqOw).”

1.3 In Figure 2e, what does "clonal proportion" indicate? Except for the proportion level, can the authors see the bin-level copy number correlations for clonealign 10x and DLP?

Response: We have now clarified the “clonal proportion” definition in the legend of Figure 2e, as follows:

“Each point shows the *clonealign*-inferred scRNA-seq clonal proportion (y-axis) versus the corresponding DLP+ clonal proportion (x-axis) for each clone in each sample. Each patient is denoted by a different shape. Pearson correlation coefficients for each patient are reported at the top.“

We also report the gene-level copy number - gene expression correlations in Supplementary Figure 6.

1.4 What are the major impacts of genomic transcriptional pattern and non-genomic transcriptional plasticity, and how might they be relevant in a clinical context?

Response: We have added the following to clarify, in the discussion:

Transcriptional plasticity implies short term responses to drug treatment which may be reversible. We observed that a large part of the transcriptional response is not fixed. On the other hand*,* gene dosage mutations are unlikely to revert to ground state, especially where deletions occur. We showed that *in cis* genes (whose expression may be influenced by gene dosage) are less prone to variation, depending on the nature of the change. Complete deletion is irreversible, gene amplification could progress in either direction (more gains or loss after gain). Once a resistant (genomic) clone having *in cis* genes, becomes dominant and fixed in the tumour population, the possibility of clonal competition with less fit precursors no longer exists. Potential clinical significance lies in the degree of reversibility of each mechanism. We previously showed that clonal dominance of resistance (i.e. where gene dosage mutations may carry some of the fitness) results in irreversible states. On the other hand, where clonal dominance does not occur, reversal of clone determined fitness may occur during treatment holiday and patients' tumours could remain sensitive to re-challenge.

1.5 Plots in Figure 1b do not straightforwardly align with the description in Results, for example, Pt4 has "intermediate fraction of altered genome (mean=0.11, sd=0.07)", but the plots may only show the median instead of mean which deviates from 0.11.

Response: As we understand, the reviewer means Figure 2b, instead of Figure 1b. Indeed, the means are very different from the medians in these cases. To avoid confusion, we are now reporting median values instead of mean values as follows:

“Pt4 exemplifies a low complexity (6 clones) and intermediate fraction of altered genome (median=0.33, sd=0.16), Pt6 a high clone complexity (11 clones) and low fraction of altered genome (median=0.04, sd=0.02), whereas Pt5 exhibited high clonal complexity (10 clones) and high altered genome fraction (median=0.27, sd=0.16), [Figure 2](https://docs.google.com/document/d/19YPI2Ch3hqwdPje_8lKfijphZY9fhh7B3mjD_z9R1oM/edit#fig_DLPtree)a,b.”

—-----------------------------------------------------------------------------------------------------

# Reviewer #2:

The study is positioned to dissect the relative contribution of copy number alteration (CNA) versus (presumed) non-genetic factors, to chemotherapy resistance in triple negative breast cancer (TNBC). This is an interesting and important topic, and timely with the growing interest in plasticity in the field.

The study makes use of 6 PDX models that are serially passaged over 2.5 years in mice, and extremely extensive single cell sequencing (>100K cells). The analysis is extensive. The highlighted findings are:

- Subclones with distinct patterns of CNAs have distinct responses to chemotherapy (i.e. variable fitness under treatment).

- The fittest CNA-defined clones have consistent gene expression patterns, whereas gene expression is more variable in less fit clones.

- An EMT phenotype associates with resistance.

- Cells do not return to their original gene expression pattern when therapy is withdrawn, indicating "hysteresis" in the transcriptional response (which I presume is due to unmeasured genetic or epigenetic evolution that "locks in" the new gene expression pattern).

2.1 The nomenclature about the clones and treatment sensitivity is a bit confusing. I don't think the rxH group is defined in the main text -> how long was this population out of drug for?

Response:

RxH was initially introduced in the first sentence of the results section under the term "cisplatin holiday." We have now incorporated the abbreviations UnRx (untreated), Rx (treated with cisplatin), and RxH (cisplatin+holiday) in the same sentence:

“neutral (no drug intervention) conditions (UnRx) and with parallel cisplatin treatment (Rx) and cisplatin holiday (RxH) conditions”.

Additionally, RxH is introduced in both Figure 1 and Table 1. The legend of Figure 1 now reads:

“(a) Tumor biopsies from 6 TNBC patients were transplanted in immunodeficient mice. Three TNBC untreated time-series (Pt1-3) and three cisplatin treated time-series (Pt4-6) with its counter drug holiday samples. UnRx: Grey-untreated, Rx: Blue-cisplatin treated, RxH: Yellow-cisplatin drug holiday (kept untreated for that cycle of treatment).”

At each passage, the treated tumour cells (Rx) have a break before the next cycle - this is in keeping with the way humans are treated. The holiday population (RxH) was without the drug for the same duration as the parallel mice treated with cisplatin for one cycle. RxH was subjected to one, two, or three cycles of cisplatin but entered a drug-free or holiday state in the corresponding passage, where they are compared with tumors continuously treated with cisplatin and those entirely treatment-naive. The RxH are parallel transplants that never again receive drugs and are allowed to grow to the endpoint.

We have also added more details in the Methods section "TNBC PDX timeseries treatment with cisplatin":

“In the re-transplant, all implanted mice were left without drug treatment for a period of 4-5 weeks until visible tumours formed and then half the mice were re-treated (Rx arm) and half left to grow further to endpoint - the latter harvested as the holiday interval (RxH) tumours, this is in keeping with the way humans are treated. Although we did not sample gene expression immediately before treatment started (this would have been destructive to the tumours), the observed responses of clones in the treated mice reflects a drug-free period before treatment started, mirroring the way chemotherapy is delivered to patients. The holiday population (RxH) was without the drug for the same duration as the parallel mice treated with cisplatin for one cycle. RxH was subjected to one, two, or three cycles of cisplatin but entered a drug-free or holiday state in the corresponding passage, where they are compared with tumours continuously treated with cisplatin and those entirely treatment-naive. The RxH are parallel transplants that never again receive drugs and are allowed to grow, to establish the full reversion status of the transcriptome after the prior treatment.”

2.2 I've really struggled to understand the definition of in-cis and in-trans transcripts. I thought that these were transcripts in regions where a new CNA was acquired, and subsequent gene expression differences compared between the parent and offspring clone. But most comparisons are between untreated and treated PDXs, where the "parent-child" relationship might not exist (i.e both the parent and child could be unique to one condition), so I think my understanding might be wrong. Could the authors revise the definitions/terminology here?

Might labelling the transcripts "within a new CNA" or "within regions of uniform copy number" be simpler?

Response: Indeed, in-cis genes are within CN differences, and in-trans genes are within regions with uniform copy number. We have now added a very simple cartoon to Figure 1, as follows:

Regarding the comparisons, our main focus was to compare the impact of drug treatment or holiday. In Results, section “Copy number variation …”, paragraph 6, we now made it clear that the differential expression was done between clones at the same passage (for example a clone from treated, passage X5, versus a clone from untreated, also passage X5), and not between offspring and parent because the tumor evolution occurs over time:

“Next, having established CNA clone identity of transcriptomes, we were interested in exploring the transcriptional change between distinct clones or distinct treatment status at the same time point (passage). Therefore, we investigated the differential transcription among same-passage clones that had the highest fitness coefficient [[13]](https://paperpile.com/c/SxU1B4/iM7DP) in different treatments (e.g., treated versus untreated, or drug holiday versus untreated)”

Further, in paragraph 7, we better explained the definitions for in-cis and in-trans transcripts:

“Having obtained the differentially expressed (DE) genes for an inferred clone pair (e.g., the comparison in [Figure 3](https://docs.google.com/document/d/19YPI2Ch3hqwdPje_8lKfijphZY9fhh7B3mjD_z9R1oM/edit#fig_manhattan)c for Pt4, passage X7, treated inferred clone A versus untreated inferred clone H), we next divided the DE genes based on whether they are encoded in genomic regions of copy number difference between clones: we refer to in-cis, where the copy number values are different between the two clones compared, and in-trans, where the copy number values are the same ([Figure 3](https://docs.google.com/document/d/19YPI2Ch3hqwdPje_8lKfijphZY9fhh7B3mjD_z9R1oM/edit#fig_manhattan)b). Precisely, first we extracted the genomic coordinates of each DE gene, and we mapped its genomic coordinates to the most overlapping genomic region in the DLP+ copy number data. Then, we calculated the median copy number across all cells in each clone, and compared the two medians. If they were different, then the DE gene is defined as an in-cis gene, otherwise it was defined as an in-trans gene. “

2.3 Relatedly, how the ancestral relationship between clones was used in the analysis wasn't clear to me. Is it actually ignored?

Response: The ancestral history was used to derive the phylogenetic tree, and cutting the tree defines the clones and the clades (tree branches). In our earlier paper Salehi et al, 2021 [[13]](https://paperpile.com/c/SxU1B4/iM7DP), we use this approach to define the subpopulations of clones whose fitness was estimated with a Wright-Fisher model. Most of the analysis in the current paper is focused on the comparison between the fittest clones in drug treatment (resistant) versus most fitted clones in the untreated conditions (sensitive). However, there is a distinct relationship between the resistant and sensitive clones, which we now discuss in paragraph 4 of the first section of Results:

“We note that for all the treatment patients Pt4-6, a fitness landscape inversion occurs, such that minor clones present at early untreated stages become prevalent under treatment, identifying them as resistant clones in the treated samples. Resistant clones arise on different clades (i.e., branch of the phylogenetic tree) than clones in untreated samples ([Figure 2](https://docs.google.com/document/d/19YPI2Ch3hqwdPje_8lKfijphZY9fhh7B3mjD_z9R1oM/edit#fig_DLPtree)a), the latter exhibiting near neutral fitness as determined by a Wright-Fisher model [[13]](https://paperpile.com/c/SxU1B4/iM7DP). In Pt4, clone B, appears as a minor population in the untreated samples, evolves through minor gene dosage mutations into clone A, which exhibits a clonal sweep, becoming the resistant and only clone after two or more rounds of cisplatin treatment. In Pt5, clones B and C diminish in prevalence in the untreated samples, but their parent, clone D, evolves into clone A, a resistant clone under treatment, which is on a very different clade than the sensitive clone G that increases in abundance in UnRx. In Pt6, clone G, the parent of the sensitive clone E, expands in the treated samples, but the fittest resistant clone, clone H, is again on a very different clade than the sensitive clone E. Thus clones originating from different clades in the phylogenetic tree based on CNA mutations have different fitness under UnRx and Rx or RxH samples, as previously shown [[13]](https://paperpile.com/c/SxU1B4/iM7DP). “

In addition, the ancestral relationship between clones is discussed in various parts of the manuscript, such as, for example in the third paragraph of Results section 2:

“untreated high fitness clone H, which is in a distant branch to strongly platinum resistant clone A”.

2.4 Related, p9-line13 - the phrase "outside of clonal CNA differences" is very confusing to me, do the authors mean "in genomic regions with uniform copy number across all clones"?

Response: We mean in genomic regions that have the same copy number across the two clones that are compared. We have clarified this in paragraph 7 of Results, please see the response to Q2.2 above for the text insertion..

2.5 I couldn't see a definition of "canonical cis transcripts" (fig 2d)

Response: “Canonical” was a term we had previously used and omitted to update this term in Figure 3. In the Results section, we defined two types of in-cis genes: copy-number correlated in-cis transcripts (corresponding to ‘Gain Up’, ‘Loss Down’ canonical in-cis genes in Figure 3d), and copy-number anti-correlated in-cis transcripts (corresponding to ‘Gain Down, ‘Loss Up non canonical in-cis genes in Figure 3d). We have now corrected the terms in Figure 3, and changed from ‘canonical’ to ‘copy-number correlated’, and ‘non canonical’ to ‘copy-number anti-correlated’.

2.6 What is the definition of a "fitness gene"? Is more or less expression of these genes associated with increased fitness (and how is fitness measured?)

Response: Fitness genes in the description is the curated set of Pan Cancer core fitness genes that is provided from the published paper Behan et al, 2019, “Prioritization of cancer therapeutic targets using CRISPR–Cas9 screens”. In our study, we would like to see whether this list of core fitness genes is enriched in the regulated genes results from our analysis. To make the manuscript less confusing, we revised our text (see the last paragraph of “Copy number variation…” section in Results, and changed the phrase “differentially expressed core fitness genes” into “known pan cancer core gene set”.

In the reference paper, under “Defining core and context-specific fitness genes”, Behan et al noted that: “genes required for cell fitness in specific molecular or histological contexts are likely to encode favorable drug targets, because of a reduced likelihood of inducing toxic effects in healthy tissues”. Also, they note: “To identify core fitness genes, we developed a statistical method, the adaptive daisy model (ADaM), to adaptively determine the minimum number of dependent cell lines that are required for a gene to be classified as a core fitness gene. Genes that were defined as core fitness in at least 12 out of 13 cancer types were classified as pan-cancer core fitness genes”.

2.7 The dynamics of gene expression changes through treatment (end of p10 and top of p11) is interesting, though unfortunately I found the terminology challenging again. How is "stably expressed" defined? (I thought it must be assessed by looking at the same clone across time, but that seems to be a distinct analysis in a later paragraph). The authors also use the phrase "stable drug expression" but I presume that is a typo

Response: We have now added in parentheses what we mean by stable: “stable between treatment and holiday transcriptomes” now reads

“ stable (i.e., not differentially expressed, fdr > 0.1) between treatment and holiday transcriptomes”.

Also we have clarified “stable drug expression” by changing it to

“stable expression under treatment”.

2.8 The reports that chemo challenge tends to generally broadly switch on gene expression, and then expression decays towards pretreatment values, is very interesting and illustrates the plasticity of the gene expression response. Can the authors say anything about differential sensitivity of these "reverted" clones to subsequent rounds of treatment? Are they equally sensitive to treatment once the gene expression has returned to "baseline"? Functional experiments here would be wonderful (drug testing ex vivo) but I think are unreasonable to expect the authors to perform.

Response: We thank the reviewer for the very interesting comment and observation. In our drug treatment paradigm, treated tumors were exposed every three days to 2mg/Kg cisplatin up to a maximum of 8 doses and tumours harvested for analysis and re-transplant. From the day of re-transplant, there was an interval of approximately 4-6 weeks before the the next round of treatment was started to allow the re-transplanted tumours to establish and grow to a measurable size (see Methods, section “TNBC PDX timeseries treatment with cisplatin”). This cycling of treatment and treatment free intervals mirrors the way chemotherapy is delivered to patients. Thus the responses of the tumours after re-transplant are really after a drug free interval. The held out holiday samples allow us to establish the state of the transcriptome before treatment is re-initiated. This has been clarified in the methods

“In the re-transplant, all implanted mice were left without drug treatment for a period of 4-5 weeks until visible tumours formed and then half the mice were re-treated (Rx arm) and half left to grow further to endpoint - the latter harvested as the holiday interval (RxH) tumours. Although we did not sample gene expression immediately before treatment started (this would have been destructive to the tumours), the observed responses of clones in the treated mice reflects a drug-free period before treatment started, mirroring the way chemotherapy is delivered to patients. The holiday population (RxH) was without the drug for the same duration as the parallel mice treated with cisplatin for one cycle. RxH was subjected to one, two, or three cycles of cisplatin but entered a drug-free or holiday state in the corresponding passage, where they are compared with tumors continuously treated with cisplatin and those entirely treatment-naive. The RxH are parallel transplants that never again receive drugs and are allowed to grow, to establish the full reversion status of the transcriptome after the *prior* treatment.”

All the tumors when transplanted from previous treated passage, take 4-6 weeks to show up. We then randomly divide them into two groups-Treated (Rx) and treatment holiday (RxH). Rx-would be treated for another cycle of cisplatin while the RxH would not. They will continue to grow untreated till humane end point of the mouse.

2.9 Two cases (in patients 4&5) where a clone with distinct CNAs sweeps during treatment are highlighted, and in these cases expression doesn't revert to the pretreatment state. It wasn't clear to me whether or not the fixed change in expression was simply because of the gene dosages expect from the CNA, or if there were genome wide (trans?) differences in gene expression. Presumably the latter but could this be clarified?

Response: We agree that Pt4 expression at the last two time points did not revert back to baseline in comparison to Pt6, which reverted to more than 70% by the last time point. To confirm, we have added Supplementary Figure 11 panel c. To answer the in-cis and in-trans question, we have added NEW Supplementary Figure 11 panel a. The following text in Results section “Clonal and non-clonal…” and Supplementary Figure 11 were added or modified:

“In this case, few reversible genes were observed (only 15 genes at X6 and 19 genes at X7, all in-trans, whereas 2513 genes were found at X5, [Supplementary Table 7](https://docs.google.com/document/d/19YPI2Ch3hqwdPje_8lKfijphZY9fhh7B3mjD_z9R1oM/edit#sup_dynamic)), consistent with a fixed genomic landscape of drug resistance ([Figure 4](https://docs.google.com/document/d/19YPI2Ch3hqwdPje_8lKfijphZY9fhh7B3mjD_z9R1oM/edit#fig_dynamic)e-g), with minimal transcriptional plasticity ([Supplementary Figure 11](https://docs.google.com/document/d/19YPI2Ch3hqwdPje_8lKfijphZY9fhh7B3mjD_z9R1oM/edit#sfi_dynamic2)c). A similar observation was made for clone A of Pt5, where the transcriptional landscape is fixed at the last passage (X10). On the contrary, clonal sweeps of resistant clones were not detected in Pt6 (see also [Figure 2](https://docs.google.com/document/d/19YPI2Ch3hqwdPje_8lKfijphZY9fhh7B3mjD_z9R1oM/edit#fig_DLPtree)c), and similarly a large number of reversible genes were found at all time points (> 695 for Pt6, [Figure 4](https://docs.google.com/document/d/19YPI2Ch3hqwdPje_8lKfijphZY9fhh7B3mjD_z9R1oM/edit#fig_dynamic)e, >70% by the last passage, [Supplementary Figure 11](https://docs.google.com/document/d/19YPI2Ch3hqwdPje_8lKfijphZY9fhh7B3mjD_z9R1oM/edit#sfi_dynamic2)c)”

“Overall, the proportions of in-cis versus in-trans genes that contribute to the stable expression state of drug exposed cells (52-61% for Pt4, 20-45% for Pt5 and 0-12% for Pt6, [Figure 4](https://docs.google.com/document/d/19YPI2Ch3hqwdPje_8lKfijphZY9fhh7B3mjD_z9R1oM/edit#fig_dynamic)b, [Supplementary Figure 11](https://docs.google.com/document/d/19YPI2Ch3hqwdPje_8lKfijphZY9fhh7B3mjD_z9R1oM/edit#sfi_dynamic2)a) were generally higher than those of untreated passages ([Figure 3](https://docs.google.com/document/d/19YPI2Ch3hqwdPje_8lKfijphZY9fhh7B3mjD_z9R1oM/edit#fig_manhattan)d). ”

**Supplementary Figure 11: Dynamic gene comparisons, supplementary panels to Figure 4.**

(a) Percentages of in-cis versus in-trans genes for the genes that remain fixed after drug withdrawal, as in Figure 4b. “In-cis” includes all the “induced” and “repressed” genes in-cis, “In-trans” includes all the “induced” and “repressed” genes in trans. (b) Gene level comparisons for Pt4. Left panel: comparison of Rx and RxH clones against UnRx clone H, same as in Figure 4b left panel. Right panel: comparison against UnRx clone C. (b,c) X-axis: the same comparisons as in Figure 4b,e for Pt4-6. Y-axis: percentages. (c) Percentages for reverted versus unreverted genes for the same comparisons as in Figure 4b,e. Reverted genes include all the in-cis and trans genes in category “towards UnRx” in Figure 4e. Unreverted includes all the in-cis and trans genes that are “induced”, “repressed” and “away from UnRx” in Figure 4b,e.

2.10 The dynamics of reversion to a pre-treatment gene expression state are also interesting - and the notion of "transcriptional memory". Could the authors **speculate** on the mechanism, is it likely genetic or epigenetic? (or something else)

Response: In the discussion we raise the following points:

“Bivalent promoters are known for repressing the developmental genes in embryonic stem cells which gain expression during the differentiation for instructing lineage specification [[16]](https://paperpile.com/c/SxU1B4/kQ2I) via epigenetic mechanisms[[17]](https://paperpile.com/c/SxU1B4/NmyS). Interestingly, all the five gene modules achieved from pseudotime analysis in Pt6 were enriched in bivalent promoters (Supplementary Figure 14c), while comparatively only two and three modules were enriched in bivalent promoters for Pt4 and Pt5 respectively (Figure 6c,d). Therefore, we believe that the activation of the developmental/differentiation genes in the bivalent promoters enriched modules in Pt6 during its response to drug treatment instructed lineage specification to new state, hence Pt6 holiday passages did not revert back to drug-naïve transcriptional state when compared to the Pt4 and Pt5.”

2.11a I felt the pseudotime analysis sits uneasily with the earlier analysis, as in the pseudotime analysis temporal dynamics are inferred, but the data has actually directly measured changes over time and through evolution (and these analyses could detect dynamics, such as oscillations that would presumably not be detected in pseudotime analysis). I wonder if the manuscript (i.e. narrative) would be clearer without this section.

Response: We feel that we should keep the pseudotime analysis for the following important reasons.

Firstly, related studies in transcriptomic analysis [[18,19]](https://paperpile.com/c/SxU1B4/wJcU+I4CS) commonly combine the classical analysis (DE, pathway analysis) and pseudotime analysis because the latter allows us to strengthen the results from the former analysis. Here, in our study, with only a starting cell cluster from untreated cells at early passage as prior, Slingshot package can detect different lineages corresponding to untreated cells, treated, and drug holiday lineages matching with observations in the earlier analysis of drug responses and clonal prevalence. Moreover, obtained regulated gene modules from pseudotime analysis enrich similar pathways as in earlier pathway analysis from Figure 5. Hence, the pseudotime results confirm our finding in the earlier analyses.

Secondly, since the datasets in this study contain discrete time series data, we can directly measure the changes over time and through evolution, as we did in the first part of the results section. But this study mainly focuses on the most dominant clonal cells at each time point. However, within each specific time point, it is conceivable that there could be several sub-populations with different degrees of drug response. The next question is whether we can extend this study into more details about dynamic cell evolution, and dynamic gene regulation programs. Pseudotime analysis allows us to place cells into the global landscape and focus on the transition between cell states based on global transcriptional changes. From that, we can predict cell fates in response to drugs.

To clarify why we believe the pseudotime analysis is important, we added the texts to the Results section “Pseudotime analysis identifies…” of the manuscript, as follows:

“In summary, the pseudotime analysis allows us to strengthen the results from the earlier analyses. The untreated, treated, and drug holiday identified lineages match with the observations in the earlier analysis of drug response and clonal prevalence. Moreover, the regulated gene modules as a result of the pseudotime analysis enriched similar main pathways as in the earlier analyses. Pseudotime analysis allows to place cells into the global landscape and emphasize the transition between cell states based on global transcriptional changes. ”

2.11b It occurred to me that there is selection in the experiment to both the drug and the host mouse. The analysis addresses the first in detail, but can host selection be delineated too?

Response: Yes, the host selection can be delineated. In our earlier paper ([[13]](https://paperpile.com/c/SxU1B4/iM7DP) Extended fig.4-UnRx, different clones neutral fitness) in which we measured the relative fitness (1+s) of clones with a Wright-Fisher model, we mapped the fitness of untreated but passaged tumours and computed fitness. Many of these clones behaved close to neutrality, but for some clones a positive or negative fitness measure occurred. In these situations both the intrinsic clonal fitness and clonal fitness within the host environment are measured. Moreover, these fitness measures (clonal trajectories) were found to be reproducible in biological repeat samples and in deliberate re-mixing and re-transplant experiments where the population sizes were reset and the experiment re-run. The text clarifies this and now reads:

“We set out to measure copy number aberration (CNA) associated and copy number independent transcriptional variation in subpopulations of patient derived xenografts passaged over months to years under neutral (no drug intervention) conditions (UnRx) and with parallel cisplatin treatment (Rx) and cisplatin holiday (RxH) conditions ([Figure 1](https://docs.google.com/document/d/19YPI2Ch3hqwdPje_8lKfijphZY9fhh7B3mjD_z9R1oM/edit#fig_overview), Methods). We have previously measured CNA associated clonal fitness using a Wright Fisher model for treated and untreated passages and validated the measured fitness with repeated mixture re-transplant experiments [[13]](https://paperpile.com/c/SxU1B4/iM7DP)“

And

“We have previously measured the clonal fitness of Pt4,5,6 under different passaging conditions [[13]](https://paperpile.com/c/SxU1B4/iM7DP). Briefly, the fitness coefficient denotes the relative growth potential rate (increased or attenuated) of a given clone over time and was quantified using a Wright-Fisher inspired Bayesian probabilistic framework named *fitClone*. The fitness coefficient results from this previous analysis for patients Pt4-6 are included in [Supplementary Figure 3](https://docs.google.com/document/d/19YPI2Ch3hqwdPje_8lKfijphZY9fhh7B3mjD_z9R1oM/edit#sfi_pt4)b, [Supplementary Figure 4](https://docs.google.com/document/d/19YPI2Ch3hqwdPje_8lKfijphZY9fhh7B3mjD_z9R1oM/edit#sfi_pt5)b, [Supplementary Figure 5](https://docs.google.com/document/d/19YPI2Ch3hqwdPje_8lKfijphZY9fhh7B3mjD_z9R1oM/edit#sfi_pt6)b and [Supplementary Table 2](https://docs.google.com/document/d/19YPI2Ch3hqwdPje_8lKfijphZY9fhh7B3mjD_z9R1oM/edit#sup_clonecoeff). In addition to fitness quantification, we have also previously confirmed clonal fitness by experimental mixing and serial re-transplantation experiments [[13]](https://paperpile.com/c/SxU1B4/iM7DP). In the present study, clones from the treatment series with high fitness coefficient were labeled as *Rx (resistant to drug), and clones from the untreated series with high fitness coefficient were labeled as *UnRx (sensitive to drug, [Figure 2](https://docs.google.com/document/d/19YPI2Ch3hqwdPje_8lKfijphZY9fhh7B3mjD_z9R1oM/edit#fig_DLPtree)a, [Supplementary Table 2](https://docs.google.com/document/d/19YPI2Ch3hqwdPje_8lKfijphZY9fhh7B3mjD_z9R1oM/edit#sup_clonecoeff)). “

Minor points:

2.12 - What was the initial death rate upon drug treatment? (IC50 value, etc) Would be nice to know to understand the strength of selection in the experiment.

Response: The death rate is challenging to calculate because tumor growth is reduced, and this is a compound effect of cell division rate and death rate. The number of cycles required for 50% tumor growth inhibition (TGI) in early untreated passages was determined initially, before initiating serial treatments. We conducted a mini screen of a few tumors and selected these three patients, as the TGI for cisplatin was more than 60%. The goal of each treatment cycle was to continue treating the tumor with 2 mg/kg of mouse weight for a maximum of eight cycles until the tumor shrinks by 50%. We observed that in the first cycle, the tumor shrank up to 50% after three doses but gradually increased as the tumor became more resistant, as mentioned in [[13]](https://paperpile.com/c/SxU1B4/iM7DP). Now we have added an additional panel, b, in Figure 1. (b) Tumour growth inhibition graphs for Pt4, Pt5 and Pt6.

The strength of selection in these experiments are already published in our previous paper by measuring with Wright-Fisher modeling of fitness [[13]](https://paperpile.com/c/SxU1B4/iM7DP). Now we have explained the naming convention that matches our previous paper in the method section under “TNBC PDX timeseries treatment with cisplatin**”:**

**“**For patient naming, Pt1, Pt2, Pt3 in this study correspond to the breast cancer patient-derived xenograft SA501, SA530, SA604 [[20]](https://paperpile.com/c/SxU1B4/lt8EC), and Pt4, Pt5, Pt6 in this study correspond to SA609, SA535, and SA1035 respectively in our previous study [[13]](https://paperpile.com/c/SxU1B4/iM7DP)**”.**

2.13 - The abstract states the paper contains 126,566 single cell transcriptomes, but only 55,025 survived QC. The smaller number feels fairer to report.

Response: we agree, and we now report only the post-filter number in the abstract and the first paragraph of Results.

2.14 - What's the time points in a timeline? Passage numbers are given, but absolute time would nice to know.

Response: The growth curves that contain the details of tumour volume (mm3) and days from palpable tumour to collection are noted in Extended Data Fig. 3 of our previous study (Salehi et al, 2021, “Clonal fitness inferred from time-series modelling of single-cell cancer genomes”), please find a copy of this figure below.

To make it clear, we now included in Methods, under section “TNBC PDX timeseries treatment with cisplatin”, the following text:

“For patient naming, Pt1, Pt2, Pt3 in this study correspond to the breast cancer patient-derived xenograft SA501, SA530, SA604 [[20]](https://paperpile.com/c/SxU1B4/lt8EC), and Pt4, Pt5, Pt6 in this study correspond to SA609, SA535, and SA1035 respectively in our previous study [[13]](https://paperpile.com/c/SxU1B4/iM7DP), see also Table 1 and [Supplementary Table 1](https://docs.google.com/document/d/19YPI2Ch3hqwdPje_8lKfijphZY9fhh7B3mjD_z9R1oM/edit#sup_ncells). The growth curves that contain the details of tumour volume (mm3) and days from palpable tumour to collection are noted in Extended Data Fig. 3 of our previous study [18]”.

We didn’t add the figure in the manuscript, but posted it below:

Copied figure from our previously published paper.

2.15 - Were the PDX performed in replicates? If there are some replicates the repeatability of evolution would be great to know.

Response: This was established with multiple forms of replication, including (i) replicate mice (biological replicates), (ii) replicated lineages and (iii) repeated experiments in which tumour populations were re-mixed in different proportions and the entire lineage selection-transplant experiment repeated over multiple passages, to establish rigorously whether the fitness measurements of copy number clones by Wright-Fisher, were correct. This is described in detail in Salehi et al, however to make this more accessible to the readers of this paper, we have added a new section in Methods about the forms of replication.

“**Replicate Lineages of Treatment, Holiday, and No Treatment**

Replication included: (i) biological replicate transplants per time point; (ii) lineage replicates, where transplants were serially transplanted in parallel; (iii) re-mixing and re-transplant of tumour populations of different putative fitness at different population fractions, with a re-run of serial treatment or passaging, to revalidate the clone dynamics in each situation. These details are reported in our previous paper [[13]](https://paperpile.com/c/SxU1B4/iM7DP). We conducted parallel analyses of untreated lineage and counter drug holiday samples for that time point.”

Therefore, we demonstrated that resistant clones in both series share a common precursor, as previously published [[13]](https://paperpile.com/c/SxU1B4/iM7DP); also refer to the figure below). We conducted experiments where fitness was re-validated by re-mixing clones at different proportions from initial and late passages of time series. We re-ran the time series by serial transplantations to confirm clonal dynamics and same lineages taking over in later passages.

**Biological Replicates:** (a) Schematics of Pt4 showing the replicate treated series generated in Pt4 (b) Schematics of mixing experiments.

—-----------------------------------------------------------------------------------------------------

# Reviewer #3:

Kabeer et al. presented a study of transcriptional drug responses using PDX models of TNBC. While this study proposed an interesting single cell re programing model, the data support is relative weak, particularly, the only three treated PDX Pt had completely different clonality. The analysis of in-cis and in-trans- dosage effects is shallow. The study could be much improved through more in-depth analyses.

We thank the reviewer for helpful suggestions on this point and have added additional analysis as described below and in response to reviewers 1 and 2.

3.1 In the alignment of DLP and scRNAseq data, there exist large portions of unassigned cells, indicating sampling bias of the two approaches and or alignment errors. Could the author explore more?

Response: In our first submission the apparently large fraction of unassigned cells present in some clones, was not due to sampling bias between approaches, nor alignment errors. It was due to clonealign thresholds chosen to be stringent in mapping transcriptomes to genotypes, the fraction of genome altered, and a lower count for some samples. For the four samples affected (Pt3 X7, Pt6 UnRx X4 and X5 and Pt6 Rx X5), we have now rerun clonealign with less stringent thresholds (see Supplementary Table 3), and recomputed Supplementary Figures 2 and 5, Figure 2e, Figure 5 (column Pt6 X5), added one column to Table 1, and added several columns in Supplementary Table 1 to show the number and proportions of cells remaining or excluded at every filtering or assignment step.

In detail, the assignment of scRNA-seq single cells to clones is undertaken using a probabilistic model, clonealign. Before assignment, however, clonealign selects the genes that have any copy number difference in any clone, and then removes the cells that have total counts in the selected genes less than a threshold (we used min_counts_per_cell=100 for all samples). This threshold is intended to ensure that only high gene count cells are included in each clone transcriptome.

In the first submission, overall, 90.6% of the total number of filtered cells were kept by the clonealign filters, but there were four notable outliers, all at early passages: Pt3 X7, Pt6 UnRx X4 and X5 and Pt6 Rx X5, where only 22%, 32%, 41% and 43% of the filtered cells were kept, respectively. For all of these four samples, the number of selected genes and the overall counts are smaller than for the other samples, therefore the min_counts_per_cell threshold of 100 was too stringent. We have now used thresholds of 25 for Pt6 UnRx X4 and 50 for the remaining three samples, in order to be able to retain more cells (see Supplementary Table 3). Now, clonealign uses on average 94.4% of the previously filtered cells, and all samples use at least 56% of the filtered cells.

After the clonealign cell filter, the vast majority of the cells are assigned to clones, with average 2.7% cells unassigned, maximum 18.7%. It is interesting to see that with more cells used as input clonealign also labeled more cells as “unassigned” (at the first submission the average percentage of unassigned cells was 1%, maximum 9%). This is reassuring, since some of the input cells included lower quality samples (due to the less stringent threshold).

We note that using a less stringent filter for the four samples mentioned above did not significantly change the results. First of all, the 4 samples were from very early time points, and the vast majority of our analysis was done at later time points. Very minor changes are noted at Figure 2e, where the Pearson correlation coefficients are now lower by only 0.03 and 0.02 for Pt3 and Pt6, respectively. In Figure 5 (pathway analysis), where the Pt6 X5 comparison is now between Rx clone B (instead of D; B is the most abundant clone in the DLP clones, so this is a better clone to explore) and UnRx clone A, a few more pathways are observed than with higher threshold - and this is in fact more consistent with the pathways at X6, so there is more convergence. No other results were influenced by the looser threshold.

3.2 The clonal compositions of three treated patients were very different. What are the underlying factors, particularly, Pt4 had one dominant clone after treatment while the other two had many co-existing clones?

Response: We deliberately chose three PDX lines that represent the spectrum of genomic instability in TNBC populations, ranging from highly unstable TNBC tumours with many CNA mutations per cell, to simpler tumours with only 2 or 3 genotypes. This reflects the distribution in patients as we and others have previously described [[3,21–23]](https://paperpile.com/c/SxU1B4/XroZ+qFFT+RAEP+XXKQ).

Moreover, the clonal fitness patterns to platinum also represent the spectrum of responses. Pt4 has a strong/dominant intrinsic clonal resistance, i.e. has a single clone that is much fitter to platinum and this fitness landscape is peaked in a single clone, whereas Pt5 and Pt66 exhibit differing degrees of clonal fitness with more than one clone exhibiting degrees of resistance. In our earlier paper we showed a fitness landscape inversion with all three PDX lines, but pt4 has the strongest intrinsic resistance in a single clone. Thus in pt4 there is a rapid clonal sweep with platinum, whereas Pt5,6 show a slower and more distributed clonal response. These choices were deliberate, to exemplify the differences between strong genomically determined resistance and situations where genomic and non-genomic factors may be more balanced.

The exact molecular factors underpinning the differences in genomic instability are unknown, however it is notable that the lines exhibit different intrinsic DNA repair signatures. We have clarified these points under the Results section “Clonal and non-clonal transcriptional responses to therapy reveal intratumoural heterogeneity”, as follows:

“While Pt4 and Pt5 displayed more up-regulated pathways than down-regulated pathways, especially after several rounds of treatment, Pt6 showed the opposite trend in the last two time points, showing heterogeneity in transcriptional responses across patients. This is consistent with previous work [[13]](https://paperpile.com/c/SxU1B4/iM7DP), and could be because of different DNA repair deficiency phenotypes of Pt4 and Pt5 as BRCA2 and BRCA1 types respectively, while Pt6 [[24]](https://paperpile.com/c/SxU1B4/PBqT4) was exhibiting APOBEC-BRCA1 germline mutational signatures. Interestingly, different clones of the same sample may encode common as well as individual pathways, as observed for example in Pt5 X10, where holiday clones A and E display four common pathways and ten individual pathways, when compared against the corresponding untreated sample ([Figure 5](https://docs.google.com/document/d/19YPI2Ch3hqwdPje_8lKfijphZY9fhh7B3mjD_z9R1oM/edit#fig_path)a).”

3.3 The untreated samples also showed one-directional clonal enrichment in all three patients. The authors compared them to Rx enriched clones. However, are there shared features among them? Are they due to random drift?.

??

They are not due to “drift”, as noted in responses above. We realize this was not well described in the current text, for brevity and was thus not apparent. We addressed precisely this question, as to whether the dynamics had a likelihood consistent with drift versus non-neutral in this transplant series in an earlier paper, Salehi et al, Nature 2021. Briefly, we showed in our previous CNA fitness paper Salehi et al, 2021 [[13]](https://paperpile.com/c/SxU1B4/iM7DP) with a modified Wright-Fisher (WF) diffusion population genetics model that the changes in population prevalence of clones under treatment do not fit a drift situation, i.e, neutral fitness coefficients. Moreover, we validated these WF fitness measurements of copy number defined clones, by conducting experiments where the clonal population proportions were reset and the entire experiment re-run, serially transplanting +/- treatment, with the same outcome and fitness determinations. Thus we conducted experiments in which fitness was re-validated by remixing clones at different proportions from initial and late passages of time series tumors. Those samples were rerun, similar to the untreated time series, through serial transplantations to confirm clonal dynamics, and we found that the same lineages took over in later passages.

Results from this study (Fig. 3a, Extended Fig. 4, Extended Fig. 8) showed that three patients Pt4-Pt6 exhibited evidence of clonal dynamics and variation in selection coefficients consistent with positive selection and differential fitness. Some clones propagated after the initial time point, exhibited expansion from minor prevalence to the high prevalence at the late time points with highest selection coefficients and formed a distinct clade distinguished by mutation events compared to untreated clones.

In each series, emergent clones following treatment were distinct in phylogenetic origin from those with high fitness in the untreated setting, indicating an inversion of the clone fitness landscapes (Fig. 4a, Extended Data Fig. 8a Salehi et al 2021). Suppression of high-fitness clones that dominated in the absence of treatment and expansion of low-fitness and/or previously unobserved genotypes led to a substantially altered rank order of selection coefficients in treated samples relative to untreated samples (Fig. 4b, Extended Data Fig. 8 b, c).

In summary, the difference between treated versus untreated clones is statistically more likely to be explained by fitness differences between copy number genotypes, rather than random population fluctuations under a “drift” or neutral fitness model. Moreover, in the previous study, we observed the reproducibility of these clones in replicate transplants that strengthen the evidence of genotype difference between fitted clones in each treated/ untreated direction.

We have added a brief description of the above in two paragraphs in the first section of Results:

“We set out to measure copy number aberration (CNA) associated and copy number independent transcriptional variation in subpopulations of patient derived xenografts passaged over months to years under neutral (no drug intervention) conditions (UnRx) and with parallel cisplatin treatment (Rx) and cisplatin holiday (RxH) conditions ([Figure 1](https://docs.google.com/document/d/19YPI2Ch3hqwdPje_8lKfijphZY9fhh7B3mjD_z9R1oM/edit#fig_overview), Methods). We have previously measured CNA associated clonal fitness using a Wright Fisher model for treated and untreated passages and validated the measured fitness with repeated mixture re-transplant experiments [[13]](https://paperpile.com/c/SxU1B4/iM7DP)“

And

“We have previously measured the clonal fitness of Pt4,5,6 under different passaging conditions [[13]](https://paperpile.com/c/SxU1B4/iM7DP). Briefly, the fitness coefficient denotes the relative growth potential rate (increased or attenuated) of a given clone over time and was quantified using a Wright-Fisher inspired Bayesian probabilistic framework named fitClone. The fitness coefficient results from this previous analysis for patients Pt4-6 are included in [Supplementary Figure 3](https://docs.google.com/document/d/19YPI2Ch3hqwdPje_8lKfijphZY9fhh7B3mjD_z9R1oM/edit#sfi_pt4)b, [Supplementary Figure 4](https://docs.google.com/document/d/19YPI2Ch3hqwdPje_8lKfijphZY9fhh7B3mjD_z9R1oM/edit#sfi_pt5)b, [Supplementary Figure 5](https://docs.google.com/document/d/19YPI2Ch3hqwdPje_8lKfijphZY9fhh7B3mjD_z9R1oM/edit#sfi_pt6)b and [Supplementary Table 2](https://docs.google.com/document/d/19YPI2Ch3hqwdPje_8lKfijphZY9fhh7B3mjD_z9R1oM/edit#sup_clonecoeff). In addition to fitness quantification, we have also previously confirmed clonal fitness by experimental mixing and serial re-transplantation experiments [[13]](https://paperpile.com/c/SxU1B4/iM7DP). In the present study, clones from the treatment series with high fitness coefficient were labeled as *Rx (resistant to drug), and clones from the untreated series with high fitness coefficient were labeled as *UnRx (sensitive to drug, [Figure 2](https://docs.google.com/document/d/19YPI2Ch3hqwdPje_8lKfijphZY9fhh7B3mjD_z9R1oM/edit#fig_DLPtree)a, [Supplementary Table 2](https://docs.google.com/document/d/19YPI2Ch3hqwdPje_8lKfijphZY9fhh7B3mjD_z9R1oM/edit#sup_clonecoeff)). “

3.3.1 “However, are there shared features among them?

Response: With respect to **shared features** we were slightly confused by the question. There is not unidirectional expansion of untreated clones in the treated case, rather there is an inversion of the fitness landscape - clones that rise to high prevalence under weak positive or near neutral fitness in untreated cases, end up being diminished or eradicated completely, under treatment. There will be many regions of similarity between these clones because they share a lineage history so in fact the majority of the genome will be in common. The regions of *difference*, which define the clonal genotypes are of interest because potentially they encode gene dosage associated transcriptional differences, which is the focus of the current manuscript as a proof of principle. The pathway convergence analysis of Figure 5 emphasizes this as the comparisons across clones reflect differences in differential gene expression with respect to untreated clones. To better address pathway convergence, in the new Supplementary Figure 12 (see below) in response to a subsequent question we make these differences between treatment and holiday situations more explicit by computing the pathway similarities and their in-cis, in-trans contributions clone-wise (previously summarized only) to further exemplify.

3.4 Fig 2e figure legends didn't match with axis labels, and both were poorly stated. The r values labeled in figure were not clearly explained. It's surprisingly high that gene expression and copy numbers had up to 93% concordance.

Response: The concordance is between **the clone proportions**, not between the gene expressions and copy numbers. We have now updated the legend of Figure 2e, as follows:

“Each point shows the clonealign-inferred scRNA-seq clonal proportion (y-axis) versus the corresponding DLP+ clonal proportion (x-axis) for each clone in each sample. Each patient is denoted by a different shape. Pearson correlation coefficients for each patient are reported at the top.“

3.5 It isn't clear if copy numbers had strong dosage effects in treatment response genes. The in-cis transcript accounts for 5.1-57.4% of DEG across selected clones in three treated PDX-Pt 4,5,6. What are the in-cis percentages of any clone pairs in un-treated PDX samples? What are the in-trans transcript percentages in treated group? Overall, what are the percentages of copy number in-cis transcripts in treated groups comparing to un-treated groups? Would dosage effect stronger or weaker in expanded clones compared to other shrinking clones?

Response: We thank the reviewer for this thorough comment. In the first submission, we quantified the in-cis/ in-trans percentages for treated groups from Pt4-6 in Figure 3d, and for untreated groups from Pt1-3 in Supplementary Figure 8c.

To address this question, we conducted additional DE analyses on the 3 treated patients Pt4-6 and reported the results in Supplementary Figure 9. We have also added the following text in Results, section “Copy number variation shapes PDX single cell transcriptomes…”:

“To understand the dosage effect of copy number in treatment response genes compared to untreated lines, we examined further the following classes of DE genes in-cis and in-trans categories: untreated comparisons: (i) UnRx vs. UnRx, and treated comparisons including: (ii) Rx expanding clones vs. UnRx, (iii) Rx shrinking clones vs. UnRx, (iv) RxH vs. UnRx ([Supplementary Figure 9](https://docs.google.com/document/d/19YPI2Ch3hqwdPje_8lKfijphZY9fhh7B3mjD_z9R1oM/edit#sfi_summary_cistrans)). Overall, the in-cis proportion of the treated comparisons are lower than in untreated comparisons in Pt5, Pt6 (mean+-sd = 37.55+-3.47 for treated and 58.52+-3.09 for untreated in Pt5, and 10.93+-3.63 for treated vs. 31.08+-5.02 for untreated in Pt6), showing that the majority of DE gene regulation in untreated clones associates with in-cis genes, ie regions of copy number difference. However, in contrast, in Pt4 serial transplants, in-cis genes contribute less in treated versus untreated comparisons (mean+- sd = 53.57+-1.12 for treated and 41.84+-14.1 for untreated). This difference across patients may be explained by the dosage effects in Pt4 due to gene dosage mutations conferring very strong fitness to platinum treatment ([Figure 2](https://docs.google.com/document/d/19YPI2Ch3hqwdPje_8lKfijphZY9fhh7B3mjD_z9R1oM/edit#fig_DLPtree)b).”

To answer whether dosage effects exhibit stronger or weaker effect in expanded clones compared to shrinking clones, we conducted the analysis on the 3 treated patients Pt4-Pt6, by calculating DE genes followed by reporting the percentage of CNA genotype dependent/independent in-cis, trans genes in the following categories: (ii) Rx expanding clones vs UnRx, (iii) Rx shrinking clones vs UnRx (Supplementary Fig. 9).

Overall, the Rx expand clones have slightly higher variance compared to Rx shrink clones in both in-cis, and in-trans proportions for all three patients demonstrating that Rx expand clones have stronger regulated events/ fitness under drug selection. In-cis genes contribution: (mean and sd are 53.55+-1.56 in ii compared to 53.33 in iii for Pt4, 37.27+-3.13 in ii compared to 34.26+-0.85 in iii for Pt5, 7.05+-1.9 in ii compared to 13.71+-2.73 in iii for Pt6). In-trans genes contribution: (mean and sd are 43.37+-1.76 in ii compared to 44.25 in iii for Pt4, 60.01+-3.48 in ii compared to 62.97+-0.37 in iii for Pt5, 90.21+-1.85 in ii compared to 83.92+-2.97 in iii for Pt6)

But these values do not point to a strong conclusion about dosage effect when comparing Rx expanded clones versus Rx shrinking clones. Moreover, looking back into phylogenetic trees of Pt4-Pt6, the expanding/shrinking clones are located in the distinct clades from untreated clones. So we expect both of them to exhibit greater divergence compared to untreated clones. So we do not have a firm basis with which to compare the dosage effect of Rx expanding clones versus Rx shrinking clones.

**Supplementary Figure 9:** Summary of in-cis and trans genes across four categories of differential expression comparisons, supplementary to Figure 3. in-cis(top row) and in-trans(bottom row) genes proportions in Pt4-6 for each type of comparisons. X-axis differentially expressed analyses are classified into 4 groups of comparisons: Rx expanding clones, Rx shrinking clones, RxH and UnRx, all versus UnRx clones. Red lines show medians for each group.

3.6 Could anti-correlated transcripts arise from other regulations, such as epigenetics in addition to copy number miscalling?

Response: Yes, there could be other regulations or mechanisms involved. In the discussion we have added the following points:

“The anti-correlated transcripts we identified could arise through multiple mechanisms, for example, some genes gaining copy gains could get down-regulated due to copy gains of other repressor transcription factors or imbalances in the gene regulatory networks that regulate its expression; genes that maintain a fixed level of gene product due to network interactions; some genes dosage alterations resulting in altered epigenetic marks leading to the counter-regulation etc.”

3.7 "We asked which cellular functions are represented by the cis- and trans- encoded transcription dynamics to drug exposure and withdraw". However, the whole paragraph didn't provide results regarding which genes are in-cis- and in-trans-encoded by copy number events. For instance, it will be interesting to learn if copy number changes converge to EMT related phenotypes (more cis-regulated than trans-regulated).

Response: In Figure 4b there is an omnibus calculation in-cis/in-trans for all pathways. We have now added Supplementary Figure 12 (see below), which shows the in-cis and in-trans proportions for all the pathway-specific differentially expressed genes at the latest time points for Pt4-6. We have also added Supplementary Table 9, which lists all the in-cis and trans pathway genes in all the comparisons in Figure 5.

Additionally, we have added the following in the main text:

“The in-cis and in-trans composition of the genes in all the hallmark pathways taken together for each comparison, was consistent with the proportions discussed earlier in [Figure 3](https://docs.google.com/document/d/19YPI2Ch3hqwdPje_8lKfijphZY9fhh7B3mjD_z9R1oM/edit#fig_manhattan) (mean percentage of in-cis genes 33.4%, 19.4% and 3.7% for Pt4,5,6, respectively, [Figure 5](https://docs.google.com/document/d/19YPI2Ch3hqwdPje_8lKfijphZY9fhh7B3mjD_z9R1oM/edit#fig_path)b). In-cis gene mean proportions for each individual pathway are also similar (35.1%, 17.2% and 4.6% for Pt4,5,6, respectively, [Supplementary Figure 12](https://docs.google.com/document/d/19YPI2Ch3hqwdPje_8lKfijphZY9fhh7B3mjD_z9R1oM/edit#sfi_pathways_cistrans)), with a few notable exceptions. Notably, the KRAS Signaling Down pathway, which contains 62.5% of the in-cis genes from Pt4, and MYC Targets V2, having 10.5% of the in-cis genes from Pt4 (see [Supplementary Table 9](https://docs.google.com/document/d/19YPI2Ch3hqwdPje_8lKfijphZY9fhh7B3mjD_z9R1oM/edit#sup_pathway_genes) for the list of all in-cis and in-trans pathway genes), both exhibit an excess of in-cis transcripts. All pathways at the last time points contained both in-cis and in-trans genes, except for five Pt6 pathways, including TNFA Signaling Via NFKB and Interferon Alpha and Gamma Response pathways, which contained only in-trans genes.”

**Supplementary Figure 12: Cis and trans gene proportions for the genes in the pathways at the latest time points for Pt4, Pt5 and Pt6, supplementary to Figure 5.** Left side: significantly enriched pathways (p < 0.05, vertical axis) from a ranked gene set enrichment analysis (GSEA) [[25]](https://paperpile.com/c/SxU1B4/WVqsA), using the Hallmark gene set collection from MSigDB [[25,26]](https://paperpile.com/c/SxU1B4/fqZ83+WVqsA), for Rx vs. UnRx and RxH vs. UnRx comparisons at the latest time points, same as in Figure 5. Right side: for each enriched pathway, showing the in-cis (orange) and in-trans (blue) proportions of all the differentially expressed genes (FDR < 0.01 and |log2 fold change| >= 0.25) that are part of the corresponding pathway. The red-contoured boxes represent cases where all the genes are in-trans and there are no in-cis genes.

3.8 How about the alignment between pseudo-time lineages and CNV lineages?

In the first submission, we characterized each lineage based on fraction of cells, inferred cell clonal labels, treatment conditions, and treatment time point (see Supplementary Figure 13a, 13b of revised manuscript). Beside that, the mapping between pseudotime lineages and gene dosage effect (CNA genotype dependent in-cis genes/ independent trans genes) were shown in Figure 6c, 6d, Supplementary Figure 14c. A gene is classified as in-cis, or in-trans gene types depending on the list of inferred clones and the copy number change between clones in each lineage.

To make it clearer, and highlight the alignment between pseudotime lineages and CNV lineages, we have added some additional analyses into Supplementary Figure 13:

The inferred clonal labels of scRNA-seq cells - results of clonealign are embedded in the cells UMAP projection to highlight the cell clonal landscape within the global cell landscape and along each lineage (Supplementary Figure 13c, 13d, 13e). Moreover, we included the cell clonal prevalences of copy number analysis at genomic level to emphasize the list of expanding, shrinking clones under different treatment conditions. From that, it is easier to see the mapping between each lineage and CNV clonal dominance. We have added the following description into the main text under the pseudotime analysis results section:

“The mapping between pseudotime lineages and clonal landscape was highlighted in [Supplementary Figure 13](https://docs.google.com/document/d/19YPI2Ch3hqwdPje_8lKfijphZY9fhh7B3mjD_z9R1oM/edit#sfi_traj45)c,d,e where resistant clones or drug holiday clones are present near the end points of treated, and holiday lineages, and early, shrinking cell clones are detected near the beginning of each lineage.”

The proportion of in-cis/ in-trans genes across each pseudotime lineage (from Figure 6c, 6d, Supplementary Figure 14c) are now summarized in Supplementary Figure 13f. From these results, we can clearly see the contribution of in-cis genes in treated lineages is higher than in untreated lineages for Pt4, Pt5, demonstrating that genes are strongly regulated under drug treatment lineages compared to untreated cells lineage. We have added the following description into the main text under the pseudotime analysis results section:

“In addition, we characterized each tradeSeq significant regulated gene as in-cis or in-trans depending on the inferred cell clonal labels and the copy number change between clones in each lineage ([Figure 6](https://docs.google.com/document/d/19YPI2Ch3hqwdPje_8lKfijphZY9fhh7B3mjD_z9R1oM/edit#fig_traj)c,d, [Supplementary Figure 14](https://docs.google.com/document/d/19YPI2Ch3hqwdPje_8lKfijphZY9fhh7B3mjD_z9R1oM/edit#sfi_traj6)c). We observed that the number of in-cis genes in treated lineages are higher than in untreated lineage for Pt4, Pt5 ([Supplementary Figure 13](https://docs.google.com/document/d/19YPI2Ch3hqwdPje_8lKfijphZY9fhh7B3mjD_z9R1oM/edit#sfi_traj45)f), demonstrating that genes are strongly dynamic under drug treatment lineages compared to untreated lineages (Pt4: untreated lineage L1: 58% in-cis genes compared to 66% in treated lineages L2, L3. Pt5: untreated lineage L1: 56% in-cis genes compared to 61% in treated lineages L2, L3, L4). In Pt6, in-cis genes contributions are similar (9%), and lesser than in-trans genes (91%) across lineages. This result is consistent with earlier analysis with the observation that most regulated gene types in Pt6 are in-trans. ”

Supplementary Figure 13: Characterizing each lineage based on the fraction of cells, fitted clone labels, and treatment conditions.

Minor:

3.91 Fig 1a - there wasn't annotation of light blue circle

We have added the annotations for light blue, yellow circles in Figure 1a of revised manuscript.

3.92. All gene names need to italic

Response: We have changed all the gene names to italic in the text.

3.93. Many words in sub-panels can be moved to figure legends.

Response: We have removed words from Figure 4 to make the figure less busy.

**References in response to comments.**

[1. Sui Q, Hu Z, Jin X, Bian Y, Liang J, Zhang H, et al. The genomic signature of resistance to platinum-containing neoadjuvant therapy based on single-cell data. Cell Biosci. 2023;13:103.](http://paperpile.com/b/SxU1B4/3KVP)

[2. Navin N, Kendall J, Troge J, Andrews P, Rodgers L, McIndoo J, et al. Tumour evolution inferred by single-cell sequencing. Nature. 2011;472:90–4.](http://paperpile.com/b/SxU1B4/QJDV)

[3. Shah SP, Roth A, Goya R, Oloumi A, Ha G, Zhao Y, et al. The clonal and mutational evolution spectrum of primary triple-negative breast cancers. Nature. 2012;486:395–9.](http://paperpile.com/b/SxU1B4/qFFT)

[4. Wang Y, Waters J, Leung ML, Unruh A, Roh W, Shi X, et al. Clonal evolution in breast cancer revealed by single nucleus genome sequencing. Nature. 2014;512:155–60.](http://paperpile.com/b/SxU1B4/3HX8)

[5. Lohr JG, Stojanov P, Carter SL, Cruz-Gordillo P, Lawrence MS, Auclair D, et al. Widespread genetic heterogeneity in multiple myeloma: implications for targeted therapy. Cancer Cell. 2014;25:91–101.](http://paperpile.com/b/SxU1B4/e9u2)

[6. Piscuoglio S, Ng CKY, Geyer FC, Burke KA, Cowell CF, Martelotto LG, et al. Genomic and transcriptomic heterogeneity in metaplastic carcinomas of the breast. NPJ Breast Cancer. 2017;3:48.](http://paperpile.com/b/SxU1B4/at0n)

[7. Liu Y, Xun Z, Ma K, Liang S, Li X, Zhou S, et al. Identification of a tumour immune barrier in the HCC microenvironment that determines the efficacy of immunotherapy. J Hepatol. 2023;78:770–82.](http://paperpile.com/b/SxU1B4/wbpP)

[8. Meyer M, Reimand J, Lan X, Head R, Zhu X, Kushida M, et al. Single cell-derived clonal analysis of human glioblastoma links functional and genomic heterogeneity. Proc Natl Acad Sci U S A. 2015;112:851–6.](http://paperpile.com/b/SxU1B4/mEO4)

[9. Patel BB, Ackerstaff E, Serganova IS, Kerrigan JE, Blasberg RG, Koutcher JA, et al. Tumor stroma interaction is mediated by monocarboxylate metabolism. Exp Cell Res. 2017;352:20–33.](http://paperpile.com/b/SxU1B4/DPRe)

[10. Zhang J, Song C, Tian Y, Yang X. Single-Cell RNA Sequencing in Lung Cancer: Revealing Phenotype Shaping of Stromal Cells in the Microenvironment. Front Immunol. 2021;12:802080.](http://paperpile.com/b/SxU1B4/DUjM)

[11. Tian Y, Li Q, Yang Z, Zhang S, Xu J, Wang Z, et al. Single-cell transcriptomic profiling reveals the tumor heterogeneity of small-cell lung cancer. Signal Transduct Target Ther. 2022;7:346.](http://paperpile.com/b/SxU1B4/SPLty)

[12. Kim C, Gao R, Sei E, Brandt R, Hartman J, Hatschek T, et al. Chemoresistance Evolution in Triple-Negative Breast Cancer Delineated by Single-Cell Sequencing. Cell. 2018;173:879–93.e13.](http://paperpile.com/b/SxU1B4/CcLNW)

[13. Salehi S, Kabeer F, Ceglia N, Andronescu M, Williams MJ, Campbell KR, et al. Clonal fitness inferred from time-series modelling of single-cell cancer genomes. Nature. 2021;595:585–90.](http://paperpile.com/b/SxU1B4/iM7DP)

[14. Laks E, McPherson A, Zahn H, Lai D, Steif A, Brimhall J, et al. Clonal Decomposition and DNA Replication States Defined by Scaled Single-Cell Genome Sequencing. Cell. 2019;179:1207–21.e22.](http://paperpile.com/b/SxU1B4/0ZC0K)

[15. Lai, Shah. HMMcopy: copy number prediction with correction for GC and mappability bias for HTS data. R package version.](http://paperpile.com/b/SxU1B4/iFqOw)

[16. Yu Y, Li X, Jiao R, Lu Y, Jiang X, Li X. H3K27me3-H3K4me1 transition at bivalent promoters instructs lineage specification in development. Cell Biosci. 2023;13:66.](http://paperpile.com/b/SxU1B4/kQ2I)

[17. Verma N, Pan H, Doré LC, Shukla A, Li QV, Pelham-Webb B, et al. TET proteins safeguard bivalent promoters from de novo methylation in human embryonic stem cells. Nat Genet. 2018;50:83–95.](http://paperpile.com/b/SxU1B4/NmyS)

[18. Ionkina AA, Balderrama-Gutierrez G, Ibanez KJ, Phan SHD, Cortez AN, Mortazavi A, et al. Transcriptome analysis of heterogeneity in mouse model of metastatic breast cancer. Breast Cancer Res. 2021;23:93.](http://paperpile.com/b/SxU1B4/wJcU)

[19. Kagohara LT, Zamuner F, Davis-Marcisak EF, Sharma G, Considine M, Allen J, et al. Integrated single-cell and bulk gene expression and ATAC-seq reveals heterogeneity and early changes in pathways associated with resistance to cetuximab in HNSCC-sensitive cell lines. Br J Cancer. 2020;123:101–13.](http://paperpile.com/b/SxU1B4/I4CS)

[20. Funnell T, O’Flanagan CH, Williams MJ, McPherson A, McKinney S, Kabeer F, et al. Single-cell genomic variation induced by mutational processes in cancer. Nature. 2022;612:106–15.](http://paperpile.com/b/SxU1B4/lt8EC)

[21. Nik-Zainal S, Davies H, Staaf J, Ramakrishna M, Glodzik D, Zou X, et al. Landscape of somatic mutations in 560 breast cancer whole-genome sequences. Nature. 2016;534:47–54.](http://paperpile.com/b/SxU1B4/XroZ)

[22. Curtis C, Shah SP, Chin S-F, Turashvili G, Rueda OM, Dunning MJ, et al. The genomic and transcriptomic architecture of 2,000 breast tumours reveals novel subgroups. Nature. 2012;486:346–52.](http://paperpile.com/b/SxU1B4/RAEP)

[23. Weisman PS, Ng CKY, Brogi E, Eisenberg RE, Won HH, Piscuoglio S, et al. Genetic alterations of triple negative breast cancer by targeted next-generation sequencing and correlation with tumor morphology. Mod Pathol. 2016;29:476–88.](http://paperpile.com/b/SxU1B4/XXKQ)

[24. Savage P, Pacis A, Kuasne H, Liu L, Lai D, Wan A, et al. Chemogenomic profiling of breast cancer patient-derived xenografts reveals targetable vulnerabilities for difficult-to-treat tumors. Commun Biol. 2020;3:310.](http://paperpile.com/b/SxU1B4/PBqT4)

[25. Shi J, Walker MG. Gene Set Enrichment Analysis (GSEA) for Interpreting Gene Expression Profiles. Curr Bioinform. 2007;2:133–7.](http://paperpile.com/b/SxU1B4/WVqsA)

[26. Liberzon A, Birger C, Thorvaldsdóttir H, Ghandi M, Mesirov JP, Tamayo P. The Molecular Signatures Database (MSigDB) hallmark gene set collection. Cell Syst. 2015;1:417–25.](http://paperpile.com/b/SxU1B4/fqZ83)

**Second round of review**

**Reviewer 1**

In the revised manuscript, the authors have properly addressed my concerns. One minor issue is that some gene symbols in Figure 4f are not clear.

**Reviewer 3**

Thanks for addressing my questions. I have no further comments.
